# Supplementary material for: Multiple origins of a frameshift insertion in a mitochondrial gene in birds and turtles
Source: Gigascience. 2021 Jan 19;10(1):giaa161. doi: 10.1093/gigascience/giaa161 (PMC7814300; doi:10.1093/gigascience/giaa161)
Supplement: giaa161_GIGA-D-20-00122_Original_Submission [file giaa161_giga-d-20-00122_original_submission.pdf]

GigaScience

Multiple origins of a frameshift insertion in a mitochondrial gene in birds and turtles

--Manuscript Draft--

**Manuscript Number:**	GIGA-D-20-00122
**Full Title:**	Multiple origins of a frameshift insertion in a mitochondrial gene in birds and turtles
**Article Type:**	Research
**Funding Information:**	Carlsbergfondet (DK) (CF16-0663) — Dr Guojie Zhang<br>Strategic Priority Research Program of the Chinese Academy of Sciences (XDB31020000) — Dr Guojie Zhang<br>Villum Fonden (25900) — Dr Guojie Zhang
**Abstract:**	Background<br><br>During evolutionary history, molecular mechanisms have emerged that can cope with deleterious mutations. Frameshift insertions in protein coding sequences are extremely rare because they disrupt the reading frame. There are a few known examples of their correction through translational frameshifting, which enables ribosomes to skip nucleotides during translation to regain proper reading frame. Corrective frameshifting has been proposed to act on the single base pair insertion at position 174 of the NADH dehydrogenase subunit 3 gene (ND3) that has been observed in a number of turtles and birds. However, the relatively sparse taxonomic representation has hampered our understanding on the evolution of this insertion in vertebrates.<br><br>Results<br><br>Here, we analyzed 94,518 ND3 sequences from 9,429 vertebrate species to reveal the phylogenetic history of this insertion and its common genomic characteristics. We confirmed that the insertion only appears in turtles and birds and reconstructed that it evolved independently with complex patterns of gains and losses. The insertion was observed in almost all bird orders. Surprisingly, the diverse Passeriformes have never regained the insertion since their origin in the Eocene. We showed strong conservation in the nucleotides surrounding the insertion in both turtles and birds with it, which implies that the insertion enforces structural constraints that could be involved in its correction.<br><br>Conclusions<br><br>Our study demonstrates that frameshifts can be widespread, with complex patterns of gains and losses within birds and turtles, and can be retained for millions of years if they are embedded in a conserved sequence theme.
**Corresponding Author:**	Sergio Andreu-Sánchez<br>Universitair Medisch Centrum Groningen<br>Groningen, Groningen NETHERLANDS
**Corresponding Author Secondary Information:**	
**Corresponding Author's Institution:**	Universitair Medisch Centrum Groningen
**Corresponding Author's Secondary Institution:**	
**First Author:**	Sergio Andreu-Sánchez
**First Author Secondary Information:**	
**Order of Authors:**	Sergio Andreu-Sánchez<br>Josefin Stiller

Powered by Editorial Manager® and ProduXion Manager® from Aries Systems Corporation

	Wanjun Chen
	Guojie Zhang
**Order of Authors Secondary Information:**	
**Additional Information:**	
**Question**	**Response**
Are you submitting this manuscript to a special series or article collection?	No
**Experimental design and statistics**<br><br>Full details of the experimental design and statistical methods used should be given in the Methods section, as detailed in our Minimum Standards Reporting Checklist. Information essential to interpreting the data presented should be made available in the figure legends.<br><br>Have you included all the information requested in your manuscript?	Yes
**Resources**<br><br>A description of all resources used, including antibodies, cell lines, animals and software tools, with enough information to allow them to be uniquely identified, should be included in the Methods section. Authors are strongly encouraged to cite Research Resource Identifiers (RRIDs) for antibodies, model organisms and tools, where possible.<br><br>Have you included the information requested as detailed in our Minimum Standards Reporting Checklist?	Yes
**Availability of data and materials**<br><br>All datasets and code on which the conclusions of the paper rely must be either included in your submission or deposited in publicly available repositories (where available and ethically appropriate), referencing such data using	No

Powered by Editorial Manager® and ProduXion Manager® from Aries Systems Corporation

a unique identifier in the references and in the “Availability of Data and Materials” section of your manuscript.<br><br>Have you have met the above requirement as detailed in our Minimum Standards Reporting Checklist?	
If not, please give reasons for any omissions below.<br><br>as follow-up to "**Availability of data and materials**<br><br>All datasets and code on which the conclusions of the paper rely must be either included in your submission or deposited in publicly available repositories (where available and ethically appropriate), referencing such data using a unique identifier in the references and in the “Availability of Data and Materials” section of your manuscript.<br><br>Have you have met the above requirement as detailed in our Minimum Standards Reporting Checklist?<br><br>"	All data will be deposited in GigaDB upon confirmation from the editor.

Powered by Editorial Manager® and ProduXion Manager® from Aries Systems Corporation

Manuscript Click here to download Manuscript Main_text.docx

Research

Multiple origins of a frameshift insertion in a mitochondrial gene in birds and turtles

Sergio Andreu-Sánchez[1,*]https://orcid.org/0000-0002-3503-9971, Wanjun Chen[2], Josefin Stiller[1]https://orcid.org/0000-0001-6009-9581, Guojie Zhang[1,2,3,4]https://orcid.org/0000-0001-6860-1521

[1] Villum Centre for Biodiversity Genomics, Section for Ecology and Evolution, Department of Biology, University of Copenhagen, Denmark
[2] China National Genebank, BGI-Shenzhen, Shenzhen, 518083, China
[3] State Key Laboratory of Genetic Resources and Evolution, Kunming Institute of Zoology, Chinese Academy of Sciences, Kunming, 650223, China
[4] Center for Excellence in Animal Evolution and Genetics, Chinese Academy of Sciences, 32 Jiaochang Donglu, Kunming 650223, China

*Current address: University of Groningen, University Medical Center Groningen, Department of Pediatrics, 9700 RB Groningen, Netherlands.

Corresponding author: guojie.zhang@bio.ku.dk

Abstract (250 words)

Background

During evolutionary history, molecular mechanisms have emerged that can cope with deleterious mutations. Frameshift insertions in protein coding sequences are extremely rare

because they disrupt the reading frame. There are a few known examples of their correction through translational frameshifting, which enables ribosomes to skip nucleotides during translation to regain proper reading frame. Corrective frameshifting has been proposed to act on the single base pair insertion at position 174 of the NADH dehydrogenase subunit 3 gene (ND3) that has been observed in a number of turtles and birds. However, the relatively sparse taxonomic representation has hampered our understanding on the evolution of this insertion in vertebrates.

Results

Here, we analyzed 94,518 ND3 sequences from 9,429 vertebrate species to reveal the phylogenetic history of this insertion and its common genomic characteristics. We confirmed that the insertion only appears in turtles and birds and reconstructed that it evolved independently with complex patterns of gains and losses. The insertion was observed in almost all bird orders. Surprisingly, the diverse Passeriformes have never regained the insertion since their origin in the Eocene. We showed strong conservation in the nucleotides surrounding the insertion in both turtles and birds with it, which implies that the insertion enforces structural constraints that could be involved in its correction.

Conclusions

Our study demonstrates that frameshifts can be widespread, with complex patterns of gains and losses within birds and turtles, and can be retained for millions of years if they are embedded in a conserved sequence theme.

Keywords: Programmed frameshift, sequence analysis, ancestral state reconstruction, mitochondrion, ND3

Background

Comparative analysis of molecular sequences across the diversity of life allows us to discover which molecular mechanisms have been conserved and which were modified throughout evolution. Insertions or deletions in protein coding genes are usually selected against because they result in frameshifts that destroy the amino acid sequence and result in dysfunctional proteins (Tse et al., 2010). Albeit rare, examples of corrective frameshifting exist, which enables ribosomes to regain the proper reading frame (Atkins et al., 2016). Programmed translational frameshifts have been characterized in viruses, retrotransposons, bacteria, yeast and in mammalian antizymes (reviewed in Farabaugh, 1996). Additionally, corrective frameshifting was proposed to be acting in the mitochondria of a range of animals (ants (Beckenbach et al., 2005), glass sponges (Rosengarten et al., 2008), oyster (Milbury & Gaffney, 2005), birds (Härlid et al., 1997; Mindell et al., 1998) and turtles (Mindell et al., 1998; Parham et al., 2006)), where +1 frameshift insertions have been reported in up to six different mitochondrially-encoded genes (Russell & Beckenbach, 2008).

In vertebrates, it was in ostrich (*Struthio camelus*) where the first frameshift insertion was described in the mitochondrially-encoded NADH dehydrogenase 3 (ND3) gene at position 174 (hereafter ND3-174+1) (Härlid et al., 1997). An extended investigation found that the ND3-174+1 insertion was present in a species of turtle and in many bird species (46/61 bird species) (Mindell et al., 1998). A later study focused on turtle mitogenomes (31 species) showed that the insertion was widespread in turtles (27/31) and likely present in their ancestor but lost two to three times within turtles, while being absent in crocodiles (2 species) and snakes (1 species) (Russell & Beckenbach, 2008). From this pattern of absence and presence it was suggested that the insertion could have been present in a common ancestor of turtles and birds (i.e. Archelosauria (Crawford et al., 2015) birds+crocodiles, and turtles) and subsequently lost in crocodiles, and within lineages of turtles and birds (Russell & Beckenbach, 2008). The insertion

was not observed in any other vertebrate lineage (Mindell et al., 1998). The relatively small number of species investigated in each vertebrate group limited the resolution of the evolutionary history of the insertion across the vertebrate tree of life. Denser sampling promises to provide additional insight into the distribution of the insertion in different lineages, and to determine not only the common features of the sequence theme that allow the insertion to remain in the genome, but also find deviations from a conserved sequence theme.

Three common sequence features that surround frameshift insertions were identified in the better known examples of frameshifts (*Ty1* and *Ty3* genes in yeast, antizyme gene in mammals, *prfB* in *E. coli*), namely a tRNA that enables the ribosome to “slip” on the ribosome P-site, a rare codon in the A-site promoting the stall and a commonly used codon in the +1 frame (Baranov et al., 2002). In mitochondrial frameshifts, the mechanism leading to a programmed corrective frameshift is not as well characterized but similarities in the DNA sequence structure surrounding mitochondrial frameshift insertions indicate that there may be a common mechanism across mitochondrial genes and organisms that facilitates the correction (Russell & Beckenbach, 2008). In ND3-174+1, the insertion is usually found in a codon CUN, where N is the insertion. When entering the P-site of the ribosome during translation, this CUN codon produces a wobble pairing with tRNA-Leucine (Russell & Beckenbach, 2008). The codon downstream of the insertion is usually AGU, a rare Serine codon, which enters the A-site (Russell & Beckenbach, 2008). The CUN in the P-site and the AGU in the A-site are thought to initiate the frameshift correction by causing a stall in decoding (Russell & Beckenbach, 2008). The AGU codon further forms the beginning of a 15 base pair long stem-loop RNA secondary structure (Mindell et al., 1998), which may enhance the stall (Russell & Beckenbach, 2008). Then, there are two possibilities, to keep the shifted reading frame, which would lead to an early termination of the protein (in birds ending after 207 bp instead of the usual 354 bp (Mindell et al., 1998)), or to produce a +1 frameshift, leaving out A-175 and thus recovering the regular

reading frame of the protein. The latter is preferred since GUA, the codon found in the corrected frameshift right after the insertion, has canonical Watson-Crick match with tRNA-Valine while the 0 reading frame there is the rare wobble-pairing to tRNA-Serine AGU codon (Russell & Beckenbach, 2008).

Here, we build on the abundance of mitochondrial sequence data available for vertebrates to study the evolution of the frameshift insertion in the mitochondrial protein coding gene ND3. We compiled ND3 sequences representing 9,429 vertebrate species and reconstructed the ancestral state of the ND3-174 position in different clades of Diapsida in order to trace the origin and transformation of the frameshift. We further investigate sequence conservation and codon usage patterns around the insertion site to identify potential common sequence motifs that may be associated with the absence or presence of the frameshift insertion. This large dataset provides improved resolution to understand the evolution of this phenomenon and the common sequence patterns that appear to be required for maintaining the programmed translational frameshift.

Data description

In this work we have compiled a total of 94,176 ND3 sequences from 9,429 vertebrate species. The ND3 sequences were extracted from larger mitochondrial fragments or full mitochondrial genomes downloaded from NCBI's GenBank and RefSeq databases. All RefSeq mitochondrial genomes were downloaded from the NCBI FTP site (ftp://ftp.ncbi.nlm.nih.gov/refseq/release/mitochondrion/, downloaded 2019-12-03), resulting in 5,325 mitochondrial genomes with the term 'Vertebrata' contained in the taxonomy, from which we could retrieve the ND3 sequence in 5,320 records. We searched for nucleotide sequences containing the ND3 gene on the GenBank nucleotide database (accessed 2019-12-11) with the query "NADH dehydrogenase subunit 3" AND Vertebrata[Organism] AND mitochondrion[filter]".

A total of 92,352 sequence records were downloaded using a custom script. From those, we could retrieve 88,880 records of ND3 sequences. We further included mitochondrial data from the second phase of the Bird 10,000 genome project (B10K), which produced 336 mitochondrial genomes as part of their whole genome sequences (Feng et al., in review). Of these mitochondrial genomes, 318 had ND3 sequences assembled, which were added if the species was not already present in the database. The records were further filtered to exclude sequences with an annotation 'UNVERIFIED', which GenBank adds when the accuracy of a sequence cannot be confirmed.

We reduced this dataset to one representative sequence for each species for the following analyses. We first checked if more than one ND3 sequences with both the insertion and without the insertion were present within the same species, which could be signs of intraspecific polymorphism, incorrect species identifications (i.e. multiple species are grouped under the same taxonomic name on NCBI), or errors during annotation. One species (*Otidiphaps nobilis*) had different nucleotides (2 sequences with C and one with T) present in the insertion. A total of 72 species had some sequences containing the insertion and some sequences without it (see Additional Table 1). It is hard to know if these cases reflect intraspecific polymorphism or annotation errors. We assume that it is more likely that these cases resulted from removal of the frameshift insertion through manual curation before sequence submission to NCBI. Frameshifts in coding regions occur rarely and may be removed as a presumed sequencing error if the curator was unaware of the possibility of a genuine frameshift insertion. Submission of a sequence that contains the frameshift insertion on the other hand requires specific annotation and hence knowledge of its existence in certain groups. We therefore assumed that for the 72 species in which both a gap and an insertion coexisted, it was more likely that the insertion was removed unknowingly, rather than the insertion being an artifact and coded the species as containing the insertion (Additional Table 1).

We then removed all intraspecific records giving sequences from RefSeq preference over GenBank sequences, and GenBank sequences over the B10K records. If more than one sequence for the same species were found among GenBank records, a random sequence was chosen. This resulted in a final dataset of a total of 9,429 vertebrate species.

A table including all 94,176 records, their accession number and the nucleotide identified at position 174 can be obtained Additional_Table_1. The multiple sequence alignment of the 9,429 unique vertebrates used is available in Additional_File_1.

Analysis

Studying the presence and absence pattern of ND3-174+1 in turtles and birds

Protein coding sequences for ND3 of a total of 9,429 vertebrate species were sourced from NCBI. A normal reading frame of ND3, without an insertion at position 174, was found in all included species of jawless fishes (Cyclostomata, 31 species included), cartilaginous fishes (Chrondrichthyes, 209), bony fishes (Actinopterygii, 3,340), coelacanths (Actinistia, 2), amphibians (Amphibia, 564), mammals (Mammalia, 1,443), snakes, lizards, worm lizards and tuatara (Lepidosauria 292 species) and crocodiles (Crocodylia, 22 species). Only some lineages in turtles (Testudines, 143 species) and birds (Aves 3,340 species) contained an insertion of a single nucleotide in position 174 (ND3-174+1). Thus, we focused further analyses on the group of Diapsida, from which we obtained a phylogenetic tree for 3,480 species, which includes Archelosauria (birds (3,066 species), crocodiles (22 species) and turtles (126 species)), and their sister group Lepidosauria (266 species). In Diapsida, no insertion was detected in 2,663

species, while ND3-174+1 was present in 817 species. The inserted nucleotide was cytosine (C) in 749 species, thymine (T) in 59, guanine (G) in 7 and adenine (A) in 2 species.

The insertion was only found in birds and turtles (Figure 1A) (labeled tips in Supplementary Figure 1). Within birds, the insertion was present in all bird orders except for the speciose perching birds (Passeriformes, 2,103 species included) and tropicbirds (Phaethontiformes, 2 species included). Within turtles, 87 of 126 examined species had the insertion and they were dispersed across the phylogeny (Figure 1A). We used maximum likelihood ancestral state reconstruction with an equal rates model to infer the marginal likelihoods of the 5 states (gap, A, T, C, G) at each of the internal nodes of the tree. A lack of the ND3-174+1 insertion (gap) was inferred as the ancestral state for the common ancestor of Diapsida, the common ancestor of lizards and snakes (Lepidosauria), and the common ancestor of birds and crocodiles (Archosauria), and the common ancestor of Archosauria and turtles (Archelosauria) with high probability (marginal likelihood of gap state > 0.99). The common ancestor of birds was inferred to have contained the insertion (marginal likelihood of C state 0.99). The common ancestor of turtles was reconstructed without the insertion (marginal likelihood of gap state 0.99). Across Diapsida, there were at least 38 gains of the insertion and 43 losses (Figure 1A). Most transitions were from C to T (28 transitions), while other state changes were less frequent (2 transversions from C to G, 1 C to A, 1 T to A).

The evolution of the insertion within turtles was complex with four inferred gains (marginal likelihood > 0.90), of which three were insertions of C and one was an insertion of T (Figure 1B). We inferred two losses of the insertion (always from an ancestral C state) (Figure 1B). Transitions from C to T were most common (7 transitions), compared to other transitions (2 T to C) and transversions (1 T to A). An insertion of G was observed in turtles in a clade of 3 species

(*Malaclemys terrapin, Trachemys scripta, Chrysemys picta* in Emysternia), but the ancestral state with their closest relatives could not be inferred unambiguously.

In birds, the ancestor of Passeriformes was inferred to have lost the insertion (marginal likelihood 0.99). Outside of Passeriformes (963 species), there were another 40 independent losses of the insertion, from a predicted ancestral C allele (marginal likelihood > 0.90), and 34 independent gains (30 gains of a C insertion and 4 gains of a T). As in turtles, transitions from transitions from C to T (21 transitions) were most common among non-passerine birds, with other state changes being less frequent (2 transversions from C to G, 1 C to A). Different orders of birds have different prevalence of the insertion or its absence (Figure 1C). The only two orders consistently without the frameshift insertion were Passeriformes and Phaethoniformes, while it was present in all examined species of 12 orders (Pterocliformes, Columbiformes, Mesitornithiformes, Musophagiformes, Otidiformes, Opisthocomiformes, Eurypygiformes, Suliformes, Cariamiformes, Falconiformes, Coliiformes, Trogoniformes). The remaining bird orders had both lineages with and without the insertion.

Sequence conservation analysis

In order to identify a potential shared sequence pattern around the insertion, we analyzed and compared the nucleotide diversity in ND3 sequences with and without the insertion at position 174. We calculated nucleotide diversity (as Shannon entropy) and information content (R) for each base pair. Information content quantifies the amount of information that is gained when a random sample is taken and has a maximum value (R=2 bits) if the position is fully conserved. In taxa without the insertion (Lepidosauria, crocodiles, certain turtles and certain birds), the region around position 174 (from 163 to 180 bp) of ND3 had a similar conservation level than other areas of the gene sequence (Figure 2A). In contrast, all species with the insertion (certain turtles and certain birds) had noticeably more conserved (higher R) sequences around position

174 than other regions of ND3 compared to species without the insertion (Figure 2A). The distribution of information content values (R) from position 163 to 180 was significantly different between species with and without the insertion (non-parametric Wilcoxon test, p-value 0.005), with a mean information content ratio between species with gap and species with insertion of 0.8 (Figure 2A). Specifically, when the insertion was present, there was a high conservation on upstream nucleotides of the insertion (position 163 to 174), with some variability on the third codon positions (Figure 2B). The sequence downstream of the insertion (position 175 to 180) were highly conserved with a maximum information content (R=2 bits, Figure 2B). When the insertion was absent, sequence conservation was lower (R<2 bits), particularly on the third codon positions and conservation downstream of position 174 (Figure 2C).

We also analyzed the codon conservation in sequences containing the insertion (a combined set of turtles and birds with the insertion). We analyzed both the 0 reading frame, which is the shifted reading frame in which the protein would be translated if the frameshift insertion was retained, and the +1 corrected reading frame, which is thought to leave out the adenosine at position 175 (A-175) (Russell & Beckenbach, 2008). In the 0reading frame, the first two codons downstream of the insertion showed almost complete conservation to AGT in codon +1 (encoding Serine) and AGC in codon +2 (encoding Serine) (Figure 2D). There were two species that did not contain AGT in the +1 codon. Baillon's crake (*Porzana pusilla*, Gruiformes) had a CGT codon, which encodes for Arginine, a basic amino acid. The Eastern black-bridged leaf turtle (*Cyclemys pulchristriata*, Geoemydidae) showed AAT, which encodes Asparagine, a neutral amino acid like Serine. Codon +2, which was AGC in 99.7% of the analyzed sequences, showed two non-synonymous variations changing the chemical properties of the amino acid, GGC (encoding for Glycine, non-polar aliphatic) in the Watercock (*Gallicrex cinerea*, Gruiformes), and CTC (encoding for Leucine, non-polar aliphatic) in the Eastern black-bridged leaf turtle (*Cyclemys pulchristriata*, Geoemydidae). Under the corrective frameshift model

leaving out A-175, the codon +1 after the insertion showed the greatest conservation (GTA in 99.7% of the sequences) encoding for the non-polar Valine (Figure 2D). Existing variation was synonymous (GTG) in the watercock and non-synonymous (ATC) in the Green wood hoopoe (*Phoeniculus purpureus*, Bucerotiformes) which encodes an Isoleucine, another non-polar amino acid (Figure 2D). The following codon +2 was also highly conserved in coding for Alanine, albeit with all four synonymous codons present (Figure 2D). One non-synonymous, but still non-polar amino acid (TCC) was a Serine in the Eastern black-bridged leaf turtle.

Discussion

In this work we have inferred multiple origins of an insertion in position 174 of the mitochondrially-encoded NADH dehydrogenase 3 complex gene (ND3-174+1) based on a large collection of publicly available sequences for 9,429 vertebrate species. This study significantly expands the sampling of previous studies (61 species in (8), 34 species in (10)) to provide a broader picture across vertebrates on one hand and more fine-scale resolution of sequence conservation on the other hand. We confirm that the insertion is present exclusively in turtles and birds (Mindell et al., 1998; Russell & Beckenbach, 2008) but the improved sampling shows that both the insertion was more frequently gained and lost than previously thought. Different from previous interpretations, which predicted the presence of this insertion in the common ancestor of turtles and birds (Archelosauria), our analyses suggested an independent evolution of this insertion in these two groups according to maximum likelihood ancestral state reconstruction under an equal-rates assumption of loss and gain. Within birds, the insertion was reconstructed as present in the most recent common ancestor of modern birds, which lived about 70-111 million years ago (depending on the phylogeny, (Jarvis et al., 2014; Prum et al., 2015)). Once obtained, the insertion was retained in many lineages but lost in the common ancestor of Passeriformes 39-49 million years ago (Jarvis et al., 2014; Oliveros et al., 2019;

Prum et al., 2015) and not regained since. Additionally, within the other bird lineages, it appeared more likely to lose the insertion than to gain it (40% of changes are insertion losses, while 34% are insertion gains). The most recent common ancestor of turtles was inferred to not have had an insertion in ND3, as opposed to previous ideas (Mindell et al., 1998; Russell & Beckenbach, 2008). Our data included 126 species of turtles, 95 more than in the last study on turtle mitochondrial genomes (Russell & Beckenbach, 2008), which produced an alternative interpretation of the gain and loss patterns. Within turtles, the insertion has been independently gained at least four times based on our reconstructions. Once the insertion is gained, some lineages show transitions to other nucleotides. The higher prevalence of C to T transitions both in turtles and birds (28/34, 82% of mutations) could be a consequence of cytosine methylation, which has been described to be present also in mitochondrial genomes (Sirard, 2019).

The quality of the ND3 sequences used here and the observed absence or presence of the insertion at position 174 is of crucial importance for our inferences. Most of the ND3 sequences used here originate from Sanger sequenced ND3 genes, whose chromatograms may have been hand-curated for sequencing errors. Insertions at position 174 are likely to be genuine, as they would have been flagged as problematic during submission to NCBI’s Genbank and would require a special annotation to address the frameshift insertion (often to the Mindell et al. study (Mindell et al., 1998)). The absence of the insertion on the other hand may be overrepresented in our dataset. A frameshift insertion in ND3 may have been curated out of the sequence because such insertions in the protein coding sequence are extremely rare and may therefore be considered a sequencing error and removed from the sequence. Given the prevalence of the ND3 insertion in certain clades, the number of loss events in turtles and non-Passeriformes birds could be an overestimation. Where losses were observed in multiple species of a clade, the most extreme case being the absence in all 2,103 included Passeriformes, the absence of

the insertion is likely to be genuine. To gain an understanding of the prevalence of this problem, we compared ND3 annotations for 148 bird species that both have Sanger sequenced ND3 sequences on NCBI and also high throughput sequenced mitochondrial genomes from the B10K project, for which we have created the annotations personally and can therefore exclude manual modification. Reassuringly, we found that most annotations matched between the two sources (143/148). The 5 cases of differing annotations were likely manual curations of NCBI records. Nonetheless, in all 5 cases, the unedited annotation was also supported by at least one other Sanger sequenced record (Additional Table 1). This assessment admittedly spans only a small fraction of the taxa investigated here but lends support that at least in birds annotation errors may be limited (3.4%). The possibility that the variability within a species is genuine needs further investigation with additional, unedited sequencing of ND3 sequences from multiple individuals.

It remains unknown why the ND3 insertion appears in birds and turtles, and whether the occurrence of this insertion is under neutral change or subjected to natural selection. However, it is intriguing that the insertion in exactly the same position of the ND3 gene at position 174 has independently evolved 38 times and in turtles and birds. This site-specificity points to some underlying common feature that causes the frameshift to occur in this position. One possibility is that there is an increased probability to produce indels in that specific position. On the other hand, insertions may appear at a normal rate but its surrounding region in the gene might have specific features that allow ribosomes to program the frameshift correction similar to the mechanisms described in other systems (Russell & Beckenbach, 2008). We found that ND3 sequences that contain the insertion in birds and turtles are highly conserved, despite their separate evolution for over 240 million years. Intriguingly, this conservation is not found in birds and turtles that do not have the insertion (Figure 2A) nor in the other diapsids without the

insertion, as expected with the degree of divergence of vertebrates. This points to strong sequence constraints that could be involved in correcting the insertion.

While our study confirmed the previous prediction on the presence of conserved features for sequences surrounding the insertion (Russell & Beckenbach, 2008), our extended sampling provides more detailed patterns have been missed with few sampling and a higher resolution of the sequencing conservation features that could be useful for understanding the molecular process of the programmed translational frameshift. The codon upstream of the frameshift insertion in ND3 is a Leucine codon (CTN) in all examined bird and turtle species with the exception of one bird. This upstream codon was proposed to produce a wobble pairing promoting the translation stall (Russell & Beckenbach, 2008). We further confirm that two AGY codons encoding for Serine, a polar amino acid, were almost always observed after the insertion (Russell & Beckenbach, 2008). However, our extended sampling shows that this pattern can be more flexible in both birds and turtles, with CGT (Arginine, also polar) in a bird and AAT (Asparagine, basic) in a turtle (Figure 2D). The consequences of this amino acid change on the programmed translational frameshift are unknown. It was previously hypothesized that the AGT codon following the insertion, a rarely used codon for Serine, promotes a stall in the translation which in turn facilitates the frameshift (Russell & Beckenbach, 2008). If the two observed variations, CGT or AAT, were also rare codons encoding for Arginine and Asparagine respectively, the hypothesis would still hold. Indeed, in human mitochondria CGT is the third least used codon out of four possible Arginine codons , while AAT is the least used Asparagine codon out of two possible codons (Jia & Higgs, 2008). If the codon usage was conserved across vertebrates, these rare codons may provide an alternative means to promote the stall in addition to the commonly used codon.

Regarding the insertion itself, we have observed all four nucleotides to be present in different frequencies. According to the frameshift model (Russell & Beckenbach, 2008), the insertion rarely occurs as an Adenosine. In addition to Reeve’s turtle (*Chinemys reevesi*), which has previously been shown to contain an A-insertion (Russell & Beckenbach, 2008), we found an independent occurrence of A-insertion in a bird, Baillon’s crake (*Porzana pusilla*), which also has a non-synonymous codon right after the insertion as described above. It is unusual to present this A since a CTA codon produces a perfect match with the tRNA-Leu anticodon (Russell & Beckenbach, 2008), which is in conflict with the idea that wobble pairing promotes frameshifting (Russell & Beckenbach, 2008). Baillon’s crake may therefore be an interesting model for further investigations on the programmed translational frameshift.

Our study demonstrates that incorporating a large number of sequences can improve resolution in inferred evolutionary patterns and give additional power to investigate sequence conservation and deviation from it. Our analyses suggested an independent origin both in turtles and birds, and complex patterns of gains and losses. The high sequence conservation surrounding the insertion suggests purifying selection retaining the sequence motifs needed for translational frameshifting. Nonetheless, a few species deviate from the conserved pattern. Additional losses and gains of the insertion and other deviations from the conserved motifs will likely be found once more ND3 sequences become available, within birds and turtles and possibly also in other vertebrates. In our current study we have roughly sampled 35% of turtle species (126/356) and 30% (3,065/10,021 (Edward C. Dickinson et al., 2004)) of birds, with approximatelly 34% of Passeriformes (2,103/6,063 (E. C. Dickinson & Christidis, 2014)). Nevertheless, much biodiversity remains to be sampled to provide further insight into the evolution and genomic setting that allows the insertion to persist.

Potential implications

We believe that the current work will help to advance our understanding of this corrective frameshift and its distribution across the vertebrate tree of life. This will allow researchers to further study which sequence features allow the programmed translational frameshift insertion to arise and to investigate the evolutionary constraints that keep the surrounding sequence heavily conserved. The fact that this insertion has remained in the mitochondrial genome for millions of years in certain birds and turtles opens the door to study the translational machinery in these lineages.

Methods

Sequence alignment

Full length ND3 sequences were aligned using MAFFT (Katoh & Standley, 2013). pxclsq from the phyx suite (Brown et al., 2017) was used to filter out positions of the alignment with over 95% of gaps (alignment is available in Additional File 1).

Phylogenetic distribution of the insertion

The frameshift insertion in position 174 was only observed in certain species of turtles and birds and we therefore restricted analyses to Diapsida, i.e. birds, crocodiles, turtles and Lepidosauria (tuatara, worm lizards, snakes and lizards). This left 3,797 out of 9,429 vertebrate species. We recorded the state of position 174 in each sequence, either being a gap (i.e. insertion absent), or being a nucleotide (i.e. insertion present as A,T,C,G).

We used the R package rotl (Michonneau et al., 2016) to obtain a phylogenetic tree for the included species. The package queries the Open Tree of Life (otol) database (https://tree.opentreeoflife.org/opentree/argus/opentree12.3@ott93302), which synthesizes phylogenetic hypotheses from published datasets and adds species that have not been included

in phylogenetic analyses based on the taxonomic system (Redelings & Holder, 2017; Rees & Cranston, 2017). While a fully sampled tree for Diapsida would be preferable over a synthetic tree, it agrees in the relationships among the major Diapsida clades with phylogenetic analyses (Chiari et al., 2012; Crawford et al., 2012; Green et al., 2014; Irisarri et al., 2017). Of the 3,797 diapsid species with ND3 records, 3,480 could be matched with a terminal on the Open Tree of Life (newick tree in Additional File 2, figure with named tips in Additional Figure 1). In order to summarize the distribution of absence or presence of the insertion on a dated bird phylogeny, we used the fossil-calibrated phylogenetic tree from (Jarvis et al., 2014).

Ancestral state reconstruction

We used the R package Castor (Louca & Doebeli, 2018) to obtain ancestral state likelihoods at each node of the phylogeny using the function hsp_mk_model. The function first calculates the transition matrix between different states assuming equal-rates for transitioning from one state to another and vice versa in a maximum likelihood framework. We chose the equal-rates model because it makes the least assumptions about the probabilities of gain, loss and transitions between different states. Given the known states of the tips and the phylogenetic tree, the marginal likelihood of each node in the tree was calculated using the rerooting method (Yang et al., 1995).

Inference of transition patterns

In order to specifically identify nodes of the phylogeny where transitions from one state to another occurred, we related the marginal likelihood from the ancestral state reconstruction of each descendant node to its parent node using the R package phangorn (Schliep, 2011). We only considered nodes with an ancestral state marginal likelihood> 0.9. If the marginal likelihood was < 0.9, the state was considered as ambiguous. A transition was counted when a

descendant node differed from its parent's state with high marginal likelihood. This approach therefore only identifies transitions that are accompanied with strong changes in likelihoods.

Sequence conservation and codon frequencies

Nucleotide frequencies per position across the entire ND3 sequence were obtained separately for diapsids without the insertion and for the diapsids with the insertion. We calculated Shannon entropy as a measure of nucleotide diversity (Mindell et al., 1998):

$$H_i = -\sum_N freq_N \ x \ log_2(freq_N)$$

where *H* is the Shannon entropy in position i of the DNA sequence, $freq_N$ is the frequency of nucleotide *N* of state {A,T,G,C}. Shannon entropy was transformed into information content per nucleotide position:

$R_i = log_2(4) - H_i$

where R is the information content at position i. Shannon entropy was compared between the two groups in the region surrounding the insertion (position 163-180) with a non-parametric Wilcoxon test and a significance threshold (α) of $p<0.05$. Weblogo (Crooks et al., 2004) was used to visualize the nucleotide diversity of this region (position 163-181) for both groups. The relative diversity versus conservation of each nucleotide was visualized as the height of the nucleotide, measured in bits with a maximum value of 2 at complete conservation.

Codon frequencies were calculated for both the shifted reading frame, the 0 reading frame, and for the corrected reading frame, the +1 reading frame. Codons containing unknown nucleotides (N) were removed. Codon frequencies were calculated for the 6 codons surrounding the insertion (position 172-181). The calculation of codon frequencies in the +1 reading frame excluded the Adenosine following the insertion A-175 (Russell & Beckenbach, 2008).

Availability of source code and requirements

Scripts used for data generation and analysis can be found at:

https://github.com/sergioSEa/ND3_174_vertebrates2020

Operating system(s): e.g. Bash scripts should be run in Linux OS/Mac OS. Python and R scripts are platform independent.

Programming language: Bash, R, Python

Other requirements: Python 3 or higher, Mafft v7.4, pxclsq 0.1. Python packages: biopython. R packages: Rotol, castor, ape, phytools, ggtree, ggimage, phangorn, ggstance, Biostrings, ggrepel and tidyverse.

License: GNU

Availability of supporting data and materials

The data sets supporting the results of this article are available in the [repository name, to be included once uploaded] repository, [Identifier]."

List of abbreviations

ND3 - NADH dehydrogenase 3 complex gene

Author’s contributions

Sergio Andreu-Sánchez: Formal analysis, writing, conceptualization; Wanjun Chen: data curation; Josefin Stiller: Conceptualization, supervision, visualization, writing; Guojie Zhang: Conceptualization; supervision; resources; writing

Acknowledgements

This project was supported by Carlsberg Foundation (CF16-0663). It was partially supported by the Strategic Priority Research Program of the Chinese Academy of Sciences (XDB31020000). GZ is also supported by Villum Foundation (No. 25900).

References

Atkins, J. F., Loughran, G., Bhatt, P. R., Firth, A. E., & Baranov, P. V. (2016). Ribosomal frameshifting and transcriptional slippage: From genetic steganography and cryptography to adventitious use. *Nucleic Acids Research*, *44*(15), 7007–7078.

Baranov, P. V., Gesteland, R. F., & Atkins, J. F. (2002). Recoding: translational bifurcations in gene expression. *Gene*, *286*(2), 187–201.

Beckenbach, A. T., Robson, S. K. A., & Crozier, R. H. (2005). Single Nucleotide +1 Frameshifts in an Apparently FunctionalMitochondrial Cytochrome b Gene in Ants of the Genus Polyrhachis. *Journal of Molecular Evolution*, *60*(2), 141–152.

Brown, J. W., Walker, J. F., & Smith, S. A. (2017). Phyx: phylogenetic tools for unix. *Bioinformatics* , *33*(12), 1886–1888.

Chiari, Y., Cahais, V., Galtier, N., & Delsuc, F. (2012). Phylogenomic analyses support the position of turtles as the sister group of birds and crocodiles (Archosauria). *BMC Biology*, *10*, 65.

Crawford, N. G., Faircloth, B. C., McCormack, J. E., Brumfield, R. T., Winker, K., & Glenn, T. C. (2012). More than 1000 ultraconserved elements provide evidence that turtles are the sister group of archosaurs. *Biology Letters*, *8*(5), 783–786.

Crawford, N. G., Parham, J. F., Sellas, A. B., Faircloth, B. C., Glenn, T. C., Papenfuss, T. J., Henderson, J. B., Hansen, M. H., & Simison, W. B. (2015). A phylogenomic analysis of turtles. *Molecular Phylogenetics and Evolution*, *83*, 250–257.

Crooks, G. E., Hon, G., Chandonia, J.-M., & Brenner, S. E. (2004). WebLogo: a sequence logo generator. *Genome Research*, *14*(6), 1188–1190.

Dickinson, E. C., Bahr, N., Dowsett, R., Pearson, D., Remsen, V., Roselaar, C. S., Schodde, D.,

& Others. (2004). *The Howard and Moore complete checklist of birds of the world*. https://dare.uva.nl/personal/pure/en/publications/the-howard-and-moore-complete-checklist-of-birds-of-the-world(75e7072c-f976-46ed-864e-5f806f313af0).html

Dickinson, E. C., & Christidis, L. (2014). *The Howard and Moore complete checklist of the birds of the World: Passerines*.

Farabaugh, P. J. (1996). Programmed translational frameshifting. *Microbiological Reviews*, *60*(1), 103–134.

Green, R. E., Braun, E. L., Armstrong, J., Earl, D., Nguyen, N., Hickey, G., Vandewege, M. W., St John, J. A., Capella-Gutiérrez, S., Castoe, T. A., Kern, C., Fujita, M. K., Opazo, J. C., Jurka, J., Kojima, K. K., Caballero, J., Hubley, R. M., Smit, A. F., Platt, R. N., … Ray, D. A. (2014). Three crocodilian genomes reveal ancestral patterns of evolution among archosaurs. *Science*, *346*(6215), 1254449.

Härlid, A., Janke, A., & Arnason, U. (1997). The mtDNA sequence of the ostrich and the divergence between paleognathous and neognathous birds. *Molecular Biology and Evolution*, *14*(7), 754–761.

Irisarri, I., Baurain, D., Brinkmann, H., Delsuc, F., Sire, J.-Y., Kupfer, A., Petersen, J., Jarek, M., Meyer, A., Vences, M., & Philippe, H. (2017). Phylotranscriptomic consolidation of the jawed vertebrate timetree. *Nature Ecology & Evolution*, *1*(9), 1370–1378.

Jarvis, E. D., Mirarab, S., Aberer, A. J., Li, B., Houde, P., Li, C., Ho, S. Y. W., Faircloth, B. C., Nabholz, B., Howard, J. T., Suh, A., Weber, C. C., da Fonseca, R. R., Li, J., Zhang, F., Li, H., Zhou, L., Narula, N., Liu, L., … Zhang, G. (2014). Whole-genome analyses resolve early branches in the tree of life of modern birds. *Science*, *346*(6215), 1320–1331.

Jia, W., & Higgs, P. G. (2008). Codon usage in mitochondrial genomes: distinguishing context-dependent mutation from translational selection. *Molecular Biology and Evolution*, *25*(2), 339–351.

Katoh, K., & Standley, D. M. (2013). MAFFT multiple sequence alignment software version 7:

improvements in performance and usability. *Molecular Biology and Evolution*, *30*(4), 772–780.

Louca, S., & Doebeli, M. (2018). Efficient comparative phylogenetics on large trees. *Bioinformatics* , *34*(6), 1053–1055.

Michonneau, F., Brown, J. W., & Winter, D. J. (2016). rotl : an R package to interact with the Open Tree of Life data. *Methods in Ecology and Evolution / British Ecological Society*, *7*(12), 1476–1481.

Milbury, C. A., & Gaffney, P. M. (2005). Complete mitochondrial DNA sequence of the eastern oyster Crassostrea virginica. *Marine Biotechnology* , *7*(6), 697–712.

Mindell, D. P., Sorenson, M. D., & Dimcheff, D. E. (1998). An extra nucleotide is not translated in mitochondrial ND3 of some birds and turtles. *Molecular Biology and Evolution*, *15*(11), 1568–1571.

Oliveros, C. H., Field, D. J., Ksepka, D. T., Barker, F. K., Aleixo, A., Andersen, M. J., Alström, P., Benz, B. W., Braun, E. L., Braun, M. J., Bravo, G. A., Brumfield, R. T., Chesser, R. T., Claramunt, S., Cracraft, J., Cuervo, A. M., Derryberry, E. P., Glenn, T. C., Harvey, M. G., … Faircloth, B. C. (2019). Earth history and the passerine superradiation. *Proceedings of the National Academy of Sciences of the United States of America*, *116*(16), 7916–7925.

Parham, J. F., Macey, J. R., Papenfuss, T. J., Feldman, C. R., Türkozan, O., Polymeni, R., & Boore, J. (2006). The phylogeny of Mediterranean tortoises and their close relatives based on complete mitochondrial genome sequences from museum specimens. *Molecular Phylogenetics and Evolution*, *38*(1), 50–64.

Prum, R. O., Berv, J. S., Dornburg, A., Field, D. J., Townsend, J. P., Lemmon, E. M., & Lemmon, A. R. (2015). A comprehensive phylogeny of birds (Aves) using targeted next-generation DNA sequencing. *Nature*, *526*(7574), 569–573.

Redelings, B. D., & Holder, M. T. (2017). A supertree pipeline for summarizing phylogenetic and taxonomic information for millions of species. *PeerJ*, *5*, e3058.

Rees, J. A., & Cranston, K. (2017). Automated assembly of a reference taxonomy for phylogenetic data synthesis. *Biodiversity Data Journal*, *5*, e12581.

Rosengarten, R. D., Sperling, E. A., Moreno, M. A., Leys, S. P., & Dellaporta, S. L. (2008). The mitochondrial genome of the hexactinellid sponge Aphrocallistes vastus: Evidence for programmed translational frameshifting. In *BMC Genomics* (Vol. 9, Issue 1, p. 33). https://doi.org/10.1186/1471-2164-9-33

Russell, R. D., & Beckenbach, A. T. (2008). Recoding of translation in turtle mitochondrial genomes: programmed frameshift mutations and evidence of a modified genetic code. *Journal of Molecular Evolution*, *67*(6), 682–695.

Schliep, K. P. (2011). phangorn: phylogenetic analysis in R. *Bioinformatics* , *27*(4), 592–593.

Sirard, M.-A. (2019). Distribution and dynamics of mitochondrial DNA methylation in oocytes, embryos and granulosa cells. *Scientific Reports*, *9*(1), 11937.

Tse, H., Cai, J. J., Tsoi, H.-W., Lam, E. P., & Yuen, K.-Y. (2010). Natural selection retains overrepresented out-of-frame stop codons against frameshift peptides in prokaryotes. *BMC Genomics*, *11*, 491.

Yang, Z., Kumar, S., & Nei, M. (1995). A new method of inference of ancestral nucleotide and amino acid sequences. *Genetics*, *141*(4), 1641–1650.

Additional Files

Additional Figure 1. Phylogenetic tree of 3,480 Diapsida species as in Figure 1A of the main text but plotted with species labels. Terminals are colored according to the status at position 174.

Additional Figure 2. Transition probabilities in turtles and birds. Only nodes with ancestral state marginal likelihood > 0.9 were used. Numbers refer to the frequency of a given transition from the overall transition events.

Additional File 1. Multiple sequence alignment of 9,429 vertebrate species. Positions over 95% gaps were removed.

Additional File 2. Newick tree of 3,480 Diapsida species.

Additional Table 1. Table of 94,176 vertebrates included in the study. *Latin_name* refers to the name of the species studied, *accession* is the NCBI accession ID, *source* refers whether the record was obtained from Genbank, Refseq or mitochondrial genome assemblies of the B10K project, *included* refers to the unique species used in downstream analysis, *status* is the

nucleotide found at position 174 of the alignment.

Figures and legends

Figure 1. Phylogenetic distribution of the insertion in position 174 of the mitochondrial ND3 gene. (a) Phylogeny of 3,480 species of Diapsida with terminals colored according to the absence (orange) or presence (blue) of the insertion. (b) Ancestral state reconstruction for turtles with states of species indicated at the tips and pie charts showing the marginal likelihoods of different states on nodes of the phylogeny. (c) Frequency of states (absence or presence of the insertion) in each bird order. Numbers represent the number of species that were analyzed for each order.

Figure 2. **Nucleotide and codon usage variability in ND3 of Diapsida.** (a) Information content R (cubed for visualization) across the ND3 sequence in different diapsid groups. The vertical red line marks the insertion at position 174. The red shading highlights an area of high conservation (high information content) only seen in birds and turtles that have the insertion. (b-c) Sequence conservation as a sequence logo from position 163 to 180 showing variability among species (b) with the insertion and (c) without the insertion. Note that the frameshift correction is thought to occur at the following base, by skipping the nucleotide A at position 175. (d) Circle packing showing the frequency of codon usage for each codon in species that contain the insertion. The two options of the shifted and corrected reading frame following the insertion at position 174 are shown. Circle diameters indicate prevalence of a specific codon, which are grouped into larger circles if codons are synonymous. Circle color indicates amino acid class.

Figure1 Click here to download Figure Figure1.pdf

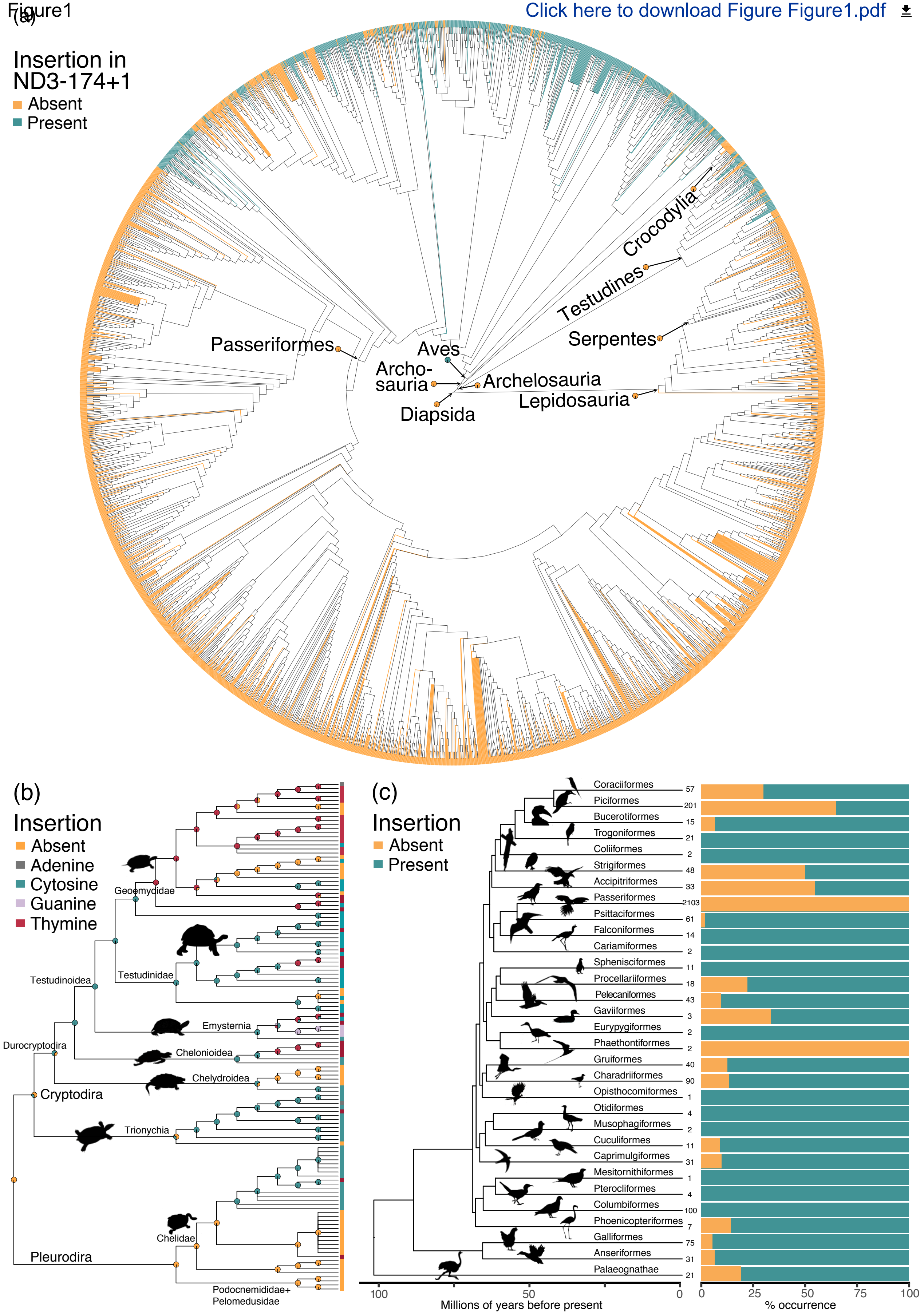

Figure2 Click here to download Figure Figure2.pdf

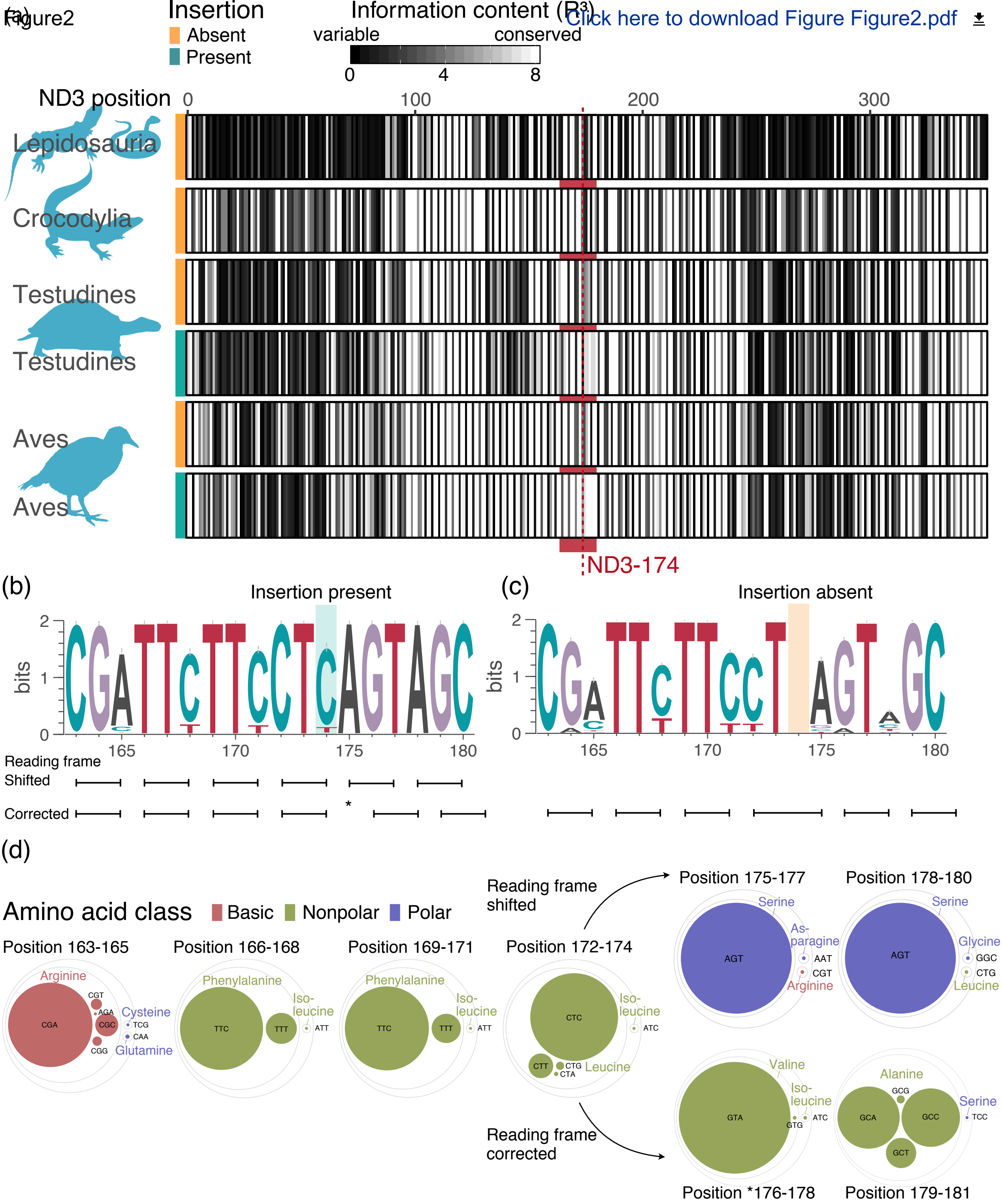

Supp_figure1

Click here to access/download
Supplementary Material
Supplementary_Figure_1.pdf

Supp_figure2

Click here to access/download
Supplementary Material
Supplementary_Figure_2.pdf

Supp_file1

Click here to access/download
Supplementary Material
Supplementary_File_1.txt

Supp_file2

Click here to access/download
Supplementary Material
Supplementary_File_2.txt

Click here to access/download
Supplementary Material
Supplementary_Table_1.txt
