# Supplementary material for: Multiple origins of a frameshift insertion in a mitochondrial gene in birds and turtles
Source: Gigascience. 2021 Jan 19;10(1):giaa161. doi: 10.1093/gigascience/giaa161 (PMC7814300; doi:10.1093/gigascience/giaa161)
Supplement: giaa161_GIGA-D-20-00122_Revision_1 [file giaa161_giga-d-20-00122_revision_1.pdf]

GigaScience

Multiple origins of a frameshift insertion in a mitochondrial gene in birds and turtles

--Manuscript Draft--

Manuscript Number:	GIGA-D-20-00122R1
Full Title:	Multiple origins of a frameshift insertion in a mitochondrial gene in birds and turtles
Article Type:	Research
Funding Information:	Carlsbergfondet (DK) (CF16-0663) — Dr Guojie Zhang<br>Strategic Priority Research Program of the Chinese Academy of Sciences (XDB31020000) — Dr Guojie Zhang<br>Villum Fonden (25900) — Dr Guojie Zhang

Abstract:

Abstract

Background

During evolutionary history, molecular mechanisms have emerged to cope with deleterious mutations. Frameshift insertions in protein coding sequences are extremely rare because they disrupt the reading frame. There are a few known examples of their correction through translational frameshifting, a process which enables ribosomes to skip nucleotides during translation to regain proper reading frame. Corrective frameshifting has been proposed to act on the single base pair insertion at position 174 of the mitochondrial NADH dehydrogenase subunit 3 gene (ND3) that has been observed in several turtles and birds. However, the relatively sparse taxonomic representation has hampered our understanding on the evolution of this insertion in vertebrates.

Results

Here, we analyzed 87,707 ND3 sequences from 10,308 vertebrate taxa to reveal the evolutionary history of this insertion and its common genomic characteristics. We confirmed that the insertion only appears in turtles and birds and reconstructed that it evolved independently in both groups with complex patterns of gains and losses. The insertion was observed in almost all bird orders but was absent in all members of the diverse Passeriformes. We found strong conservation in the nucleotides surrounding the insertion in both turtles and birds, which implies that the insertion enforces structural constraints that could be involved in its correction.

Conclusions

Our study demonstrates that frameshifts can be widespread, with complex patterns of gains and losses within birds and turtles and can be retained for millions of years if they are embedded in a conserved sequence theme.

Corresponding Author:	Sergio Andreu-Sánchez<br>Universitair Medisch Centrum Groningen<br>Groningen, Groningen NETHERLANDS
Corresponding Author Secondary Information:	
Corresponding Author's Institution:	Universitair Medisch Centrum Groningen
Corresponding Author's Secondary Institution:	
First Author:	Sergio Andreu-Sánchez
First Author Secondary Information:	
Order of Authors:	Sergio Andreu-Sánchez
	Josefin Stiller
	Wanjun Chen
	Guojie Zhang

Powered by Editorial Manager® and ProduXion Manager® from Aries Systems Corporation

Order of Authors Secondary Information:	
Response to Reviewers:	Reviewer #1:<br><br>>>>We thank the reviewer for their constructive suggestions, which have improved the manuscript. We have addressed all suggestions in detail below.<br><br>Reviewer #1: The manuscript "Multiple origins of a frameshift insertion in a mitochondrial gene in birds and turtles" by Andreu-Sánchez and colleagues is a well-written and very interesting manuscript. I think it is an important contribution. Programmed frameshifts are actually reasonably common in viruses (Harger et al. 2002; Plant et al. 2005; Brierley & Dos Ramos 2006) but quite rare in cellular organisms (in fact, a -1 frameshift exists in coronaviruses; Plant et al. 2005). The authors correctly cite much of the literature, including the existence of some other programmed frameshifts in mitochondrially-encoded proteins in metazoa, although it would be nice if the authors could cite some additional literature, like the more recent review by Dinman (2012). I only have a few relatively minor comments.<br>>>>We have now cited the suggested reference (L. 109).<br><br>To my knowledge, after the original Mindell et al. (1998) study, the only previous study that has explicitly pointed out homoplasy in the frameshift was one from my lab (Tamashiro et al. 2019). In both cases the distribution of frameshifts could be explained by an ancient origin combined with multiple losses.<br><br>I also note that the authors state (on lines 315-317) that "Most of the ND3 sequences used here originate from Sanger sequenced ND3 genes, whose chromatograms may have been hand-curated for sequencing errors. Insertions at position 174 are [therefore] likely to be genuine…". The Tamashiro et al. (2019) study I alluded to used Illumina data and I can definitely attest to the presence of the insertion in many reads (and the absence in the three taxa in that study that lack the frameshift insertion). My lab group has also collected a number of Sanger ND3 sequences and they definitely have the insertion. Errors are certainly possible (indeed, the original Desjardins & Morais 1990 chicken mitogenome sequence almost certainly removed the frameshift, assuming it was a sequencing error - Mindell et al. 1998 states this and other Gallus mitogenome sequences - including some from my lab - have the frameshift).<br>>>>We apologize if our phrasing caused any confusion. Our point was that, in case of an observed absence of the insertion from sequences available on Genbank, the insertion could have been mistakenly removed if the curator was not aware that an insertion could have been present. It is possible that curators that are unaware of the Mindell et al. 1998 study and others will be more likely to remove the insertion. The absence of an insertion in Genbank sequences is therefore not necessarily the true state. On the other hand, an insertion that is present at position 174 in sequences available on Genbank is more likely to be genuine as it would have been flagged as a frameshift insertion during the sequence submission process. Thus, a present insertion is therefore more likely to be the true state.<br><br>I only have one conceptual issue with the analyses. I think it would be good to complement the likelihood with a parsimony analysis. The conservative nature of the way the authors calculated the number of transitions (i.e., only counting changes between ancestral state reconstructions when the contribution of a specific state to the marginal likelihoods was >0.9) is a double-edged sword. After all, there will be cases where it is clear transformations have occurred, but the exact branch where that change occurred is unclear. I think a very simple parsimony analysis would complement their likelihood analysis and provide a useful way of reporting the results. Although parsimony is often considered "old-fashioned" in the phylogenetic community it is simply the likelihood solution given the no common mechanism model (Steel & Penny 2000); I am not convinced that the simple stochastic model used by the authors is better for an still poorly-characterized type of evolutionary change (like shifts between the presence and absence of a programmed frameshift) than the no common mechanism (parsimony) model. Indeed, the absence of changes within passerines suggests that the rate of change for the presence or absence of a frameshift is<br>>>>We thank the reviewer for this suggestion and we have incorporated a maximum parsimony analysis as implemented in the R ape package. The parsimony analysis

largely corroborates the likelihood analysis. Most nodes that have a high probability of one state in the likelihood analysis are also resolved in the parsimony analysis. In the same vein, nodes with uncertainty in the maximum likelihood model were also not resolved in the parsimony analysis and had equally parsimonious states of absence/presence. Corresponding sections have been added to the Results in Sections: Patterns of presence and absence of ND3-174+1 in vertebrates, Complex patterns of gain and loss within turtles and birds and Materials and methods in Section:Ancestral state reconstruction.

From the standpoint of mechanics, all the authors need to do is created a nexus file with a single binary character the program PAUP* (Swofford 2020) and then map the character on the trees the authors used. They already have the character scorings, so this should be a trivial file format conversion. If the authors read the tree, start saving a log file, and then issue the command "DescribeTrees / chglist=yes plot=no;" (or choose the same options through the GUI), and then stop logging. The can then simply use grep to count numbers of 0 to 1 and 1 to 0 changes (PAUP* reports unambiguous and ambiguous mapping of the changes using ==> and -->). This will run quickly (even on a laptop computer) and authors can report numbers of changes given ACCTRAN and DELTRAN (Swofford & Maddison 1987). Changing between the two optimizations is accomplished by "PSet opt=delTran;" and "PSet opt=accTran;" (note that ACCTRAN is the default). This will be a simple and easy to interpret analysis
that is insensitive to branch lengths. Assuming the authors add this simple analysis, they should also report the retention index (which is echoed when they do "DescribeTrees").

As stated above, one concern that leads me to suggest the parsimony analysis is the issue of branch lengths. I was unable to find how the authors obtained the branch lengths for their analysis. This should be clarified.
>>>We apologize that it was not explicitly stated. The used consensus phylogeny from the Open Tree of Life does not contain branch lengths. The model used assumed equal length for all branches. This is now clearly stated in on line 221.:
"Because the Open Tree of Life consensus phylogeny did not include branch lengths, the function assumes equal branch lengths throughout the phylogeny."

A final comment about parsimony: although I would really like to see the parsimony analysis, I am not convinced a parsimony analysis with five-state coding (i.e., coding the identity of the base) is necessary. I am mostly interested in the parsimony estimate of gains vs losses in a binary sense. Obviously, if the authors are interested in doing the five-state parsimony analysis it would be fine, I just feel the presence vs absence analysis is more interesting to readers.
>>>We followed the suggestion and reconstructed the presence/absence of the insertion in the maximum parsimony framework.

On lines 430-431 the authors state "It remains unknown why the ND3 insertion appears in birds and turtles, and whether the occurrence of this insertion is under neutral change or subjected to natural selection." This is awkwardly phrased. It might be better as "It remains unknown whether the ND3 insertion has any functional in birds and turtles; the gain and loss of the inserted nucleotide could be neutral or it could be subject to selection." I would also say that the hypothesis that indel mutations are especially common at this site (articulated on lines 434-435 "One possibility is that there is an increased probability to produce indels in that specific position") is certainly possible but it seems very unlikely. The possibility that frameshifts are tolerated in this position is much more likely. In this context, it would seems likely that there is a change in passerines that does not allow frameshifting (of course, it is possible that some change in passerines has resulted in a lower rate new indels mutations at this position, but it seems much more likely that new indels are not tolerated at this position and therefore removed by natural selection).

>>>Fixed as suggested (L. 401):
"One possibility is that there is an increased probability to produce indels in that specific position. Alternatively, insertions may appear at a normal rate but only get tolerated if they are embedded in a specific sequence motif that allows ribosomes to

conduct the frameshift correction. This seems likely given the strong conservation at the nucleotide and at the codon level that evolved convergently in birds and turtles, despite their separate evolution for over 240 million years.”

Finally, it would be a good idea for the authors to take more care with references. For example, on line 288 the authors use numbered references and, on line 320, "Mindell et al." is reiterated. Overall, a careful readthrough to catch these issues is warranted.

>>>Fixed.

Regardless of whether I provide a positive or negative review I like to sign my reviews (unless it is against journal policy). I believe the review process would be more positive and constructive if all reviews were either open or if they were double-blind; anonymous reviews create too much potential for reviews that are not constructive. Of course, it is much more pleasant to sign a review when I am enthusiastic about the manuscript, as I am here.
>>>We thank the reviewer for their constructive and positive comments, which have improved the quality of the manuscript.

Edward L. Braun

Reviewer #2:

>>>We thank the reviewer for their constructive and helpful comments. We appreciate the time and effort in scrutinizing the dataset and have addressed all suggestions and concerns in detail below.

Reviewer #2: The authors make use of a comprehensive mitogenomic dataset, covering a wide range of taxa among vertebrates, and later focusing on turtles and birds, to study the evolution of an insertion causing a frame-shift and an early stop codon in the mitochondrial ND3 gene. They identify it as occurring only in turtles and birds but being absent in Crocodilians and other groups of vertebrates. Using sequences for more than 9,000 taxa, they conduct a comparative phylogenetic approach and suggest that the insertion appeared at the ancestor of Archosauria and was later lost in Crocodilians as well as in multiple lineages of both birds and turtles. They also analyze the influence of base composition in the flanking regions of the insertion to examine their potential influence on the occurrence of the insertion and the mechanisms to cope with it.

The manuscript is general well written and easy to understand, even if some sections could be more concise and less redundant. It would be highly appreciated if the authors included some more information on the alternative mechanisms that have been proposed to deal with frame-shifts (see refs below).
>>>We thank the reviewer for the positive comments on the manuscript. We have paid careful attention in reducing redundancy throughout the manuscript.

The manuscript now mentions two other mechanisms other than programmed frameshifting with the relevant references (Line 113):
“Alternatively, other mechanisms could explain the absence of functional consequences of the frameshift insertion, such as non-canonical translation of tetra or penta codons, which might be an ancient translation mechanism [12], or RNA editing [13]. “

In addition, we also make a brief mention to the 3 translational frameshifts described in the introduction of Han et al (line 125):
"Three models have been proposed to enable translational frameshifting [13]. The "pause-and-slip" model proposes that a pause is induced at the A-site of the ribosome and that the P-site tRNA can pair with the +1 codon, allowing it to slip out of frame [15]. A second model proposes that abnormal tRNA structures enable the frameshift [16]. The "out-of-frame" model proposes that the recruited tRNA skips the additional nucleotide in the A-site [11]."

The methods section is in its current state well written and in detail described, the analytical pipeline is sound and the results seemed to support the conclusions presented by the authors.
For the discussion, however, I could not avoid the feeling that what they find is not different from what was already described in previous studies, which is a loss of the insertion in crocodilians and multiple losses and gains within turtles and birds. I would thus suggest, making use of the outstanding dataset the authors already compiled, the study to be extended to other insertions detected in the ND3 gene across vertebrates and, more particularly, in the taxa they focus, turtles and birds.
>>>We thank the reviewer for this suggestion. We have now studied the presence and absence of other insertions in ND3 in birds and turtles. We identified four frameshift insertions upstream of ND3-174 in five turtle species. Despite having many more available records from birds than from turtles, we did not detect any additional frameshifts in birds. This implies a widespread ability of coping with frameshifts in turtles, while translation frameshifting seems to be limited to ND3-174 in birds. Because of the limited occurrence of the other frameshifts, we focus the remaining manuscript on ND3-174. The corresponding section has been added to the manuscript, Section: Additional potential frameshifts in five turtle species

Before doing that, however, the authors need to go some stepes backwards and revise the alignment (provided in Additional file 1) as a close inspection of it led me to detected an issue that might require most of the analyses to be redone and the manuscript modified accordingly if the conclusions were to change.

First, the alignment needs to be improved to ensure that reading frame is respected along the full set of sequences along the whole gene. This is easily accomplished using a codon-aware aligner able to deal with frame-shifts. From my experience, the alternative proposed by Ranwez et al (2011) in MACSE (available at https://bioweb.supagro.inra.fr/macse/index.php?menu=releases) should be enough to correct this.
>>>We thank the reviewer for the suggestion of using a codon-aware aligner. Unfortunately, MACSE could not be used on the large alignment of 10,308 sequences across vertebrates used in our manuscript. We therefore decided to use MACSE on a smaller dataset across Diapsida (1,044 sequences). For the full Vertebrata alignment, we continued to use MAFFT but excluded sequences that could have resulted in spurious alignments around position 174. We filtered out sequences that were not properly aligned (e.g. large scale insertions or deletions which could be indicative of a numt or low-quality sequencing) or that had low quality in the region of interest (N in the codons surround position 174).

In order to assess the impact of the chosen aligner on the inference of the presence or absence of the insertion at ND3-174, we compared the prediction of presence/absence of the insertion at ND3-174 using the MACSE alignment of 1,044 Diapsida sequences with the MAFFT alignment of Diapsida derived from the Vertebrata alignment. We only observed different predictions in three species of a turtle genus, Cuora aurocapitata, Cuora pani and Cuora trifasciata. All three were predicted with no insertion by MACSE but with an insertion of T at position 174 in MAFFT. The MAFFT alignment shows a gap six base pairs upstream (see Reviewer 2 – Figures alignment A)

If the sequence was shifted to the left, the alignment would still be good and would remove the inferred insertion of T at position 174 (see screenshot below). We manually corrected this alignment: see Reviewer 2 – Figures alignment B

The choice of the aligner therefore did not influence much for the inference of the insertion or the inferred patterns of the gain and loss of the insertions. A heavier impact likely lies in the quality of the aligned sequences, which we have exposed to greater scrutiny now than in the previous iteration of the dataset.

Second, and more important, it is known for some species not having the insertion at position 174, that they can present a different one earlier in the sequence. An example of this, already presented in Russell & Beckenbach (2008), is the African helmeted turtle, Pelomedusa subrufa, which has not one but three different insertions, each on a different gene, including ND3.
When checking for this taxon, I noticed that (i) it is present twice in the alignment, likely due to the comma present in one of the labels, that likely made it escape the filtering to keep single sequences per taxon; perhaps this is also true for other taxa, thus adding redundant information ; (ii) this insertion is absent in the alignment and its incorrect filtering (maybe it is present in less than 95% of the sequences?) results in disrupted reading frames for the rest of the species.

In order to verify, I checked the mitogenome in Genbank (NC_001947.1), where the insertion is explicitly annotated as an exception and present in the annotated sequence (see captions of nucleotide and protein alignments in joint pdf file).
I consider that including it, along with the possibility that it happens elsewhere in the phylogeny, is susceptible to change the big picture of the evolution of insertions in this gene and should be addressed in this manuscript.

>>>We thank the reviewer for highlighting these problems with the included sequences. We have re-collected the dataset from scratch and have changed our strategy of collecting the sequence names to avoid the highlighted problems mentioned by the reviewer. For the previous dataset, we extracted species names from the Genbank headers but this was not always successful due to often non-standardized headers and resulted in artifacts such as the one described in (i). We now use NCBI's taxonomy to select unique sequences using the Organism field from the NCBI allowing for better control of taxon names. While this mostly corresponds to species names, it should be noted that NCBI's taxonomy sometimes includes placeholder taxa (e.g. species with cf. designation or non-formal names). We decided to include these placeholder taxa, which may be formally named at some point. This dataset is also more transparent as to which sequences are represented than our previous attempt to assign each sequence to a species.

For point ii), the absence of this particular frameshift was indeed caused by the filtering of positions not seen at least in 5% of sequences. We removed such singletons from the alignment to focus on position 174 but we acknowledge that this alignment alone is too simplified. We now include the unfiltered alignment Reviewer_2_alignment.txt (which will be uploaded to GigaDB) and the MACSE alignment where this insertion, and other possible frameshifts (Additional_File_4).

I think it would also be interesting some analyses on tRNAs, as coping with the frame-shifts described in this manuscript could be reflected in tRNA structures (see, for example, Haen et al (2014)). This option is not mentioned in the manuscript and given that some species present more than one insertion in coding genes along the mitogenome, it should definitively be considered. Given that a significant portion of the sequences in this study comes from fully assembled and annotated mitogenomes (e.g. RefSeq sequences from Genbank), having a look for differences in tRNAs sequences and structures should be feasible and would provide more value and support to the inferred trends and observations.
>>>This is an excellent suggestion. We have included an analysis of tRNA secondary structures in the codons surrounding the insertion. We compared secondary structures for birds and turtles with and without the insertion, respectively. We focused on the secondary structures of tRNAs of the codon encompassing the insertion (leucine) and the following tRNA, which is serine when the frameshift is not corrected and valine if

	the frameshift is corrected. The secondary structures between these groups were not noticeably different between the groups, which indicates that there are no convergent modifications to the tRNA secondary structures between birds and turtles with the insertion. The predicted secondary structures are included as Additional_file_6 and alignments will be made publicly available in GigaDB. The analyses are described in the Methods (Section:tRNA secondary structure prediction) and Results (Section: No major tRNA changes in taxa presenting the insertion).
Additional Information:	
Question	Response
Are you submitting this manuscript to a special series or article collection?	No
**Experimental design and statistics**<br><br>Full details of the experimental design and statistical methods used should be given in the Methods section, as detailed in our Minimum Standards Reporting Checklist. Information essential to interpreting the data presented should be made available in the figure legends.<br><br>Have you included all the information requested in your manuscript?	Yes
**Resources**<br><br>A description of all resources used, including antibodies, cell lines, animals and software tools, with enough information to allow them to be uniquely identified, should be included in the Methods section. Authors are strongly encouraged to cite Research Resource Identifiers (RRIDs) for antibodies, model organisms and tools, where possible.<br><br>Have you included the information requested as detailed in our Minimum Standards Reporting Checklist?	Yes
**Availability of data and materials**<br><br>All datasets and code on which the conclusions of the paper rely must be either included in your submission or deposited in publicly available repositories	No

Powered by Editorial Manager® and ProduXion Manager® from Aries Systems Corporation

(where available and ethically appropriate), referencing such data using a unique identifier in the references and in the "Availability of Data and Materials" section of your manuscript.<br><br>Have you have met the above requirement as detailed in our Minimum Standards Reporting Checklist?	
If not, please give reasons for any omissions below.<br><br>as follow-up to "**Availability of data and materials**<br><br>All datasets and code on which the conclusions of the paper rely must be either included in your submission or deposited in publicly available repositories (where available and ethically appropriate), referencing such data using a unique identifier in the references and in the "Availability of Data and Materials" section of your manuscript.<br><br>Have you have met the above requirement as detailed in our Minimum Standards Reporting Checklist?<br><br>"	All data will be deposited in GigaDB upon confirmation from the editor.

Manuscript
Click here to access/download;Manuscript;Manuscript.docx

Dear Hans Zauner,

We are pleased to resubmit our manuscript entitled “Multiple origins of a frameshift insertion in a mitochondrial gene in birds and turtles” (GIGA-D-20-00122) by Sergio Andreu-Sánchez, Wanjun Chen, Josefin Stiller and Guojie Zhang, to be considered for publication as an Research article in *GigaScience.*

We are grateful for the time and effort spent by the two reviewers to make helpful comments and suggestions on our manuscript. We have addressed all comments and outline our responses in the responses to the reviewers. Briefly, we have revised the manuscript with particular eye to filtering the underlying alignment of potential problematic sequences, have included an investigation of the secondary structure of tRNAs and have added a maximum parsimony analysis that corroborates the maximum likelihood reconstruction of ancestral states of the frameshift insertion.

We include a version of the manuscript that tracks changes and a version that has all changes accepted for better readability. We hope you will find the revised manuscript suitable for consideration for publication and are looking forward to hearing from you.

The authors declare no competing interests. All authors have approved the manuscript for submission. The content of the manuscript has not been published, or submitted for publication elsewhere.

Sincerely,

Guojie Zhang, Ph.D.
Professor
Department of Biology
University of Copenhagen
2100 København Ø, Denmark
Phone, office：+45 91 85 54 31
E-mail: guojie.zhang@bio.ku.dk

Research

Multiple origins of a frameshift insertion in a mitochondrial gene in birds and turtles

Sergio Andreu-Sánchez[1,*]https://orcid.org/0000-0002-3503-9971**, Wanjun Chen**[2]**, Josefin Stiller**[1]https://orcid.org/0000-0001-6009-9581**, Guojie Zhang**[1,2,3,4]https://orcid.org/0000-0001-6860-1521

[1] Villum Centre for Biodiversity Genomics, Section for Ecology and Evolution, Department of Biology, University of Copenhagen, Denmark
[2] BGI-Shenzhen, Beishan Industrial Zone, Shenzhen 518083, China
[3] State Key Laboratory of Genetic Resources and Evolution, Kunming Institute of Zoology, Chinese Academy of Sciences, Kunming, 650223, China
[4] Center for Excellence in Animal Evolution and Genetics, Chinese Academy of Sciences, 32 Jiaochang Donglu, Kunming 650223, China

*Current address: University of Groningen, University Medical Center Groningen, Department of Pediatrics, 9700 RB Groningen, Netherlands.

Corresponding author: guojie.zhang@bio.ku.dk

Abstract

Background

During evolutionary history, molecular mechanisms have emerged to cope with deleterious mutations. Frameshift insertions in protein coding sequences are extremely rare because they disrupt the reading frame. There are a few known examples of their correction through translational frameshifting, a process which enables ribosomes to skip nucleotides during translation to regain proper reading frame. Corrective frameshifting has been proposed to act on the single base pair insertion at position 174 of the mitochondrial NADH dehydrogenase subunit 3 gene (ND3) that has been observed in several turtles and birds. However, the relatively sparse taxonomic representation has hampered our understanding on the evolution of this insertion in vertebrates.

<u>Results</u>

Here, we analyzed 87,707 ND3 sequences from 10,308 vertebrate taxa to reveal the evolutionary history of this insertion and its common genomic characteristics. We confirmed that the insertion only appears in turtles and birds and reconstructed that it evolved independently in both groups with complex patterns of gains and losses. The insertion was observed in almost all bird orders but was absent in all members of the diverse Passeriformes. We found strong conservation in the nucleotides surrounding the insertion in both turtles and birds, which implies that the insertion enforces structural constraints that could be involved in its correction.

<u>Conclusions</u>

Our study demonstrates that frameshifts can be widespread, with complex patterns of gains and losses within birds and turtles and can be retained for millions of years if they are embedded in a conserved sequence theme.

Keywords

Programmed frameshift, sequence analysis, ancestral state reconstruction, mitochondrion, ND3

Background

Comparative analysis of molecular sequences across the diversity of life allows to discover which molecular mechanisms have been conserved and which have been modified throughout evolution. Insertions or deletions in protein coding genes are usually selected against because they result in frameshifts that disrupt the amino acid coding frame and result in dysfunctional proteins [1]. Albeit rare, examples of corrective frameshifting exist, in which ribosomes regain the proper reading frame [2]. Programmed translational frameshifts have been characterized in viruses, retrotransposons, bacteria, yeast and in mammalian antizymes (reviewed in [3,4]). Additionally, corrective frameshifting was proposed to be acting in the mitochondria of a range of animals (ants [5], glass sponges [6], oysters [7], birds [8,9] and turtles [9,10]), where +1 frameshift insertions have been reported in up to six different mitochondrially-encoded genes [11] with no clear functional consequences. Alternatively, other mechanisms than corrective frameshifting could explain the absence of functional consequences of the frameshift insertion, such as non-canonical translation of tetra or penta codons, which might be an ancient translation mechanism [12], or RNA editing [13].

Three common sequence features that surround frameshift insertions were identified in the better known examples of translational frameshifts (*Ty1* and *Ty3* genes in yeast, antizyme gene in mammals, *prfB* in *E. coli*); a tRNA that enables the ribosome to “slip” on the ribosome P-site, a rarely used codon in the A-site promoting the stall, and a commonly used codon in the +1 frame [14]. In mitochondrial genes with frameshifts, the mechanism leading to a programmed corrective frameshift is not as well characterized but the DNA sequence structure surrounding the mitochondrial frameshift insertions have the same features, indicating that the mechanism that facilitates the correction could be the same [11]. Three models have been proposed to enable translational frameshifting [13]. The “pause-and-slip” model proposes that a pause is

induced at the A-site of the ribosome and that the P-site tRNA can pair with the +1 codon, allowing it to slip out of frame [15]. A second model proposes that abnormal tRNA structures enable the frameshift [16]. The “out-of-frame” model proposes that the recruited tRNA skips the additional nucleotide in the A-site [11]. This “out-of-frame” correction of the frameshift is thought to act in glass sponges, where several mitochondrial genes have been described to contain frameshifts and conserved genomic features that suggest that this mechanism might be acting [13].

The “out-of-frame” model may also apply to the frameshift insertion found in the mitochondrial genomes of certain turtles and birds, where a +1 frameshift insertion occurs at position 174 of the NADH dehydrogenase 3 gene (hereafter ND3-174+1)[9]. The nucleotide sequence around the ND3-174+1 insertion is conserved in a way that would facilitate “out-of-frame” pairing [13]. The insertion is usually found in a codon CUN, with N being the insertion, which produces a wobble pairing with tRNA-leucine when entering the P-site of the ribosome [11]. The codon downstream of the insertion is usually AGU, a rarely used codon for serine, which enters the A-site [11]. The CUN in the P-site and the AGU in the A-site are thought to initiate the frameshift correction by causing a stall in decoding [11]. The AGU codon further forms the beginning of a 15 base pair long stem-loop RNA secondary structure [9], which may enhance the stall [11]. After the stall has been initiated, there are two possibilities. One is to keep the shifted reading frame, which would lead to an early termination of the protein (in birds ending after 207 bp instead of the usual 354 bp [9]), or to produce a +1 frameshift, leaving out A-175 and thus recovering the regular reading frame of the protein.

ND3-174+1 was the first mitochondrial +1 frameshift that was described in vertebrates, when it was initially found in ostrich (*Struthio camelus*) [8]. An extended investigation found that the ND3-174+1 insertion was also present in a species of turtle and in many other bird species

(46/61 bird species) [9]. Within turtle mitogenomes, the insertion was widespread (27/31 turtle species), likely present in their ancestor but lost two to three times within turtles, while being absent in crocodiles (2 species) and snakes (1 species) [11]. From this pattern of absence and presence, it was suggested that the insertion could have been present in a common ancestor of turtles and birds (i.e. Archelosauria: birds+crocodiles, and turtles [17]) and subsequently lost in crocodiles, and lost again in specific lineages of turtles and birds [11]. The insertion was not observed in any other vertebrate lineage [9]. The relatively small number of species investigated in each vertebrate group limited the resolution of the evolutionary history of the insertion. Denser sampling promises to provide additional insight into the distribution of the frameshift insertion in different lineages, and to determine not only the common features of the sequence theme that allow the insertion to remain in the genome, but also find potential deviations from a conserved sequence theme.

Here, we build on the abundance of mitochondrial sequence data publicly available for vertebrates to study the evolution of the frameshift insertion in the mitochondrial protein coding gene ND3. We compiled ND3 sequences representing 10,308 vertebrate taxa and reconstructed ancestral states and transformations of ND3-174+1 in different groups of Diapsida. We further investigated sequence conservation and codon usage patterns around the insertion site to identify potential common sequence motifs associated with the absence or presence of the frameshift insertion. This large dataset provides improved resolution to understand the evolution of this frameshift insertion and highlights that common sequence patterns appear to be required for maintaining the programmed translational frameshift.

Data description

In this work we have compiled a total of 87,708 ND3 sequences from 10,397 unique vertebrate taxa (GigaDB - to be uploaded). Even though our focus was to represent ND3 sequences for

vertebrate species, the taxonomy used on NCBI is not always representing formally named species. The 'Organism' field in NCBI mostly contains traditional Linnaean names for species or subspecies, but in some cases the field contains unnamed taxa such as undescribed species, cryptic species or uncertain species identification (designated with sp., cf. or a placeholder name). Although some of the unique sequence identifiers may not present distinct species, we include these taxa here in addition to the formally named species to include a diversity of patterns in ND3. This decision also allowed us to match each unique sequence identifier to a leaf in the Open Tree of Life tree (see Methods), which also includes NCBI's taxonomy. Sequences were extracted from fragments of ND3 sequences, larger mitochondrial fragments or full mitochondrial genomes downloaded from NCBI's GenBank and RefSeq databases. We reduced the dataset to one representative sequence for each unique entry in the Organism field for the following analyses. If multiple sequences for the same taxon existed, we confirmed that all sequences agreed in the presence or absence of the insertion at position 174. Among intraspecific records, we gave sequences from RefSeq preference over GenBank sequences. Among multiple GenBank records for the same taxon, a random sequence was chosen. This resulted in a dataset of 10,397 vertebrate taxa, which was further filtered to exclude ambiguously aligned sequences to a total of 10,308 taxa for further analysis (GigaDB - to be updated).

Analysis

Patterns of presence and absence of ND3-174+1 in vertebrates

Out of the 10,308 sequences for ND3, a normal reading frame of ND3 without an insertion at position 174, was found in all included sequences of jawless fishes (Cyclostomata, N=30 taxa included), cartilaginous fishes (Chrondrichthyes, N=208), bony fishes (Actinopterygii, N=3,511), coelacanths (Actinistia, N=2), amphibians (Amphibia, N=627), mammals (Mammalia, N=1,683), snakes, lizards, worm lizards and tuatara (Lepidosauria, 305) and crocodiles (Crocodylia,

N=22). A single nucleotide insertion in position 174 was observed in some lineages of turtles (Testudines, N=140; 98 with insertion) and some lineages of birds (Aves, N=3,775; 827 with insertion).

Because the insertion was only found in birds and turtles, we focused further analyses on Diapsida (gene alignment in Additional_file_1 and ND3-174 status in Additional_file_2), for which we obtained an available consensus phylogenetic tree from the Open Tree of Life [18] for 3,464 taxa (Additional_file_3), including Archelosauria (birds (N=3,063), crocodiles (N=22), turtles (N=126)), and their sister group Lepidosauria (N=253) (Figure 1A) (named tips in Additional_file_5). The insertion was absent in 2,654 diapsid taxa, while ND3-174+1 was present in 811 bird and turtle taxa. The inserted nucleotide was cytosine (C) in 749 taxa, thymine (T) in 53, guanine (G) in 7 and adenine (A) in 2 taxa.

We used ancestral state reconstruction using maximum parsimony (MP) and maximum likelihood (ML) with an equal rates model to infer the likelihoods of the presence (any nucleotide) or absence (gap in the alignment) of the insertion at each node in the tree. Branch lengths were set to equal lengths because they are not present in the Open Tree of Life tree. Absence of the ND3-174+1 insertion was inferred in both MP and ML frameworks as the likely ancestral state for the common ancestor of Diapsida, the common ancestor of lizards and snakes (Lepidosauria), the common ancestor of birds and crocodiles (Archosauria), and the common ancestor of Archosauria and turtles (Archelosauria) with high probability (likelihood of absence > 0.99, Figure 1A). The common ancestor of birds was inferred to have contained the insertion (likelihood of presence 0.98). The common ancestor of turtles was reconstructed without the insertion (likelihood of absence 0.97).

In order to quantify the number of state changes between absence and presence of the insertion, we counted the number of transitions across nodes. We chose a conservative approach for inferring a transition, which required that both parent and child nodes had a minimal likelihood > 0.90 for a different state. Nodes with a likelihood < 0.90 in the ML reconstruction also had multiple parsimonious solutions in the MP framework. The MP and ML models therefore inferred the same number of gains and losses. Within turtles and birds, a total of 33 gains and 47 losses of the ND3-174+1 insertion were inferred.

In order to identify the specific nucleotides involved in the insertion and their transitions, we reconstructed ML ancestral states using the five possible states (gap, A, T, C, G) and counted state changes as above. The five state model was able to successfully resolve many nodes that were not resolved in the two state model, which resulted in higher inferred gains and losses of the insertion under the model with five states (38 gains and 52 losses). The gain of the insertion was most frequently a C (34 gains), followed by T (4 gains). Loss of the insertion happened most frequently from C (50 losses), followed by T (2 losses). When the insertion was present, most transitions were from C to T (27 transitions), while other state changes were less frequent (2 transversions from C to G, 1 transversion from C to A, 1 transversion from T to A, 1 transition from T to C).

Complex patterns of gain and loss within turtles and birds

Within turtles, 87 of 126 examined sequences had the insertion and they were dispersed across the phylogeny (Figure 1A). Using the two state models, we inferred four losses within turtles, but no gains were unambiguously estimated because the nodes did not have > 0.90 likelihoods and were therefore not unambiguous in either having the insertion or not having it. The model using five states inferred a complex evolution of the insertion was complex with three inferred gains (likelihood > 0.90), of which two were insertions of C and one was an insertion of T (Figure 1B).

We inferred five losses of the insertion, three losses from an ancestral C state and two losses from an ancestral T state (Figure 1B). Transitions from C to T were most common (7 transitions), compared to other transitions (1 T to C) and transversions (1 T to A). An insertion of G was observed in turtles in a clade of three species (*Malaclemys terrapin, Trachemys scripta, Chrysemys picta* in Emysternia), but the ancestral state could not be inferred unambiguously.

Within birds, 724 of 3,063 examined sequences had the insertion. The common ancestor of birds was inferred to have had an insertion of C (likelihood 0.99). The two state models predicted 32 gains and 49 losses, while the five state model inferred 34 gains and 47 losses (likelihood > 0.90). Gains were mostly of C (31 gains) or of T (3 gains). As in turtles, transitions from C to T (20 transitions) were most common, while other state changes were less frequent (2 transversions from C to G, 1 transversion from C to A). Different orders of birds had different prevalence of the ND3-174+1 insertion or its absence (Figure 1C). The only two orders consistently without the frameshift insertion were the speciose perching birds (Passeriformes, N=2,096 included) and the tropicbirds (Phaethontiformes, N=2) and their ancestors were reconstructed without the insertion (likelihood 0.99). The insertion was present in all examined sequences of 12 orders (Pterocliformes, Columbiformes, Mesitornithiformes, Musophagiformes, Otidiformes, Opisthocomiformes, Eurypygiformes, Suliformes, Cariamiformes, Falconiformes, Coliiformes, Trogoniformes). The remaining bird orders had both lineages with and without the insertion.

Strong sequence conservation surrounding the insertion

In order to identify a potential shared sequence pattern around the ND3-174+1 insertion, we compared the nucleotide diversity between diapsid sequences with and without the insertion. We calculated information content (R) based on Shannon entropy for each base pair, which measures conservation of a sequence position and has a maximum value of 2 bits if the position

is fully conserved. In taxa without the insertion (Lepidosauria, crocodiles, certain turtles and certain birds), the region around position 174 (from 163 to 180 bp) of ND3 had a similar conservation level as the remainder of the gene (Figure 2A). In contrast, all turtle and bird species with the insertion had noticeably more conserved base pairs (higher R) around position 174 than in other regions of ND3 (Figure 2A). The distribution of R around the insertion from position 163 to 180 was 20% lower in species without the insertion than with the insertion (non-parametric Wilcoxon test, p-value $<10^{-3}$). Species with the insertion showed a mean information content a 20% lower than species without the insertion (Figure 2A). Specifically, when the insertion was present, nucleotides upstream of the insertion (position 163 to 174) were highly conserved but some variability on the third codon positions (Figure 2B). The sequence downstream of the insertion (position 175 to 180) was completely conserved with a maximum information content (R=2 bits, Figure 2B). When the insertion was absent, sequence conservation was lower (R<2 bits), particularly on the third codon positions downstream of 174 (Figure 2C).

We also analyzed the codon conservation in sequences containing the insertion (a combined set of turtles and birds with the insertion). The codon containing the insertion was a leucine codon (CTN) in all examined bird and turtle species, with CTC being most prevalent and CTT, CTG and CTA represented at lower frequencies (Figure 2D). Notably, the green wood hoopoe (*Phoeniculus purpureus*, Bucerotiformes) deviated from this pattern with an ATC encoding for isoleucine. Both leucine and isoleucine are nonpolar amino acids. For the codons following the insertion, we considered both the 0 reading frame, which is the shifted reading frame if the frameshift insertion was retained, and the +1 corrected reading frame, which is the frame after the A at position 175 is left out [11]. In the 0 reading frame, the first two codons downstream of the insertion showed almost complete conservation to AGT (encoding serine, a polar amino acid) in the first codon following the insertion (position 175-177) and AGC (encoding serine, a

polar amino acid) in the second codon following the insertion (position 178-180) (Figure 2D). The only exception to this high codon conservation following the insertion was Baillon's crake (*Porzana pusilla*, Gruiformes) with a CGT codon (encoding arginine, a basic amino acid) in the first following codon. In the +1 corrected reading frame leaving out A-175, the codon following the insertion (position 176-178) was a GTA codon (encoding valine, a non-polar amino acid) in all sequences (Figure 2D). The second codon following the insertion (position 179-181) was also conserved in coding for alanine (a non-polar amino acid), albeit with all four synonymous codons present (Figure 2D).

No major tRNA changes in taxa presenting the insertion

Finally, we investigated whether lineages containing the ND3 insertion showed differences in tRNA secondary structure, which could, potentially, enhance programmed frameshifting. We aligned a set of tRNAs of leucine (CUN), serine (UCN) and valine, which are the tRNAs decoding the codon where the insertion occurs (leucine), and the two tRNAs that compete for being decoded downstream of the insertion (serine and valine) (Figure 2D). We compared the predicted consensus secondary structure for each tRNA from four alignments, turtles and bird with and without ND3-174 (Additional_file_6). We did not observe a consistent difference in the secondary structure between bird and turtles with insertion.

Additional potential frameshifts in five turtle species

The translational machinery of birds and turtles seem to enable programmed frameshifting in order to correct single nucleotide insertions in coding regions. Using a subset of Diapsida sequences with full mitochondrial genomes, we checked for other frameshifts in ND3 other than ND3-174. We did not find evidence for additional frameshift insertions in the ND3 sequences of birds (N=703) nor in other Diapsida groups (N=341), except for turtles. Out of 106 investigated mitochondrial genomes of turtles, we identified five putative frameshifts upstream of position

174, namely *Cuora aurocapitata* (A inserted at alignment position 121; NC_009509.1)*, Cuora pani* (T deleted at alignment position 137 or 136; NC_014401.1)*, Cyclemys oldhami* (possible deletion at alignment around position 116; NC_023220), *Pelomedusa subrufa* (G insertion at position 149; NC_001947.1) and *Pelusios castaneus* (G insertion at position 149; NC_026049.1). The two *Cuora* turtles had frameshifts in different positions. *Pelomedusa subrufa* and *Pelusios castaneus* (both Pelomedusidae) had frameshifts at the same location.

For *Pelusios castaneus*, we were able to verify that the frameshift was not due to sequencing errors by mapping high-throughput genomic sequencing reads (SRR9091361) and transcriptomic reads (SRR629649) to the mitochondrial genome (NC_026049.1). The insertion site was verified both in DNA and RNA short reads (Additional_file_7). We however could not check for the frameshift insertions in the other four turtle species due to the lack of additional genomic or transcriptomic data.

Discussion

In this work we have inferred multiple origins of an insertion in position 174 of the mitochondrially-encoded NADH dehydrogenase 3 complex gene (ND3-174+1) based on a large collection of publicly available sequences for 10,308 vertebrates. This study significantly expands the sampling of previous studies (61 species in [9], 34 species in [11]) to provide a broader picture across vertebrates on one hand and more fine-scale resolution of sequence conservation on the other hand. We confirm that the insertion is present exclusively in turtles and birds [9,11] but the improved sampling shows that both the insertion was more frequently gained and lost than previously thought. Different from previous interpretations, which predicted the presence of this insertion in the common ancestor of turtles and birds (Archelosauria), ML and MP ancestral state reconstruction suggested an independent evolution of this insertion in birds and turtles. Within birds, the insertion was reconstructed as present in the most recent

common ancestor of modern birds, which lived about 70-111 million years ago (depending on the phylogeny, [19,20]). Once obtained, the insertion was retained in many lineages but lost in the common ancestor of Passeriformes 39-49 million years ago [19–21] and not regained since. Additionally, within the other bird lineages, it appeared more likely to lose the insertion than to gain it (60% of changes are insertion losses, while 40% are insertion gains across all tested models). The most recent common ancestor of turtles was inferred to not have had an insertion in ND3, as opposed to previous ideas [9,11]. Our data included 126 species of turtles, 93 more than in the last study on turtle mitochondrial genomes [11], which produced an alternative interpretation of the gain and loss patterns. Within turtles, the insertion has been independently gained one to three times based on our reconstructions. Within clades that have insertion, some lineages show mutations to other nucleotides. The higher prevalence of C to T transitions both in turtles and birds (27/32, 84% of mutations) could be a consequence of cytosine methylation, which has been described to be present also in mitochondrial genomes [22].

The quality of the ND3 sequences and the observed absence or presence of the insertion at position 174 on these sequences is of crucial importance for our inferences. Most of the ND3 sequences used here originate from Sanger sequenced ND3 genes, whose chromatograms may have been hand-curated for sequencing errors. While insertions at position 174 are likely to be genuine, as they would have been flagged as problematic during submission to NCBI's Genbank and would require a special annotation to address the frameshift insertion (often to the Mindell et al. study [9]), their absence may be overrepresented. A frameshift insertion in ND3 may have been curated out of the sequence because such insertions in the protein coding sequence are extremely rare and may have therefore been considered a sequencing error. It is therefore possible that the number of loss events in turtles and birds could be an overestimation. Where losses were observed in multiple members of a clade, the most extreme case being the absence in all 2,096 included Passeriformes, the absence of the insertion is

likely to be genuine. To estimate the prevalence of this problem, we compared ND3 annotations for 101 bird species that both have sequenced ND3 sequences on NCBI (mostly Sanger sequenced) and also high-throughput sequenced mitochondrial genomes from the B10K project, for which we have created the annotations ourselves and can therefore exclude manual modification. Reassuringly, we found that there were no cases in which the B10K dataset did not contain the insertion while the GenBank sequence contained it. This assessment admittedly spans only a small fraction of the taxa investigated here but lends support that at least in birds annotation errors may be limited. It is possible that submitters of bird and turtle sequences are more aware of the possibility of a frameshift insertion because the insertions have been reported from these taxa [9,11] than submitters of taxa in which the insertion has not been previously observed, such as Lepidosauria or Mammalia.

It is intriguing that the insertion in exactly the same position of the ND3 gene has independently evolved 37 (33 according to the parsimony model) times, both in turtles and birds. This site-specificity points to an underlying common feature that causes the frameshift to occur in this position. One possibility is that there is an increased probability to produce indels in that specific position. Alternatively, insertions may appear at a normal rate but only get tolerated if they are embedded in a specific sequence motif that allows ribosomes to conduct the frameshift correction. This seems likely given the strong conservation at the nucleotide and at the codon level that evolved convergently in birds and turtles, despite their separate evolution for over 240 million years. Intriguingly, this conservation is not found in birds and turtles without the insertion (Figure 2A) nor in the other diapsids without the insertion, as expected with the degree of divergence of vertebrates. Further, the same sequence and codon conservation observed around ND3-174+1have been similarly described in other mitochondrial frameshifts across

animals [11]. This indicates that the heavily conserved sequence is needed for correcting the insertion and tolerating it in the mitochondrial genome.

Most of the features observed in this study conformed with the out-of-frame frameshift model, as already described [11], characterized by a weak codon tRNA interaction in the P-site, a rare codon downstream, and an alternative codon if the reading frame is restored which achieves a canonical Watson-Crick match with its tRNA [13].

While our study confirmed the previous prediction on the presence of conserved features for sequences surrounding the insertion [11], our extended sampling provides higher resolution of the sequence conservation features that may be involved in the programmed translational frameshift. The codon containing the insertion was a leucine codon (CTN) in all examined bird and turtle species with the exception of one bird (ATC in the green wood hoopoe *Phoeniculus purpureus*). This codon upstream from the translational frameshift position was proposed to produce a wobble pairing initiating a translation stall [11]. We further confirm that a serine codon (AGT), a polar amino acid, was always observed after the insertion [11]. However, our extended sampling shows that this pattern can be more flexible in at least one bird that has an arginine codon (CGT), a basic amino acid (Figure 2D). The consequences of this amino acid change on the programmed translational frameshift are unknown. The AGT codon following the insertion is thought to further promote the stall in the translation, which in turn facilitates the frameshift because it is a rarely used codon for serine [11]. If the observed variable codon, CGT, was also a rarely used codon for arginine, the hypothesis that rare codons promote the translation stall would still hold. Indeed, in human mitochondria CGT is the third least used codon out of four possible arginine codons [23]. If the codon usage was conserved across vertebrates, these rare codons may indeed promote the stall in addition.

Regarding the insertion itself, we have observed all four nucleotides to be present in different frequencies. According to the out-of-frame frameshift model, wobble pairing between a sequence and the tRNA anticodon promotes frameshifting [11]. Consequently, the insertion should rarely occur as an adenosine because a CTA codon produces a perfect match with the tRNA-leucine anticodon [11]. In addition to Reeve's turtle (*Chinemys reevesi*), which has previously been shown to contain an A-insertion [11], we found an independent occurrence of A-insertion in a bird, Baillon's crake (*Porzana pusilla*), which also has a non-synonymous codon right after the insertion as described above. These two species may therefore be an interesting candidates for further investigations on the programmed translational frameshift in the absence of wobble pairing.

In addition to the detailed investigation of position 174, we found four additional frameshifts in ND3 in five turtles. Of these, the African helmeted turtle (*Pelomedusa subrufa*) was already described to contain such a frameshift [11] and the frameshift of the West African mud turtle (*Pelusios castaneus*) in the same position was annotated as a frameshift insertion on NCBI. These two species are Pelomedusidae and both *Pelusios* and *Pelomedusa* contain a number of species [24], which could be sequenced for ND3 to investigate if the insertion is shared across Pelomedusidae and independently obtained in the two species. The other three frameshifts are, to the best of our knowledge potential new frameshifts in the ND3 gene. These findings support previous observations on the ubiquitous presence of such frameshifts in other mitochondrial genes of turtles [11], suggesting a broader tolerance of turtles to these frameshift insertions.

Our study demonstrates that incorporating a large number of sequences can improve resolution in inferred evolutionary patterns and give additional power to investigate sequence conservation. Our analyses suggested an independent origin of the frameshift insertion both in turtles and birds, and complex patterns of gains and losses within each group. The high

sequence conservation surrounding the insertion suggests purifying selection retaining the sequence motifs needed for translational frameshifting. Nonetheless, a few species deviate from the conserved pattern. Additional losses and gains of the insertion and other deviations from the conserved motifs will likely be found once more ND3 sequences become available, within birds and turtles and possibly also in other vertebrates.

Potential implications

The current work advances our understanding of the distribution of the frameshift insertion in the mitochondrial gene ND3 across the vertebrate tree of life and identifies highly conserved sequence features that seem to be associated with its occurrence. This will allow researchers to further study which sequence features allow for the corrective frameshift and to investigate the evolutionary constraints that keep the surrounding sequence heavily conserved. The fact that this insertion has remained in the mitochondrial genome for millions of years in certain birds and turtles opens the door to study the translational machinery in these lineages.

Methods

Dataset preparation

All RefSeq mitochondrial genomes were downloaded from the NCBI FTP site (ftp://ftp.ncbi.nlm.nih.gov/refseq/release/mitochondrion/, downloaded 2019-12-03), resulting in 5,325 mitochondrial genomes with the term 'Vertebrata' contained in the taxonomy, from which we could retrieve the ND3 sequence in 5,320 records. We searched for nucleotide sequences containing the ND3 gene on the GenBank nucleotide database (accessed 2019-12-11) with the query "NADH dehydrogenase subunit 3" AND Vertebrata[Organism] AND mitochondrion[filter]". A total of 92,352 sequence records were downloaded using a custom script. From those, we were able to find 88,880 records of ND3 sequences. We further included mitochondrial data

from the second phase of the Bird 10,000 genome project (B10K), which produced 336 mitochondrial genomes as part of their whole genome sequences (Feng et al., manuscript accepted). Of these mitochondrial genomes, we included 207, which had ND3 sequences assembled and which were added if the species was not already present in the database.

Sequence alignment

One ND3 sequence for each unique taxon (10,397) was aligned using MAFFT FFT-NS-1 (v7.407) for initial alignment [25] (GigaDB - to be uploaded) Next, sequences were pruned from positions not seen in at least 5% of the sequences using pxclsq (v0.1) (GigaDB - to be uploaded) from the phyx suite [26] to exclude insertions only seen in a small proportion of taxa. Note that we also explicitly investigated other frameshifts than ND3-174+1 across ND3 in birds and turtles (section Investigating other possible frameshift insertions), while this alignment was focused on the region around position 174. We filtered records containing two gaps or unknown nucleotides in the two codons adjacent to the insertion in and that after a visual examination seem not to properly align around ND3-174. We performed a second alignment round on the remaining sequences with a slower and more accurate mode of MAFFT (L-INS-i). We repeated filtering positions not in at least 5% of sequences. To remove potential low quality sequences around the insertion We removed sequences that contained a gap or an N in the two codons (six nucleotides) upstream and downstream of the insertion. The final filtered alignment of 10, 307 sequences is given in GigaDB (to be uploaded).

To confirm that all sequences of the same unique taxon had the same pattern at position 174, we used the filtered alignment as a framework for aligning all other intraspecific records, by using the --add function from MAFFT and applying the same same filters as above (GigaDB-to be uploaded).

Phylogenetic distribution of the insertion

The frameshift insertion in position 174 was only observed in certain species of turtles and birds and we therefore restricted analyses to Diapsida, i.e. birds, crocodiles, turtles and Lepidosauria (tuatara, worm lizards, snakes and lizards). This left 4,233 out of 10,397 vertebrate taxa. We recorded the state of position 174 in each sequence, either being a gap in the alignment (i.e. insertion absent), or being a nucleotide (i.e. insertion present as A,T,C,G).

We used the R package rotl (v3.0.10) [27] to obtain a phylogenetic tree for the included species. The package queries the Open Tree of Life (otol) database (https://tree.opentreeoflife.org/opentree/argus/opentree12.3@ott93302), which synthesizes phylogenetic hypotheses from published datasets and adds species that have not been included in phylogenetic analyses based on the taxonomic system [18,28]. While a fully sampled tree for Diapsida would be preferable over a synthetic tree, it agrees in the relationships among the major Diapsida clades with phylogenetic analyses [29–32]. Of the 4,233 Diapsida species with ND3 records, 3,465 could be matched with a terminal on the Open Tree of Life (newick tree in Additional File 2). In order to summarize the distribution of absence or presence of the insertion on a dated bird phylogeny, we used the fossil-calibrated phylogenetic tree from [19].

Ancestral state reconstruction

Maximum likelihood (ML) ancestral states were reconstructed using the R package Castor (v1.5.5) [33] using the function hsp_mk_model. The function first calculates the transition matrix between different states assuming equal-rates for transitioning from one state to another and vice versa in a ML framework. We chose the equal-rates model because it makes the least assumptions about the probabilities of gain, loss and transitions between different states. Given the known states of the tips and the phylogenetic tree, the likelihood of each node in the tree was calculated using the rerooting method [34]. Because the Open Tree of Life synthetic

phylogeny did not include branch lengths, the function assumes equal branch lengths throughout the phylogeny. ML analyses were both done for two states (absence or presence of the insertion) and for the five possible states (absence, A, C, G, T). Maximum parsimony (MP) reconstruction of ancestral states were performed with the function MPR in the ape package [35]. MP analysis was done for two states (absence or presence of the insertion). Prior to analysis, polytomies were arbitrarily resolved using the function multi2di in ape.

Inference of transitions between states

In order to count the number of nodes of the phylogeny where transitions from one state to another likely occurred, we related the likelihood from the ancestral state reconstruction of each descendant node to its parent node using the R package phangorn [36]. In the ML model, we only considered nodes with an ancestral state likelihood > 0.9. If the likelihood was < 0.9, the state was considered as ambiguous. A transition was counted when a descendant node differed from its parent's state with high likelihood. This approach therefore only identifies transitions that are accompanied with strong changes in likelihoods. On the other hand, in the MP model, we counted transitions where both the insertion status parental and child nodes could be resolved unambiguously. We counted the number of transitions between absence and presence of the insertion in both the ML and MP model with two states and the number of transitions between the five states (absence, A, C, G, T) in the ML model.

Sequence conservation and codon usage

Nucleotide frequencies per position across the entire ND3 sequence were obtained separately for diapsids without the insertion and with the insertion. We calculated Shannon entropy as a measure of nucleotide diversity [9]:

$$H_i = -\sum_N freq_N \ x \ log_2(freq_N)$$

where *H* is the Shannon entropy in position i of the DNA sequence, $freq_N$ is the frequency of nucleotide *N* of state {A,T,G,C}. Shannon entropy was transformed into information content per nucleotide position:

$$R_i = log_2(4) - H_i$$

where R is the information content at position i [37]. The information content was compared between the two groups in the region surrounding the insertion (position 163-180) with a non-parametric Wilcoxon test and a significance threshold (α) of $p<0.05$. Weblogo [38] was used to visualize the information content of this region (position 163-181) for both groups, where the relative diversity versus conservation of each nucleotide is reflected as the height of the nucleotide, measured in bits with a maximum value of 2 at complete conservation.

Codon frequencies were calculated for both the shifted reading frame, the 0 reading frame, and for the corrected reading frame, the +1 reading frame. Codons containing unknown nucleotides (N) were removed. Codon frequencies were calculated for the six codons surrounding the insertion (position 163-181). The calculation of codon frequencies in the +1 reading frame excluded the adenosine at position 175(A-175) following the insertion [11].

tRNA secondary structure prediction

We extracted the sequences for three tRNAs involved in translation of codons surrounding the insertion from our compiled list of mitochondrial genomes in birds and turtles present in the ND3 filtered alignment. We extracted the tRNA for leucine, translating the codon upstream of the insertion; serine, translating the codon following the insertion in the shifted reading frame; valine, translating the codon following the insertion in the corrected reading frame. We split the sequences in a group of birds and a group of turtles with and without the insertion. Short sequences were removed if they were between 2 times the standard deviation of the mean nucleotide length of the group. An initial alignment was done using the mlocarna tRNA aligner

(v2.0.0RC8) [39], which simultaneously aligns and folds RNA sequences. Two tRNAs exist for leucine and serine in mitochondrial genomes, of which CUN-leucine and UCN-serine are involved in translating the codons around the ND3-174+1. Because Genbank records do not always distinguish between the two tRNAs, we kept the tRNA copy with the higher pairwise identity to a known representative CUN-leucine and UCN-serine tRNA. Sequences passing the filter were realigned with mlocarna. The predicted consensus secondary structure of the tRNA alignment was visualized using the ViennaRNA web services [40].

Other possible frameshift insertions in ND3

In order to extend the study to other potential insertions in the ND3 gene in Diapsida, we compiled a dataset of 1,050 complete mitochondrial genomes from the Refseq database. We excluded records without annotations or with undetermined nucleotides (N) in the ND3 gene, resulting in 1,044 ND3 gene sequences (Crocodilia N=20, Lepidosauria N=215, Testudines N=106, Aves N=703). As a further check for consistency, we also investigated a second dataset focused on birds belonging to the B10K project including 328 mitochondrial genomes that were *de novo* assembled from shotgun genomic reads [Feng et al. manuscript accepted].

Both ND3 sequence sets were aligned using the vertebrate mitochondrial genetic codon table ("-gc_def 2") in the "alignSequence" module of MACSE (v2.01) [41], which respects reading frames and can tolerate frameshifts (Additional_file_4). MACSE designates frameshifts with the symbol '!' and candidates were visually verified with the alignment viewer Seaview (v5.0.4) [42]. Individual ND3 gene sequences were also annotated using "protein2genome" model of exonerate (v2.4.0) [43] by mapping the *Gallus gallus* ND3 amino acid sequence (NC_040902.1) to each of the diapsid ND3 nucleotide gene sequences. Exonerate designates potential frameshifts with the symbol '#'.

Availability of source code and requirements

Scripts used for data generation and analysis can be found at:

https://github.com/sergioSEa/ND3_174_vertebrates2020

Operating system(s): e.g. Bash scripts should be run in Linux OS/Mac OS. Python and R scripts are platform independent.

Programming language: Bash, R, Python

Other requirements: Python 3 or higher, Mafft v7.4, pxclsq v0.1. Python packages: biopython. R packages: rotl, castor, ape, phytools, ggtree, ggimage, phangorn, ggstance, Biostrings, ggrepel and tidyverse.

License: GNU

Availability of supporting data and materials

The data sets supporting the results of this article are available in the [repository name, to be included once uploaded] repository, [Identifier]."

List of abbreviations

ND3 - NADH dehydrogenase 3 complex gene

A - adenosine

C - cytosine

T - thymine

G - guanine

N - undetermined nucleotide

ML - maximum likelihood

MP - maximum parsimony

NGS - Next generation sequencing

Author's contributions

Sergio Andreu-Sánchez: Formal analysis, writing, visualization. conceptualization; Wanjun Chen: data curation, formal analysis, visualization; Josefin Stiller: Conceptualization, supervision, visualization, writing; Guojie Zhang: Conceptualization; supervision; resources; writing

Acknowledgements

This project was supported by Carlsberg Foundation (CF16-0663). It was partially supported by the Strategic Priority Research Program of the Chinese Academy of Sciences (XDB31020000). GZ is also supported by Villum Foundation (No. 25900).

Additional material

Additional_file_1: **Diapsida MAFTT alignment.** Diapsida MAFFT alignment after removal of positions not seen in at least 5% of taxa.

Additional_file_2: **Diapsida Table**. Table of Diapsida taxa and the corresponding status at ND3-174 used for ancestral state reconstruction extracted from Addtional_file_1.

Additional_file_3 **Diapsida tree.** Open tree of life matched to the Diapsida taxa included in the study.

Additional_file_3: **Diapsida MACSE alignment**. Alignment of 1,044 Refseq Diapsida sequences used for identification of other frameshifts.

Additional_file_4: **Labelled diapsida tree.** Diapsida tree as presented in Figure 1A, but including taxa names. PDF file.

Additional_file_5: **Consensus tRNA structure**. Consensus predicted tRNA structure of birds and turtles with and without gap from Valine, Leucine and Serine. Nucleotides presented do not represent the real consensus sequence.

Additional_file_6: **NGS read mapping to *Pelusios castaneus***. Genomic (SRR9091461) and transcriptomic (SRR629649) NGS reads were mapped to *Pelusios castaneus* mitochondrial genome (NC_026049.1) to check whether the predicted frameshift site was present.

References

1. Tse H, Cai JJ, Tsoi H-W, Lam EP, Yuen K-Y. Natural selection retains overrepresented out-of-frame stop codons against frameshift peptides in prokaryotes. BMC Genomics. 2010;11:491.

2. Atkins JF, Loughran G, Bhatt PR, Firth AE, Baranov PV. Ribosomal frameshifting and transcriptional slippage: From genetic steganography and cryptography to adventitious use. Nucleic Acids Res. 2016;44:7007–78.

3. Farabaugh PJ. Programmed translational frameshifting. Microbiol Rev. 1996;60:103–34.

4. Dinman JD. Mechanisms and implications of programmed translational frameshifting. Wiley Interdiscip Rev RNA. 2012;3:661–73.

5. Beckenbach AT, Robson SKA, Crozier RH. Single Nucleotide +1 Frameshifts in an Apparently FunctionalMitochondrial Cytochrome b Gene in Ants of the Genus Polyrhachis. J Mol Evol. 2005;60:141–52.

6. Rosengarten RD, Sperling EA, Moreno MA, Leys SP, Dellaporta SL. The mitochondrial genome of the hexactinellid sponge Aphrocallistes vastus: Evidence for programmed translational frameshifting [Internet]. BMC Genomics. 2008. p. 33. Available from: http://dx.doi.org/10.1186/1471-2164-9-33

7. Milbury CA, Gaffney PM. Complete mitochondrial DNA sequence of the eastern oyster Crassostrea virginica. Mar Biotechnol . 2005;7:697–712.

8. Härlid A, Janke A, Arnason U. The mtDNA sequence of the ostrich and the divergence between paleognathous and neognathous birds. Mol Biol Evol. 1997;14:754–61.

9. Mindell DP, Sorenson MD, Dimcheff DE. An extra nucleotide is not translated in mitochondrial ND3 of some birds and turtles. Mol Biol Evol. 1998;15:1568–71.

10. Parham JF, Macey JR, Papenfuss TJ, Feldman CR, Türkozan O, Polymeni R, et al. The phylogeny of Mediterranean tortoises and their close relatives based on complete mitochondrial genome sequences from museum specimens. Mol Phylogenet Evol. 2006;38:50–64.

11. Russell RD, Beckenbach AT. Recoding of translation in turtle mitochondrial genomes: programmed frameshift mutations and evidence of a modified genetic code. J Mol Evol. 2008;67:682–95.

12. Seligmann H, Warthi G. Chimeric Translation for Mitochondrial Peptides: Regular and Expanded Codons. Comput Struct Biotechnol J. 2019;17:1195–202.

13. Haen KM, Pett W, Lavrov DV. Eight new mtDNA sequences of glass sponges reveal an extensive usage of+ 1 frameshifting in mitochondrial translation. Gene. Elsevier; 2014;535:336–44.

14. Baranov PV, Gesteland RF, Atkins JF. Recoding: translational bifurcations in gene expression. Gene. 2002;286:187–201.

15. Huang Y, Koonin EV, Lipman DJ, Przytycka TM. Selection for minimization of translational frameshifting errors as a factor in the evolution of codon usage. Nucleic Acids Res. 2009;37:6799–810.

16. Sroga GE, Nemoto F, Kuchino Y, Björk G. Insertion (sufB) in the anticodon loop or base substitution (sufC) in the anticodon stem of tRNA Pro2 from Salmonella typhimurium induces suppression of frameshift mutations. Nucleic Acids Res. Oxford University Press; 1992;20:3463–9.

17. Crawford NG, Parham JF, Sellas AB, Faircloth BC, Glenn TC, Papenfuss TJ, et al. A phylogenomic analysis of turtles. Mol Phylogenet Evol. 2015;83:250–7.

18. Redelings BD, Holder MT. A supertree pipeline for summarizing phylogenetic and taxonomic information for millions of species. PeerJ. 2017;5:e3058.

19. Jarvis ED, Mirarab S, Aberer AJ, Li B, Houde P, Li C, et al. Whole-genome analyses resolve early branches in the tree of life of modern birds. Science. 2014;346:1320–31.

20. Prum RO, Berv JS, Dornburg A, Field DJ, Townsend JP, Lemmon EM, et al. A comprehensive phylogeny of birds (Aves) using targeted next-generation DNA sequencing. Nature. 2015;526:569–73.

21. Oliveros CH, Field DJ, Ksepka DT, Barker FK, Aleixo A, Andersen MJ, et al. Earth history and the passerine superradiation. Proc Natl Acad Sci U S A. 2019;116:7916–25.

22. Sirard M-A. Distribution and dynamics of mitochondrial DNA methylation in oocytes, embryos and granulosa cells. Sci Rep. 2019;9:11937.

23. Jia W, Higgs PG. Codon usage in mitochondrial genomes: distinguishing context-dependent mutation from translational selection. Mol Biol Evol. 2008;25:339–51.

24. Fritz U, Branch WR, Hofmeyr MD, Maran J, Prokop H, Schleicher A, et al. Molecular phylogeny of African hinged and helmeted terrapins (Testudines: Pelomedusidae: Pelusios and Pelomedusa). Zool Scr. 2011;40:115–25.

25. Katoh K, Standley DM. MAFFT multiple sequence alignment software version 7: improvements in performance and usability. Mol Biol Evol. 2013;30:772–80.

26. Brown JW, Walker JF, Smith SA. Phyx: phylogenetic tools for unix. Bioinformatics.

2017;33:1886–8.

27. Michonneau F, Brown JW, Winter DJ. rotl : an R package to interact with the Open Tree of Life data. Fitzjohn R, editor. Methods Ecol Evol. 2016;7:1476–81.

28. Rees JA, Cranston K. Automated assembly of a reference taxonomy for phylogenetic data synthesis. Biodivers Data J. 2017;e12581.

29. Green RE, Braun EL, Armstrong J, Earl D, Nguyen N, Hickey G, et al. Three crocodilian genomes reveal ancestral patterns of evolution among archosaurs. Science. 2014;346:1254449.

30. Irisarri I, Baurain D, Brinkmann H, Delsuc F, Sire J-Y, Kupfer A, et al. Phylotranscriptomic consolidation of the jawed vertebrate timetree. Nat Ecol Evol. 2017;1:1370–8.

31. Chiari Y, Cahais V, Galtier N, Delsuc F. Phylogenomic analyses support the position of turtles as the sister group of birds and crocodiles (Archosauria). BMC Biol. 2012;10:65.

32. Crawford NG, Faircloth BC, McCormack JE, Brumfield RT, Winker K, Glenn TC. More than 1000 ultraconserved elements provide evidence that turtles are the sister group of archosaurs. Biol Lett. 2012;8:783–6.

33. Louca S, Doebeli M. Efficient comparative phylogenetics on large trees. Bioinformatics. 2018;34:1053–5.

34. Yang Z, Kumar S, Nei M. A new method of inference of ancestral nucleotide and amino acid sequences. Genetics. 1995;141:1641–50.

35. Paradis E, Claude J, Strimmer K. APE: Analyses of Phylogenetics and Evolution in R language. Bioinformatics. 2004;20:289–90.

36. Schliep KP. phangorn: phylogenetic analysis in R. Bioinformatics. 2011;27:592–3.

37. Schneider TD, Stephens RM. Sequence logos: a new way to display consensus sequences. Nucleic Acids Res. 1990;18:6097–100.

38. Crooks GE, Hon G, Chandonia J-M, Brenner SE. WebLogo: a sequence logo generator. Genome Res. 2004;14:1188–90.

39. Smith C, Heyne S, Richter AS, Will S, Backofen R. Freiburg RNA Tools: a web server integrating INTARNA, EXPARNA and LOCARNA. Nucleic Acids Res. 2010;38:W373–7.

40. Kerpedjiev P, Hammer S, Hofacker IL. Forna (force-directed RNA): Simple and effective online RNA secondary structure diagrams. Bioinformatics. 2015;31:3377–9.

41. Ranwez V, Harispe S, Delsuc F, Douzery EJP. MACSE: Multiple Alignment of Coding SEquences accounting for frameshifts and stop codons. PLoS One. 2011;6:e22594.

42. Gouy M, Guindon S, Gascuel O. SeaView version 4: A multiplatform graphical user interface for sequence alignment and phylogenetic tree building. Mol Biol Evol. 2010;27:221–4.

43. Slater GSC, Birney E. Automated generation of heuristics for biological sequence comparison. BMC Bioinformatics. 2005;6:31.

Figures and legends

Figure 1. Phylogenetic distribution of the insertion in position 174 of the mitochondrial ND3 gene (ND3-174+1). (a) Synthetic phylogeny of 3,464 species of Diapsida with terminals colored according to the absence (orange) or presence (blue) of the insertion. (b) Ancestral state reconstruction for turtles with states of species indicated at the tips and pie charts showing the likelihoods of different states on nodes of the phylogeny. (c) Frequency of absence (orange) or presence (blue) of the insertion in each bird order. Numbers represent the number of species that were included for each order.

Figure 2. **Nucleotide and codon usage variability in ND3 of Diapsida.** (a) Information content R (cubed for visualization) across the ND3 sequence in different diapsid groups. The vertical red line marks the insertion at position 174. The red shading highlights an area of high conservation (high information content) only seen in birds and turtles that have the insertion. (b-c) Sequence conservation as a sequence logo from position 163 to 180 showing variability among species (b) with the insertion and (c) without the insertion. Note that the frameshift correction is thought to occur at the following base, by skipping the nucleotide A at position 175. (d) Circle packing showing the frequency of codon usage for each codon in species that contain the insertion. The two options of the shifted and corrected reading frame following the insertion at position 174 are shown. Circle diameters indicate prevalence of a specific codon, which are grouped into larger circles if codons are synonymous. Circle color indicates amino acid class.

Click here to access/download
Supplementary Material
Additional_file_1.tsv

Click here to access/download
Supplementary Material
Additional_file_2.tsv

Click here to access/download
Supplementary Material
Additional_file_4.tsv

Click here to access/download
Supplementary Material
Additional_file_7.PNG

Click here to access/download
Supplementary Material
Additional_file_3.txt

Click here to access/download
Supplementary Material
Additional_file_5.pdf

Click here to access/download
Supplementary Material
Additional_file_6.pdf

Figure addressing Reviewer 2 comment

Click here to access/download
Supplementary Material
renamed_222e0.pdf

Alignment addressing Reviewer 2 comments

Click here to access/download
Supplementary Material
Reviewer_2_alignment.txt
