# Supplementary material for: Multiple origins of a frameshift insertion in a mitochondrial gene in birds and turtles
Source: Gigascience. 2021 Jan 19;10(1):giaa161. doi: 10.1093/gigascience/giaa161 (PMC7814300; doi:10.1093/gigascience/giaa161)
Supplement: giaa161_GIGA-D-20-00122_Revision_2 [file giaa161_giga-d-20-00122_revision_2.pdf]

GigaScience

Multiple origins of a frameshift insertion in a mitochondrial gene in birds and turtles

--Manuscript Draft--

Manuscript Number:	GIGA-D-20-00122R2
Full Title:	Multiple origins of a frameshift insertion in a mitochondrial gene in birds and turtles
Article Type:	Research
Funding Information:	Carlsbergfondet (DK) (CF16-0663) — Dr Guojie Zhang<br>Strategic Priority Research Program of the Chinese Academy of Sciences (XDB31020000) — Dr Guojie Zhang<br>Villum Fonden (25900) — Dr Guojie Zhang

Abstract:

Abstract

Background

During evolutionary history, molecular mechanisms have emerged to cope with deleterious mutations. Frameshift insertions in protein coding sequences are extremely rare because they disrupt the reading frame. There are a few known examples of their correction through translational frameshifting, a process which enables ribosomes to skip nucleotides during translation to regain proper reading frame. Corrective frameshifting has been proposed to act on the single base pair insertion at position 174 of the mitochondrial NADH dehydrogenase subunit 3 gene (ND3) that has been observed in several turtles and birds. However, the relatively sparse taxonomic representation has hampered our understanding on the evolution of this insertion in vertebrates.

Results

Here, we analyzed 87,707 ND3 sequences from 10,309 vertebrate taxa to reveal the evolutionary history of this insertion and its common genomic characteristics. We confirmed that the insertion only appears in turtles and birds and reconstructed that it evolved independently in both groups with complex patterns of gains and losses. The insertion was observed in almost all bird orders but was absent in all members of the diverse Passeriformes. We found strong conservation in the nucleotides surrounding the insertion in both turtles and birds, which implies that the insertion enforces structural constraints that could be involved in its correction.

Conclusions

Our study demonstrates that frameshifts can be widespread and can be retained for millions of years if they are embedded in a conserved sequence theme.

Corresponding Author:	Sergio Andreu-Sánchez<br>Universitair Medisch Centrum Groningen<br>Groningen, Groningen NETHERLANDS
Corresponding Author Secondary Information:	
Corresponding Author's Institution:	Universitair Medisch Centrum Groningen
Corresponding Author's Secondary Institution:	
First Author:	Sergio Andreu-Sánchez
First Author Secondary Information:	
Order of Authors:	Sergio Andreu-Sánchez
	Josefin Stiller
	Wanjun Chen
	Guojie Zhang
Order of Authors Secondary Information:	

Powered by Editorial Manager® and ProduXion Manager® from Aries Systems Corporation

Response to Reviewers:	We are glad to comply with the last suggestion of the reviewer and to edit the header of the section he refers to.<br>At the same time, we have now included information about the GigaDB repository.
Additional Information:	
Question	Response
Are you submitting this manuscript to a special series or article collection?	No
**Experimental design and statistics**<br><br>Full details of the experimental design and statistical methods used should be given in the Methods section, as detailed in our Minimum Standards Reporting Checklist. Information essential to interpreting the data presented should be made available in the figure legends.<br><br>Have you included all the information requested in your manuscript?	Yes
**Resources**<br><br>A description of all resources used, including antibodies, cell lines, animals and software tools, with enough information to allow them to be uniquely identified, should be included in the Methods section. Authors are strongly encouraged to cite Research Resource Identifiers (RRIDs) for antibodies, model organisms and tools, where possible.<br><br>Have you included the information requested as detailed in our Minimum Standards Reporting Checklist?	Yes
**Availability of data and materials**<br><br>All datasets and code on which the conclusions of the paper rely must be either included in your submission or deposited in publicly available repositories (where available and ethically appropriate), referencing such data using a unique identifier in the references and in	No

the “Availability of Data and Materials” section of your manuscript.<br><br>Have you have met the above requirement as detailed in our Minimum Standards Reporting Checklist?	
If not, please give reasons for any omissions below.<br><br>as follow-up to "**Availability of data and materials**<br><br>All datasets and code on which the conclusions of the paper rely must be either included in your submission or deposited in publicly available repositories (where available and ethically appropriate), referencing such data using a unique identifier in the references and in the “Availability of Data and Materials” section of your manuscript.<br><br>Have you have met the above requirement as detailed in our Minimum Standards Reporting Checklist?<br><br>"	All data will be deposited in GigaDB upon confirmation from the editor.

Powered by Editorial Manager® and ProduXion Manager® from Aries Systems Corporation

Manuscript Click here to access/download;Manuscript;Andreu-Sanchez_etal_forproduction.docx

Research

Multiple origins of a frameshift insertion in a mitochondrial gene in birds and turtles

Sergio Andreu-Sánchez[1,*]https://orcid.org/0000-0002-3503-9971, Wanjun Chen[2], Josefin Stiller[1]https://orcid.org/0000-0001-6009-9581, Guojie Zhang[1,2,3,4]https://orcid.org/0000-0001-6860-1521

[1] Villum Centre for Biodiversity Genomics, Section for Ecology and Evolution, Department of Biology, University of Copenhagen, Denmark
[2] BGI-Shenzhen, Beishan Industrial Zone, Shenzhen 518083, China
[3] State Key Laboratory of Genetic Resources and Evolution, Kunming Institute of Zoology, Chinese Academy of Sciences, Kunming, 650223, China
[4] Center for Excellence in Animal Evolution and Genetics, Chinese Academy of Sciences, 32 Jiaochang Donglu, Kunming 650223, China

*Current address: University of Groningen, University Medical Center Groningen, Department of Pediatrics, 9700 RB Groningen, Netherlands.

Corresponding author: guojie.zhang@bio.ku.dk

Abstract

Background

During evolutionary history, molecular mechanisms have emerged to cope with deleterious mutations. Frameshift insertions in protein coding sequences are extremely rare because they disrupt the reading frame. There are a few known examples of their correction through translational frameshifting, a process which enables ribosomes to skip nucleotides during translation to regain proper reading frame. Corrective frameshifting has been proposed to act on the single base pair insertion at position 174 of the mitochondrial NADH dehydrogenase subunit 3 gene (ND3) that has been observed in several turtles and birds. However, the relatively sparse taxonomic representation has hampered our understanding on the evolution of this insertion in vertebrates.

Results

Here, we analyzed 87,707 ND3 sequences from 10,309 vertebrate taxa to reveal the evolutionary history of this insertion and its common genomic characteristics. We confirmed that the insertion only appears in turtles and birds and reconstructed that it evolved independently in both groups with complex patterns of gains and losses. The insertion was observed in almost all bird orders but was absent in all members of the diverse Passeriformes. We found strong conservation in the nucleotides surrounding the insertion in both turtles and birds, which implies that the insertion enforces structural constraints that could be involved in its correction.

Conclusions

Our study demonstrates that frameshifts can be widespread and can be retained for millions of years if they are embedded in a conserved sequence theme.

Keywords

Programmed frameshift, sequence analysis, ancestral state reconstruction, mitochondrion, ND3

Background

Comparative analysis of molecular sequences across the diversity of life allows to discover which molecular mechanisms have been conserved and which have been modified throughout evolution. Insertions or deletions in protein coding genes are usually selected against because they result in frameshifts that disrupt the amino acid coding frame and result in dysfunctional proteins [1]. Albeit rare, examples of corrective frameshifting exist, in which ribosomes regain the proper reading frame [2]. Programmed translational frameshifts have been characterized in viruses, retrotransposons, bacteria, yeast and in

mammalian antizymes (reviewed in [3,4]). Additionally, corrective frameshifting was proposed to be acting in the mitochondria of a range of animals (ants [5], glass sponges [6], oysters [7], birds [8,9] and turtles [9,10]), where +1 frameshift insertions have been reported in up to six different mitochondrially-encoded genes [11] with no clear functional consequences. Alternatively, other mechanisms than corrective frameshifting could explain the absence of functional consequences of the frameshift insertion, such as non-canonical translation of tetra or penta codons, which might be an ancient translation mechanism [12], or RNA editing [13].

Three common sequence features that surround frameshift insertions were identified in the better known examples of translational frameshifts (*Ty1* and *Ty3* genes in yeast, antizyme gene in mammals, *prfB* in *E. coli*); a tRNA that enables the ribosome to “slip” on the ribosome P-site, a rarely used codon in the A-site promoting the stall, and a commonly used codon in the +1 frame [14]. In mitochondrial genes with frameshifts, the mechanism leading to a programmed corrective frameshift is not as well characterized but the DNA sequence surrounding the mitochondrial frameshift insertions has the same features, indicating that the mechanism that facilitates the correction could be the same [11]. Three models have been proposed that may enable translational frameshifting [13]. The “pause-and-slip” model proposes that a pause is induced at the A-site of the ribosome and that the P-site tRNA can pair with the +1 codon, allowing it to slip out of frame [15]. A second model proposes that abnormal tRNA structures enable the frameshift [16]. The “out-of-frame” model proposes that the recruited tRNA skips the additional nucleotide in the A-site [11]. This “out-of-frame” correction of the frameshift is thought to act in glass sponges because of the conserved genomic features in several mitochondrial genes containing frameshifts [13].

The “out-of-frame” model may also apply to the frameshift insertion found in the mitochondrial genomes of certain turtles and birds, where a +1 frameshift insertion occurs at position 174 of the NADH dehydrogenase 3 gene (hereafter ND3-174+1)[9]. The nucleotide sequence around the ND3-174+1 insertion is conserved in a way that would facilitate “out-of-frame” pairing [13]. The insertion is usually found in a codon CUN, with N being the insertion, which produces a wobble pairing with tRNA-leucine when entering the P-site of the ribosome [11]. The codon downstream of the insertion is usually AGU, a rarely used codon for serine, which enters the A-site [11]. The CUN in the P-site and the AGU in the A-site are thought to initiate the frameshift correction by causing a stall in decoding [11]. The AGU codon further forms the beginning of a 15 base pair long stem-loop RNA secondary structure [9], which may enhance the stall [11]. After the stall has been initiated, there are two possibilities. One is to maintain the shifted reading frame, which would lead to an early termination of the protein (in birds ending after 207 bp instead of the usual 354 bp [9]), or to produce a +1 frameshift, leaving out A-175 and thus recovering the regular reading frame of the protein.

ND3-174+1 was the first mitochondrial +1 frameshift that was described in vertebrates, initially described in ostrich (*Struthio camelus*) [8]. An extended investigation found that the ND3-174+1 insertion was also present in a species of turtle and in many other bird species (46/61 bird species) [9]. Within turtle mitogenomes, the insertion was widespread (27/31 turtle species), likely present in their ancestor but lost two to three times within turtles, while being absent in crocodiles (2 species) and snakes (1 species) [11]. From this pattern of absence and presence, it was suggested that the insertion could have been present in a common ancestor of turtles and birds (i.e. Archelosauria: birds+crocodiles, and turtles [17]) and subsequently lost in crocodiles, and lost again in specific lineages of turtles and birds [11]. The insertion was not observed in any other vertebrate lineage [9]. The relatively

small number of species investigated in each vertebrate group limited the resolution of the evolutionary history of the insertion. Denser sampling promises to provide additional insight into the distribution of the frameshift insertion in different lineages, and to determine not only the common features of the sequence theme that allow the insertion to remain in the genome, but also find potential deviations from a conserved sequence theme.

Here, we build on the abundance of mitochondrial sequence data publicly available for vertebrates to study the evolution of the frameshift insertion in the mitochondrial protein coding gene ND3. We compiled ND3 sequences representing 10,309 vertebrate taxa and reconstructed ancestral states and transformations of ND3-174+1 in different groups of Diapsida. We further investigated sequence conservation and codon usage patterns around the insertion site to identify potential common sequence motifs associated with the absence or presence of the frameshift insertion. This large dataset provides improved resolution to understand the evolution of this frameshift insertion and highlights that common sequence patterns appear to be required for maintaining the programmed translational frameshift.

Data description

In this work, we have compiled a total of 87,708 ND3 sequences from 10,397 unique vertebrate taxa (data available in GigaDB[18]). Even though our focus was to represent ND3 sequences for vertebrate species, the taxonomy used on NCBI is not always representing formally named species. The ‘Organism’ field in NCBI mostly contains traditional Linnaean names for species or subspecies, but in some cases the field contains unnamed taxa such as undescribed species, cryptic species or uncertain species identification (designated with sp., cf. or a placeholder name). Although some of the unique sequence identifiers may not present distinct species, we include these taxa here

in addition to the formally named species to represent a diversity of patterns in ND3. This decision also allowed us to match each unique sequence identifier to a leaf in the Open Tree of Life phylogenetic tree (see Methods), which also integrates NCBI's taxonomy. Sequences were extracted from fragments of ND3 sequences and partial or full mitochondrial genomes downloaded from NCBI's GenBank and RefSeq databases. We reduced the dataset to one representative sequence for each unique entry in the Organism field for the following analyses. If multiple sequences for the same taxon existed, we confirmed that all sequences agreed in the presence or absence of the insertion at position 174. Among intraspecific records, we gave sequences from RefSeq preference over GenBank sequences. Among multiple GenBank records for the same taxon, a random sequence was chosen. This resulted in a dataset of 10,397 vertebrate taxa, which was further filtered to exclude ambiguously aligned sequences to a total of 10,309 taxa for further analysis (GigaDB - ND3_Table_uniqueSP).

Analysis

Patterns of presence and absence of ND3-174+1 in vertebrates

Out of the 10,309 sequences for ND3, a normal reading frame of ND3 without an insertion at position 174, was found in all included sequences of jawless fishes (Cyclostomata, N=30 taxa included), cartilaginous fishes (Chondrichthyes, N=208), lungfishes (Dipnoi=5), bony fishes (Actinopterygii, N=3,511), coelacanths (Actinistia, N=2), amphibians (Amphibia, N=627), mammals (Mammalia, N=1,683), snakes, lizards, worm lizards and tuatara (Lepidosauria, 305) and crocodiles (Crocodylia, N=22). A single nucleotide insertion in position 174 was observed in some lineages of turtles (Testudines, N=141; 98 with insertion) and some lineages of birds (Aves, N=3,775; 827 with insertion).

Because the insertion was only found in birds and turtles, we focused further analyses on Diapsida (gene alignment in Additional_file_1 and ND3-174 status in Additional_file_2). We obtained a consensus phylogenetic tree from the Open Tree of Life [19] for 3,464 taxa (Additional_file_3), including Archelosauria (birds (N=3,063), crocodiles (N=22), turtles (N=126)), and their sister group Lepidosauria (N=253) (Figure 1A) (named tips in Additional_file_5). The insertion was absent in 2,654 diapsid taxa, while ND3-174+1 was present in 811 bird and turtle taxa. The inserted nucleotide was cytosine (C) in 749 taxa, thymine (T) in 53, guanine (G) in 7 and adenine (A) in 2 taxa.

We used ancestral state reconstruction using maximum parsimony (MP) and maximum likelihood (ML) with an equal rates model to infer the likelihoods of the presence (any nucleotide) or absence (gap in the alignment) of the insertion at each node in the tree. Branch lengths were set to equal lengths because the Open Tree of Life tree does not include branch lengths. Absence of the ND3-174+1 insertion was inferred in both MP and ML frameworks as the likely ancestral state for the common ancestor of Diapsida, the common ancestor of lizards and snakes (Lepidosauria), the common ancestor of birds and crocodiles (Archosauria), and the common ancestor of Archosauria and turtles (Archelosauria) with high probability (likelihood of absence > 0.99, Figure 1A). The common ancestor of birds (Aves) was inferred to have contained the insertion (likelihood of presence 0.98). The common ancestor of turtles (Testudines) was reconstructed without the insertion (likelihood of absence 0.97).

In order to quantify the number of state changes between absence and presence of the insertion, we counted the number of transitions across nodes. We chose a conservative approach for inferring a transition, which required that both parent and child nodes had a minimal likelihood > 0.90 for a different state. Nodes with a likelihood < 0.90 in the ML

reconstruction also had multiple parsimonious solutions in the MP framework. The MP and ML models therefore inferred the same number of gains and losses. Within turtles and birds, a total of 33 gains and 47 losses of the ND3-174+1 insertion were inferred.

In order to identify the specific nucleotides involved in the insertion and their transitions, we reconstructed ML ancestral states using the five possible states (gap, A, T, C, G) and counted state changes as above. The five state model was able to successfully resolve many nodes that were not resolved in the two state model, which resulted in higher inferred gains and losses of the insertion under the model with five states (38 gains and 52 losses). The gain of the insertion was most frequently a C (34 gains), followed by T (4 gains). Loss of the insertion happened most frequently from C (50 losses), followed by T (2 losses). When the insertion was present, most transitions were from C to T (27 transitions), while other state changes were less frequent (2 transversions from C to G, 1 transversion from C to A, 1 transversion from T to A, 1 transition from T to C).

Complex patterns of gain and loss within turtles and birds

Within turtles, 87 of 126 examined sequences had the insertion and they were dispersed across the phylogeny (Figure 1A). Using the two state models, we inferred four losses within turtles, but no gains could not be unambiguously estimated because the nodes did not have likelihoods > 0.90 in the ancestral state reconstruction. The model using five states inferred a complex evolution of the with three inferred gains (likelihood > 0.90), of which two were insertions of C and one was an insertion of T (Figure 1B). The five state model inferred five losses of the insertion, of which three losses were from an ancestral C state and two losses were from an ancestral T state (Figure 1B). Transitions from C to T were most common (7 transitions), compared to other transitions (1 T to C) and transversions (1 T to A). An insertion of G was observed in turtles in a clade of three

species (*Malaclemys terrapin, Trachemys scripta, Chrysemys picta* in Emysternia), but the ancestral state could not be inferred unambiguously (Figure 1B).

Within birds, 724 of 3,063 examined sequences had the insertion. The common ancestor of birds was inferred to have had an insertion of C (likelihood 0.99). The two state models predicted 32 gains and 49 losses, while the five state model inferred 34 gains and 47 losses (likelihood > 0.90). Gains were mostly of C (31 gains) or of T (3 gains). As in turtles, transitions from C to T (20 transitions) were most common, while other state changes were less frequent (2 transversions from C to G, 1 transversion from C to A). Different orders of birds had different prevalence of the ND3-174+1 insertion or its absence (Figure 1C). The only two orders consistently without the frameshift insertion were the speciose perching birds (Passeriformes, N=2,096 included) and the tropicbirds (Phaethontiformes, N=2) and their ancestors were reconstructed without the insertion (likelihood 0.99). The insertion was present in all examined sequences of 12 orders (Pterocliformes, Columbiformes, Mesitornithiformes, Musophagiformes, Otidiformes, Opisthocomiformes, Eurypygiformes, Suliformes, Cariamiformes, Falconiformes, Coliiformes, Trogoniformes). The remaining bird orders had both lineages with and without the insertion (Figure 1C).

Strong sequence conservation surrounding the insertion

In order to identify a potential shared sequence pattern around the ND3-174+1 insertion, we compared the nucleotide diversity between diapsid sequences with and without the insertion. We calculated information content (R) based on Shannon entropy for each base pair, which measures conservation of a sequence position and has a maximum value of 2 bits if the position is fully conserved. In taxa without the insertion (Lepidosauria, crocodiles, certain turtles and certain birds), the region around position 174 (from 163 to 180 bp) of ND3 had a similar conservation level as the remainder of the gene (Figure 2A). In contrast,

all turtle and bird species with the insertion had noticeably more conserved base pairs (higher R values) around position 174 than in other regions of ND3 (Figure 2A). The distribution of values of R around the insertion from position 163 to 180 was 20% lower in species without the insertion than with the insertion (non-parametric Wilcoxon test, p-value $<10^{-3}$). Species with the insertion showed a mean information content 20% lower than species without the insertion (Figure 2A). Specifically, when the insertion was present, nucleotides upstream of the insertion (position 163 to 174) were highly conserved with some variability on the third codon positions (Figure 2B). The sequence downstream of the insertion (position 175 to 180) was completely conserved with a maximum information content (R=2 bits, Figure 2B). When the insertion was absent, sequence conservation was lower (R<2 bits), particularly on the third codon positions downstream of 174 (Figure 2C).

We also analyzed the codon conservation in sequences containing the insertion (a combined set of turtles and birds with the insertion). The codon containing the insertion was a leucine codon (CTN) in all examined bird and turtle species, with CTC being most prevalent and CTT, CTG and CTA represented at lower frequencies (Figure 2D). Notably, the green wood hoopoe (*Phoeniculus purpureus*, Bucerotiformes) deviated from this pattern with an ATC encoding for isoleucine. Both leucine and isoleucine are nonpolar amino acids. For the codons following the insertion, we considered both the 0 reading frame, which is the shifted reading frame if the frameshift insertion was retained, and the +1 corrected reading frame, which is the frame after the A at position 175 is left out [11]. In the 0 reading frame, the first two codons downstream of the insertion showed almost complete conservation to AGT (encoding serine, a polar amino acid) in the first codon following the insertion (position 175-177) and AGC (encoding serine, a polar amino acid) in the second codon following the insertion (position 178-180) (Figure 2D). The only exception to this high codon conservation following the insertion was Baillon's crake

(*Porzana pusilla*, Gruiformes) with a CGT codon (encoding arginine, a basic amino acid) in the first following codon. In the +1 corrected reading frame leaving out A-175, the codon following the insertion (position 176-178) was a GTA codon (encoding valine, a non-polar amino acid) in all sequences (Figure 2D). The second codon following the insertion (position 179-181) was also conserved in coding for alanine (a non-polar amino acid), albeit with all four synonymous codons present (Figure 2D).

No major tRNA changes in taxa with the insertion

We investigated whether lineages containing the ND3 insertion showed differences in tRNA secondary structure, which could, potentially, enhance programmed frameshifting. We aligned a set of tRNAs of leucine (CUN), serine (UCN) and valine, which are the tRNAs decoding the codon where the insertion occurs (leucine), and the two tRNAs that compete for being decoded downstream of the insertion (serine and valine) (Figure 2D). We compared the predicted consensus secondary structure for each tRNA from four alignments, turtles and bird with and without ND3-174 (Additional_file_6). We did not observe consistent differences in the secondary structure between bird and turtles with the frameshift insertion.

Additional potential frameshifts in five turtle species

The translational machinery of certain birds and turtles seems to enable programmed frameshifting in order to correct single nucleotide insertions in coding regions and additional frameshift locations could exist. Using a subset of Diapsida sequences with full mitochondrial genomes, we checked for other frameshifts in ND3 other than ND3-174+1. We did not find evidence for additional frameshift insertions in the ND3 sequences of birds (N=703) nor in other Diapsida groups (N=341), except for turtles. Out of 106 investigated mitochondrial genomes of turtles, we identified five putative frameshifts upstream of

position 174, namely in *Cuora aurocapitata* (A inserted at alignment position 121; NC_009509.1), *Cuora pani* (T deleted at alignment position 136 or 137; NC_014401.1), *Cyclemys oldhami* (possible deletion at alignment around position 116; NC_023220), *Pelomedusa subrufa* (G insertion at position 149; NC_001947.1) and *Pelusios castaneus* (G insertion at position 149; NC_026049.1). The two *Cuora* turtles had frameshifts in different positions. *Pelomedusa subrufa* and *Pelusios castaneus* (both Pelomedusidae) had frameshifts at the same location.

For *Pelusios castaneus*, we were able to verify that the frameshift was not due to sequencing errors by mapping high-throughput genomic sequencing reads (SRR9091361) and transcriptomic reads (SRR629649) to the mitochondrial genome (NC_026049.1). The insertion site was verified both in DNA and RNA short reads (Additional_file_7). We could not perform this check for the frameshift insertions in the other four turtle species due to the lack of additional genomic or transcriptomic data.

Discussion

In this work, we have inferred multiple origins of an insertion in position 174 of the mitochondrially-encoded NADH dehydrogenase 3 complex gene (ND3-174+1) based on a large collection of publicly available sequences for 10,309 vertebrates. This study significantly expands the sampling of previous studies (61 species in [9], 34 species in [11]) to provide a broader picture across vertebrates on one hand and more fine-scale resolution of sequence conservation on the other hand.

We confirm that the insertion is present only in turtles and birds [9,11] but the improved sampling shows that both the insertion was more frequently gained and lost than previously thought. Different from previous interpretations, which predicted the presence of

this insertion in the common ancestor of turtles and birds (Archelosauria), ML and MP ancestral state reconstruction suggested an independent evolution of this insertion in birds and turtles. Within birds, the insertion was reconstructed as present in the most recent common ancestor of modern birds, which lived about 70-111 million years ago (depending on the phylogeny, [20,21]). Once obtained, the insertion was retained in many lineages but lost in the common ancestor of Passeriformes 39-49 million years ago [20–22] and not regained since. Additionally, within the other bird lineages, it appeared more likely to lose the insertion than to gain it (60% of changes are insertion losses, while 40% are insertion gains across all tested models). The most recent common ancestor of turtles was inferred to not have had an insertion in ND3, as opposed to previous ideas [9,11]. Our data included 126 species of turtles, 93 more than in the last study on turtle mitochondrial genomes [11], which produced an alternative interpretation of the gain and loss patterns. Within turtles, the insertion has been independently gained one to three times based on our reconstructions. Within clades that have insertion, some lineages show mutations to other nucleotides. The higher prevalence of C to T transitions both in turtles and birds (27/32, 84% of mutations) could be a consequence of cytosine methylation, which has been described to be present also in mitochondrial genomes [23].

The quality of the ND3 sequences and the observed absence or presence of the insertion at position 174 on these sequences is of crucial importance for our inferences. Most of the ND3 sequences used here originate from Sanger sequenced ND3 genes, whose chromatograms may have been hand-curated for sequencing errors. While insertions at position 174 are likely to be genuine, as they would have been flagged as problematic during submission to NCBI’s Genbank and would require a special annotation to address the frameshift insertion (often to [9]), the absence of the insertion may be overrepresented. A frameshift insertion in ND3 may have been curated out of the sequence because such

insertions in protein coding sequences are extremely rare and could have been considered a sequencing error. It is therefore possible that the estimated number of loss events in turtles and birds is overestimated. Where losses were observed in multiple members of a clade, the most extreme case being the absence in all 2,096 included Passeriformes, the absence of the insertion is likely genuine. To estimate the prevalence of this potential problem, we compared ND3 annotations for 101 bird species that have ND3 sequences on NCBI (mostly Sanger sequenced) and also high-throughput sequenced mitochondrial genomes from the B10K project, for which we have ourselves created the annotations and can therefore exclude manual modification. Reassuringly, we found that there were no cases in which the B10K dataset contained the insertion while the GenBank sequence did not contain it. This assessment admittedly spans only a small fraction of the taxa investigated here but lends support that at least in birds, annotation errors may be limited. It is possible that submitters of bird and turtle sequences are more aware of the possibility of a frameshift insertion because the insertions have been reported from these taxa [9,11] than submitters of taxa in which the insertion has not been observed, such as Lepidosauria or Mammalia.

It is intriguing that an insertion in exactly the same position of the ND3 gene has independently evolved 37 times (ML model; 33 according to the parsimony model), across turtles and birds. This site-specificity points to an underlying common feature that causes the frameshift to occur in this position. One possibility is that there is an increased probability to produce indels in that specific position. Alternatively, insertions may appear at a normal rate but only get tolerated if they are embedded in a specific sequence motif that allows ribosomes to conduct the frameshift correction. This seems likely given the strong conservation at the nucleotide and at the codon level that evolved convergently in birds and turtles, despite their separate evolution for over 240 million years. Intriguingly,

this conservation is not found in birds and turtles without the insertion (Figure 2A) nor in the other diapsids without the insertion, as expected with the degree of divergence of vertebrates. Further, the similar sequence and codon conservation observed around ND3-174+1 have been described in other mitochondrial frameshifts across animals [11]. This indicates that the heavily conserved sequence is needed for correcting the insertion and tolerating it in the mitochondrial genome.

Most of the features observed in this study conformed with the out-of-frame frameshift model [11], which is characterized by a weak codon tRNA interaction in the P-site, a rare codon downstream, and an alternative codon if the reading frame is restored which achieves a canonical Watson-Crick match with its tRNA [13]. Our extended sampling provides higher resolution of the sequence conservation features that may be involved in the programmed translational frameshift, adding examples of species that deviate from the most commonly used codons. This codon upstream from the translational frameshift position was proposed to produce a wobble pairing initiating a translation stall [11]. We found it to behighly conserved as a leucine codon (CTN) in all examined bird and turtle species with the exception of one bird (green wood hoopoe *Phoeniculus purpureus*) that uses an isoleucine codon (ATC). We further confirm that a serine codon (AGT), a polar amino acid, was always observed after the insertion [11]. However, we also show that this pattern can be more flexible, within at least one bird (Baillon's crake *Porzana pusilla*) using an arginine codon (CGT), a basic amino acid (Figure 2D). The AGT codon following the insertion is thought to further promote the stall in the translation, which in turn facilitates the frameshift because it is a rarely used codon for serine [11]. If the observed variable codon, CGT, was also a rarely used codon for arginine, the hypothesis that rare codons promote the translation stall would still hold. In human mitochondria, the CGT codon is indeed only the third out of four possible codons for arginine in codon preference [24],

indicating that CGT is a rare codon. If CGT was also rarely used in the other vertebrates, it could be an additional example of a rare codon to promote the stall in the translation.

Regarding the insertion itself, we have observed all four nucleotides to be present in different frequencies. According to the out-of-frame frameshift model, wobble pairing between a sequence and the tRNA anticodon promotes frameshifting [11]. Consequently, the insertion should rarely occur as an adenosine because a CTA codon produces a perfect match with the tRNA-leucine anticodon [11]. In addition to Reeve's turtle (*Chinemys reevesi*), which has previously been shown to contain an A-insertion [11], we found an independent occurrence of A-insertion in a bird, Baillon's crake (*Porzana pusilla*), which also has a non-synonymous codon right after the insertion as described above. These two species may therefore be interesting candidates for further investigations on the programmed translational frameshift in the absence of wobble pairing.

In addition to the detailed investigation of position 174, we found four additional frameshifts in ND3 in five turtles. Of these, the African helmeted turtle (*Pelomedusa subrufa*) was already described to contain such a frameshift [11] and the frameshift of the West African mud turtle (*Pelusios castaneus*) in the same position was annotated as a frameshift insertion on NCBI. These two species are Pelomedusidae and both *Pelusios* and *Pelomedusa* contain a number of species [25], which could be sequenced for ND3 to investigate if the insertion is shared across Pelomedusidae or independently obtained in the two species. The other three frameshifts are, to the best of our knowledge potential new frameshifts in the ND3 gene. These findings support previous observations on the ubiquitous presence of such frameshifts in other mitochondrial genes of turtles [11], suggesting a broader tolerance of turtles to frameshift insertions.

Our study demonstrates that incorporating a large number of sequences can improve resolution in inferred evolutionary patterns and give additional power to investigate sequence conservation. Our analyses suggest an independent origin of the frameshift insertion both in turtles and birds, and complex patterns of gains and losses within each group. The high sequence conservation surrounding the insertion suggests purifying selection retaining the sequence motifs needed for translational frameshifting. Nonetheless, a few species deviate from the conserved pattern. Additional losses and gains of the insertion and other deviations from the conserved motifs will likely be found once more ND3 sequences become available, within birds and turtles and possibly also in other vertebrates.

Potential implications

The current work advances our understanding of the distribution of the frameshift insertion in the mitochondrial gene ND3 across the vertebrate tree of life and identifies highly conserved sequence features that seem to be associated with its occurrence. This will allow researchers to further study which sequence features allow for the corrective frameshift and to investigate the evolutionary constraints that keep the surrounding sequence heavily conserved. The fact that this insertion has remained in the mitochondrial genome for millions of years in certain birds and turtles opens the door to study the translational machinery in these lineages.

Methods

Dataset preparation

RefSeq mitochondrial genomes were downloaded from the NCBI FTP site [26], resulting in 5,325 mitochondrial genomes with the term 'Vertebrata' contained in the taxonomy, from

which we could retrieve the ND3 sequence in 5,320 records. We searched for nucleotide sequences containing the ND3 gene on the GenBank nucleotide database (accessed 2019-12-11) with the query "NADH dehydrogenase subunit 3" AND Vertebrata[Organism] AND mitochondrion[filter]". A total of 92,352 sequence records were downloaded using a custom script. From those, we were able to find 88,880 records of ND3 sequences. We further included mitochondrial data from the second phase of the Bird 10,000 genome project (B10K), which produced 336 mitochondrial genomes as part of their whole genome sequences [27]. Of these mitochondrial genomes, we included 207, which had ND3 sequences assembled and which were added if the species was not already present in the database.

Sequence alignment

One ND3 sequence for each unique taxon (10,397) was aligned using MAFFT FFT-NS-1 (v7.407) for initial alignment [28] (GigaDB - ND3_aligned1_uniqueSp.fa). Next, sequences were pruned from positions not seen in at least 5% of the sequences using pxclsq (v0.1) (GigaDB -ND3_filtered1_uniqueSp.fa) from the phyx suite [29] to exclude insertions only seen in a small proportion of taxa. Note that we also explicitly investigated other frameshifts than ND3-174+1 across ND3 in birds and turtles (section Other possible frameshift insertions), while this alignment was focused on the region around position 174. To remove potential low quality sequences around the insertion, we removed sequences that contained a gap or an N in the two codons (six nucleotides) upstream and downstream of the insertion and records that after visual examination were not properly aligned around ND3-174. We performed a second alignment round on the remaining sequences with a slower and more accurate mode of MAFFT (L-INS-i). We repeated filtering positions not in at least 5% of sequences and repeated quality filters around

position 174. The final filtered alignment of 10,312 sequences is given in GigaDB (ND3_filtered2_uniqueSp.fa).

To confirm that all sequences of the same unique taxon had the same pattern at position 174, we used the filtered alignment as a framework for aligning all other intraspecific records, by using the --add function from MAFFT and applying the same filters as above (GigaDB-ND3_filtered.fa).

Phylogenetic distribution of the insertion

The frameshift insertion in position 174 was only observed in certain species of turtles and birds and we therefore restricted analyses to Diapsida, i.e. birds, crocodiles, turtles and Lepidosauria (tuatara, worm lizards, snakes and lizards). This left 4,233 out of 10,397 vertebrate taxa. We recorded the state of position 174 in each sequence, either being a gap in the alignment (i.e. insertion absent), or being a nucleotide (i.e. insertion present as A,T,C,G).

We used the R package rotl (v3.0.10) [30] to obtain a phylogenetic tree for the included species. The package queries the Open Tree of Life (otol) database [31], which synthesizes phylogenetic hypotheses from published datasets and adds species that have not been included in phylogenetic analyses based on the taxonomic system [19,32]. While a fully sampled tree for Diapsida would be preferable over a synthetic tree, it agrees in the relationships among the major Diapsida clades with phylogenetic analyses [33–36]. Of the 4,233 Diapsida species with ND3 records, 3,465 could be matched with a terminal on the Open Tree of Life (newick tree in Additional_File_3). In order to summarize the distribution of absence or presence of the insertion on a dated bird phylogeny, we used the fossil-calibrated phylogenetic tree from [20].

Ancestral state reconstruction

Maximum likelihood (ML) ancestral states were reconstructed using the R package Castor (v1.5.5) [37] using the function hsp_mk_model. The function first calculates the transition matrix between different states assuming equal-rates for transitioning from one state to another and vice versa in a ML framework. We chose the equal-rates model because it makes the least assumptions about the probabilities of gain, loss and transitions between different states. Given the known states of the tips and the phylogenetic tree, the likelihood of each node in the tree was calculated using the rerooting method [38]. Because the Open Tree of Life synthetic phylogeny did not include branch lengths, the function assumes equal branch lengths throughout the phylogeny. ML analyses were both done for two states (absence or presence of the insertion) and for the five possible states (absence, A, C, G, T).

Maximum parsimony (MP) reconstruction of ancestral states was performed with the function MPR in the ape package [39]. MP analysis was done for two states (absence or presence of the insertion). Prior to analysis, polytomies were arbitrarily resolved using the function multi2di in ape.

Inference of transitions between states

In order to count the number of nodes of the phylogeny where transitions from one state to another likely occurred, we related the likelihood from the ancestral state reconstruction of each descendant node to its parent node using the R package phangorn [40]. In the ML model, we only considered nodes with an ancestral state likelihood > 0.9. If the likelihood was < 0.9, the state was considered as ambiguous. A transition was counted when a descendant node differed from its parent's state with high likelihood. This approach therefore only identifies transitions that are accompanied with strong changes in

likelihoods. On the other hand, in the MP model, we counted transitions where both the insertion status parental and child nodes could be resolved unambiguously. We counted the number of transitions between absence and presence of the insertion in both the ML and MP model with two states and the number of transitions between the five states (absence, A, C, G, T) in the ML model.

Sequence conservation and codon usage

Nucleotide frequencies per position across the entire ND3 sequence were obtained separately for diapsids without the insertion and with the insertion. We calculated Shannon entropy as a measure of nucleotide diversity [9]:

$$H_i = -\sum_N freq_N \; x \; log_2(freq_N)$$

where *H* is the Shannon entropy in position i of the DNA sequence, $freq_N$ is the frequency of nucleotide *N* of state {A,T,G,C}. Shannon entropy was transformed into information content per nucleotide position:

$$R_i = log_2(4) - H_i$$

where R is the information content at position i [41]. The information content was compared between the two groups in the region surrounding the insertion (position 163-180) with a non-parametric Wilcoxon test and a significance threshold (α) of $p<0.05$. Weblogo [42] was used to visualize the information content of this region (position 163-181) for both groups, where the relative diversity versus conservation of each nucleotide is reflected as the height of the nucleotide, measured in bits with a maximum value of 2 at complete conservation.

Codon frequencies were calculated for both the shifted reading frame, the 0 reading frame, and for the corrected reading frame, the +1 reading frame. Codons containing unknown nucleotides (N) were removed. Codon frequencies were calculated for the six codons surrounding the insertion (position 163-181). The calculation of codon frequencies in the

+1 reading frame excluded the adenosine at position 175 (A-175) following the insertion [11].

tRNA secondary structure prediction

The sequences for three tRNAs involved in translation of codons surrounding the insertion were extracted from the compiled set of full mitochondrial genomes of birds and turtles. We extracted the tRNA for leucine, which translates the codon upstream of the insertion; serine, which translates the codon following the insertion in the shifted reading frame; and valine, which translates the codon following the insertion in the corrected reading frame. We split the sequences in a group of birds and a group of turtles, each with and without the insertion. Short sequences were removed if they were between 2 times the standard deviation of the mean nucleotide length of the group. An initial alignment was done using the mlocarna tRNA aligner (v2.0.0RC8) [43], which simultaneously aligns and folds RNA sequences. Two tRNAs exist for leucine and serine in mitochondrial genomes, of which CUN-leucine and UCN-serine are involved in translating the codons around the ND3-174+1. Because Genbank records do not always distinguish between the two tRNAs, we kept the tRNA copy with the higher pairwise identity to a known representative CUN-leucine and UCN-serine tRNA from the initial alignment. The filtered sequences were realigned with mlocarna. The predicted consensus secondary structure of the tRNA alignment was visualized using the ViennaRNA web services [44].

Other possible frameshift insertions in ND3

In order to extend the study to other potential insertions in the ND3 gene in Diapsida, we compiled a dataset of 1,050 complete mitochondrial genomes from the Refseq database. We excluded records without annotations or with undetermined nucleotides (N) in the ND3 gene, resulting in 1,044 ND3 gene sequences (Crocodilia N=20, Lepidosauria N=215,

Testudines N=106, Aves N=703). As a further check for consistency, we also investigated a second dataset focused on birds belonging to the B10K project including 328 mitochondrial genomes that were *de novo* assembled from shotgun genomic reads [27].

Both ND3 sequence sets were aligned using the vertebrate mitochondrial genetic codon table (“-gc_def 2”) in the “alignSequence” module of MACSE (v2.01) [43], which respects reading frames and can tolerate frameshifts (Additional_file_4). MACSE designates frameshifts with the symbol ‘!’ and candidates were visually verified with the alignment viewer Seaview (v5.0.4) [46]. Individual ND3 gene sequences were also annotated using “protein2genome” model of exonerate (v2.4.0) [45] by mapping the *Gallus gallus* ND3 amino acid sequence (NC_040902.1) to each of the diapsid ND3 nucleotide gene sequences. Exonerate designates potential frameshifts with the symbol ‘#’.

Availability of source code and requirements

Scripts used for data generation and analysis can be found at:
https://github.com/sergioSEa/ND3_174_vertebrates2020
Operating system(s): e.g. Bash scripts should be run in Linux OS/Mac OS. Python and R scripts are platform independent.
Programming language: Bash, R, Python
Other requirements: Python 3 or higher, Mafft v7.4, pxclsq v0.1. Python packages: biopython. R packages: rotl, castor, ape, phytools, ggtree, ggimage, phangorn, ggstance, Biostrings, ggrepel and tidyverse.
License: GNU

Availability of supporting data and materials

The data sets supporting the results of this article are available in the GigaScience database GigaDB [18].

List of abbreviations

ND3 - NADH dehydrogenase 3 complex gene

A - adenosine

C - cytosine

T - thymine

G - guanine

N - undetermined nucleotide

ML - maximum likelihood

MP - maximum parsimony

NGS - Next generation sequencing

Author's contributions

Sergio Andreu-Sánchez: Formal analysis, writing, visualization. conceptualization; Wanjun Chen: data curation, formal analysis, visualization; Josefin Stiller: Conceptualization, supervision, visualization, writing; Guojie Zhang: Conceptualization; supervision; resources; writing

Acknowledgements

This project was supported by Carlsberg Foundation (CF16-0663). It was partially supported by the Strategic Priority Research Program of the Chinese Academy of Sciences (XDB31020000). GZ is also supported by Villum Foundation (No. 25900).

Additional material

Additional_file_1: **Diapsida MAFTT alignment.** Diapsida MAFFT alignment after removal of positions not seen in at least 5% of taxa.

Additional_file_2: **Diapsida Table**. Table of Diapsida taxa and the corresponding status at ND3-174 used for ancestral state reconstruction extracted from Addtional_file_1.

Additional_file_3 **Diapsida tree.** Open tree of life matched to the Diapsida taxa included in the study.

Additional_file_3: **Diapsida MACSE alignment**. Alignment of 1,044 Refseq Diapsida sequences used for identification of other frameshifts.

Additional_file_4: **Labelled diapsida tree.** Diapsida tree as presented in Figure 1A but including taxa names. PDF file.

Additional_file_5: **Consensus tRNA structure**. Consensus predicted tRNA structure of birds and turtles with and without gap from Valine, Leucine and Serine. Nucleotides presented do not represent the real consensus sequence.

Additional_file_6: **NGS read mapping to *Pelusios castaneus***. Genomic (SRR9091461) and transcriptomic (SRR629649) NGS reads were mapped to *Pelusios castaneus* mitochondrial genome (NC_026049.1) to check whether the predicted frameshift site was present.

Figures and legends

Figure 1. Phylogenetic distribution of the insertion in position 174 of the mitochondrial ND3 gene (ND3-174+1). (a) Synthetic phylogeny of 3,464 species of Diapsida with terminals colored according to the absence (orange) or presence (blue) of the insertion. (b) Ancestral state reconstruction for turtles with states of species indicated at the tips and pie charts showing the likelihoods of different states on nodes of the phylogeny. (c) Frequency of absence (orange) or presence (blue) of the insertion in each bird order. Numbers represent the number of species that were included for each order.

Figure 2. **Nucleotide and codon usage variability in ND3 of Diapsida.** (a) Information content R (cubed for visualization) across the ND3 sequence in different diapsid groups. The vertical red line marks the insertion at position 174. The red shading highlights an

area of high conservation (high information content) only seen in birds and turtles that have the insertion. (b-c) Sequence conservation as a sequence logo from position 163 to 180 showing variability among species (b) with the insertion and (c) without the insertion. Note that the frameshift correction is thought to occur at the following base, by skipping the nucleotide A at position 175. (d) Circle packing showing the frequency of codon usage for each codon in species that contain the insertion. The two options of the shifted and corrected reading frame following the insertion at position 174 are shown. Circle diameters indicate prevalence of a specific codon, which are grouped into larger circles if codons are synonymous. Circle color indicates amino acid class.

References

1. Tse H, Cai JJ, Tsoi H-W, Lam EP, Yuen K-Y. Natural selection retains overrepresented out-of-frame stop codons against frameshift peptides in prokaryotes. *BMC Genomics*.2010;11:491

2. Atkins JF, Loughran G, Bhatt PR, Firth AE, Baranov PV. Ribosomal frameshifting and transcriptional slippage: From genetic steganography and cryptography to adventitious use. *Nucleic Acids Res*. 2016; 44:7007–78

3. Farabaugh PJ. Programmed translational frameshifting. *Microbiol Rev*. 1996; 60:103–34

4. Dinman JD. Mechanisms and implications of programmed translational frameshifting. *Wiley Interdiscip Rev RNA*. 2012;3:661–73

5. Beckenbach AT, Robson SKA, Crozier RH. Single nucleotide +1 frameshifts in an apparently functional mitochondrial cytochrome b gene in ants of the genus *Polyrhachis*. *J Mol Evol*. 2005;60:141–52

6. Rosengarten RD, Sperling EA, Moreno MA, Leys SP, Dellaporta SL. The mitochondrial genome of the hexactinellid sponge *Aphrocallistes vastus*: Evidence for programmed translational frameshifting. BMC genomics 2008;9.1: 1-10.

7. Milbury CA, Gaffney PM. Complete mitochondrial DNA sequence of the eastern oyster *Crassostrea virginica*. *Mar Biotechnol*. 2005;7:697–712

8. Härlid A, Janke A, Arnason U. The mtDNA sequence of the ostrich and the divergence between paleognathous and neognathous birds. *Mol Biol Evol*. 1997;14:754–61

9. Mindell DP, Sorenson MD, Dimcheff DE. An extra nucleotide is not translated in mitochondrial ND3 of some birds and turtles. *Mol Biol Evol*. 1998;15:1568–71

10. Parham JF, Macey JR, Papenfuss TJ, Feldman CR, Türkozan O, Polymeni R, et al.. The phylogeny of Mediterranean tortoises and their close relatives based on complete mitochondrial genome sequences from museum specimens. *Mol Phylogenet Evol*. 2006;38:50–64

11. Russell RD, Beckenbach AT. Recoding of translation in turtle mitochondrial genomes: programmed frameshift mutations and evidence of a modified genetic code. *J Mol Evol*. 2008;67:682–95

12. Seligmann H, Warthi G. Chimeric translation for mitochondrial peptides: Regular and expanded codons. *Comput Struct Biotechnol J*. 2019;17:1195–202

13. Haen KM, Pett W, Lavrov DV. Eight new mtDNA sequences of glass sponges reveal an extensive usage of +1 frameshifting in mitochondrial translation. Gene. 2014;535.2: 336-344.

14. Baranov PV, Gesteland RF, Atkins JF. Recoding: translational bifurcations in gene expression. *Gene*. 2002;286:187–201

15. Huang Y, Koonin EV, Lipman DJ, Przytycka TM. Selection for minimization of translational frameshifting errors as a factor in the evolution of codon usage. *Nucleic Acids Res*. 2009;37:6799–810

16. Sroga GE, Nemoto F, Kuchino Y, Björk G. Insertion (sufB) in the anticodon loop or base substitution (sufC) in the anticodon stem of tRNA Pro2 from *Salmonella typhimurium* induces suppression of frameshift mutations. *Nucleic Acids Res*.1992; 20:3463–9

17. Crawford NG, Parham JF, Sellas AB, Faircloth BC, Glenn TC, Papenfuss TJ, et al.. A phylogenomic analysis of turtles. *Mol Phylogenet Evol*. 2015;83:250–7

18. Sergio A-S, Josefin S, Wanjun C, Guojie Z. Supporting data for "Multiple origins of a frameshift insertion in a mitochondrial gene in birds and turtles." GigaScience Database 2020 http://dx.doi.org/10.5524/100839

19. Redelings BD, Holder MT. A supertree pipeline for summarizing phylogenetic and taxonomic information for millions of species. *PeerJ*. 2017;5:e3058

20. Jarvis ED, Mirarab S, Aberer AJ, Li B, Houde P, Li C, et al.. Whole-genome analyses resolve early branches in the tree of life of modern birds. *Science*. 346:2014;1320–31

21. Prum RO, Berv JS, Dornburg A, Field DJ, Townsend JP, Lemmon EM, et al.. A comprehensive phylogeny of birds (Aves) using targeted next-generation DNA sequencing. *Nature*. 2015;526:569–73

22. Oliveros CH, Field DJ, Ksepka DT, Barker FK, Aleixo A, Andersen MJ, et al.. Earth history and the passerine superradiation. *Proc Natl Acad Sci U S A*. 2019;116:7916–25

23. Sirard M-A. Distribution and dynamics of mitochondrial DNA methylation in oocytes, embryos and granulosa cells. *Sci Rep*. 2019;9:11937

24. Jia W, Higgs PG. Codon usage in mitochondrial genomes: Distinguishing context-dependent mutation from translational selection. *Mol Biol Evol*. 2008;25:339–51

25. Fritz U, Branch WR, Hofmeyr MD, Maran J, Prokop H, Schleicher A, et al.. Molecular phylogeny of African hinged and helmeted terrapins (Testudines: Pelomedusidae: *Pelusios* and *Pelomedusa*). *Zool Scr*. 2011;40:115–25

26. Refseq. ftp://ftp.ncbi.nlm.nih.gov/refseq/release/mitochondrion/. Accessed 2019-12-03

27. Feng S, Stiller J, Deng Y, Armstrong J, Fang Q, Reeve AH, et al.. Dense sampling of bird diversity increases power of comparative genomics. *Nature*.2020; 587:252–7

28. Katoh K, Standley DM. MAFFT multiple sequence alignment software version 7: Improvements in performance and usability. *Mol Biol Evol*. 2013;30:772–80

29. Brown JW, Walker JF, Smith SA. Phyx: Phylogenetic tools for unix. *Bioinformatics*. 2017;33:1886–8

30. Michonneau F, Brown JW, Winter DJ. rotl: An R package to interact with the Open Tree of Life data.*Methods Ecol Evol*. 2016;7:1476–81

31.Open Tree of Life https://tree.opentreeoflife.org/opentree/argus/opentree12.3@ott93302 Accessed 2019-12-03

32. Rees JA, Cranston K. Automated assembly of a reference taxonomy for phylogenetic data synthesis. *Biodivers Data J*. 2017;5: e12581.

33. Green RE, Braun EL, Armstrong J, Earl D, Nguyen N, Hickey G, et al.. Three crocodilian genomes reveal ancestral patterns of evolution among archosaurs. *Science*. 2014;346:1254449

34. Irisarri I, Baurain D, Brinkmann H, Delsuc F, Sire J-Y, Kupfer A, et al.. Phylotranscriptomic consolidation of the jawed vertebrate timetree. *Nat Ecol Evol*. 2017;1:1370–8

35. Chiari Y, Cahais V, Galtier N, Delsuc F. Phylogenomic analyses support the position of turtles as the sister group of birds and crocodiles (Archosauria). *BMC Biol*. 2012;10:65

36. Crawford NG, Faircloth BC, McCormack JE, Brumfield RT, Winker K, Glenn TC. More than 1000 ultraconserved elements provide evidence that turtles are the sister group of archosaurs. *Biol Lett*. 2012;8:783–6

37. Louca S, Doebeli M. Efficient comparative phylogenetics on large trees. *Bioinformatics*. 2018;34:1053–5

38. Yang Z, Kumar S, Nei M. A new method of inference of ancestral nucleotide and amino acid sequences. *Genetics*. 1995;141:1641–50

39. Paradis E, Claude J, Strimmer K. APE: Analyses of Phylogenetics and Evolution in R language. *Bioinformatics*. 2004;20:289–90

40. Schliep KP. phangorn: Phylogenetic analysis in R. *Bioinformatics*. 2011;27:592–3

41. Schneider TD, Stephens RM. Sequence logos: A new way to display consensus sequences. *Nucleic Acids Res*. 1990;18:6097–100

42. Crooks GE, Hon G, Chandonia J-M, Brenner SE. WebLogo: A sequence logo generator. *Genome Res*. 2004;14:1188–90

43. Smith C, Heyne S, Richter AS, Will S, Backofen R. Freiburg RNA Tools: A web server integrating INTARNA, EXPARNA and LOCARNA. *Nucleic Acids Res*. 2010;38:W373-7

44. Kerpedjiev P, Hammer S, Hofacker IL. Forna (force-directed RNA): Simple and effective online RNA secondary structure diagrams. *Bioinformatics*. 2015;31:3377–9

45. Ranwez V, Harispe S, Delsuc F, Douzery EJP. MACSE: Multiple Alignment of Coding Sequences accounting for frameshifts and stop codons. *PLoS One*. 2011;6:e22594

46. Gouy M, Guindon S, Gascuel O. SeaView version 4: A multiplatform graphical user interface for sequence alignment and phylogenetic tree building. *Mol Biol Evol*. 2010;27:221–4

Figure

Click here to access/download;Figure;Figure1.pdf

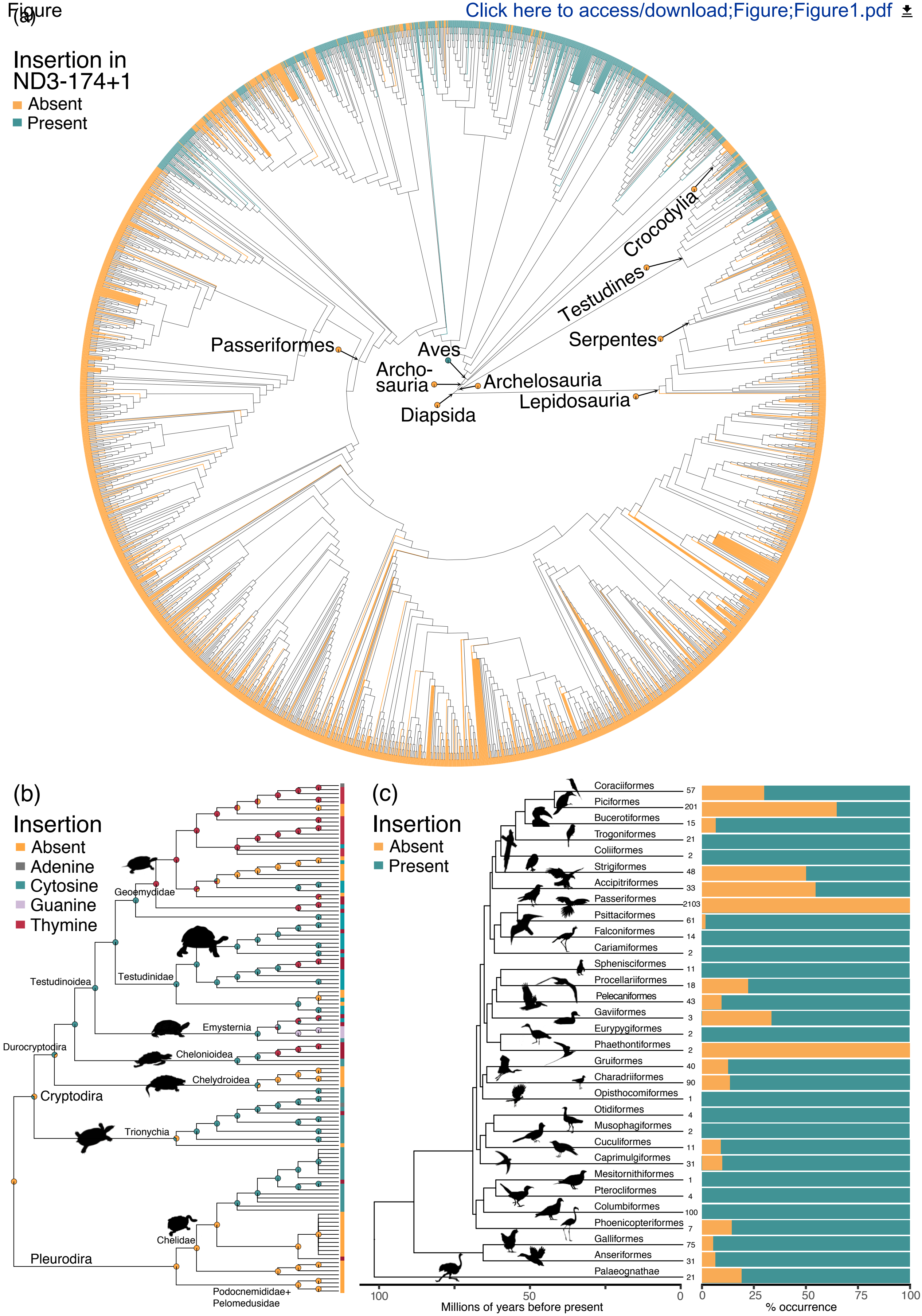

Figure
Click here to access/download;Figure;Figure2.pdf

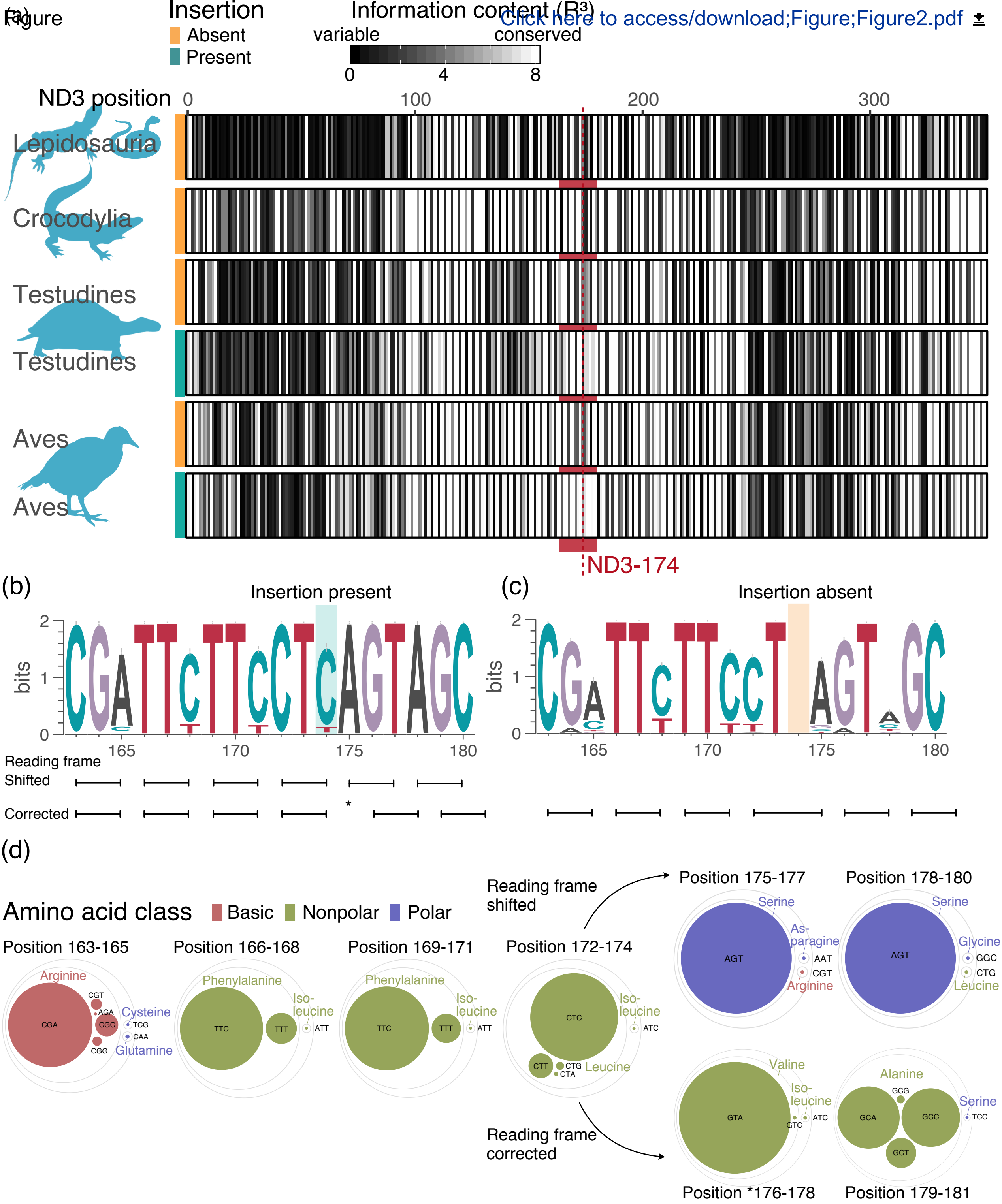

Click here to access/download
Supplementary Material
Additional_file_1.tsv

Click here to access/download
Supplementary Material
Additional_file_2.tsv

Click here to access/download
Supplementary Material
Additional_file_4.tsv

Click here to access/download
Supplementary Material
Additional_file_7.PNG

Click here to access/download
Supplementary Material
Additional_file_3.txt

Click here to access/download
Supplementary Material
Additional_file_5.pdf

Click here to access/download
Supplementary Material
Additional_file_6.pdf
