# Supplementary figures and images for: Multiple origins of a frameshift insertion in a mitochondrial gene in birds and turtles
Source: Gigascience. 2021 Jan 19;10(1):giaa161. doi: 10.1093/gigascience/giaa161 (PMC7814300; doi:10.1093/gigascience/giaa161)

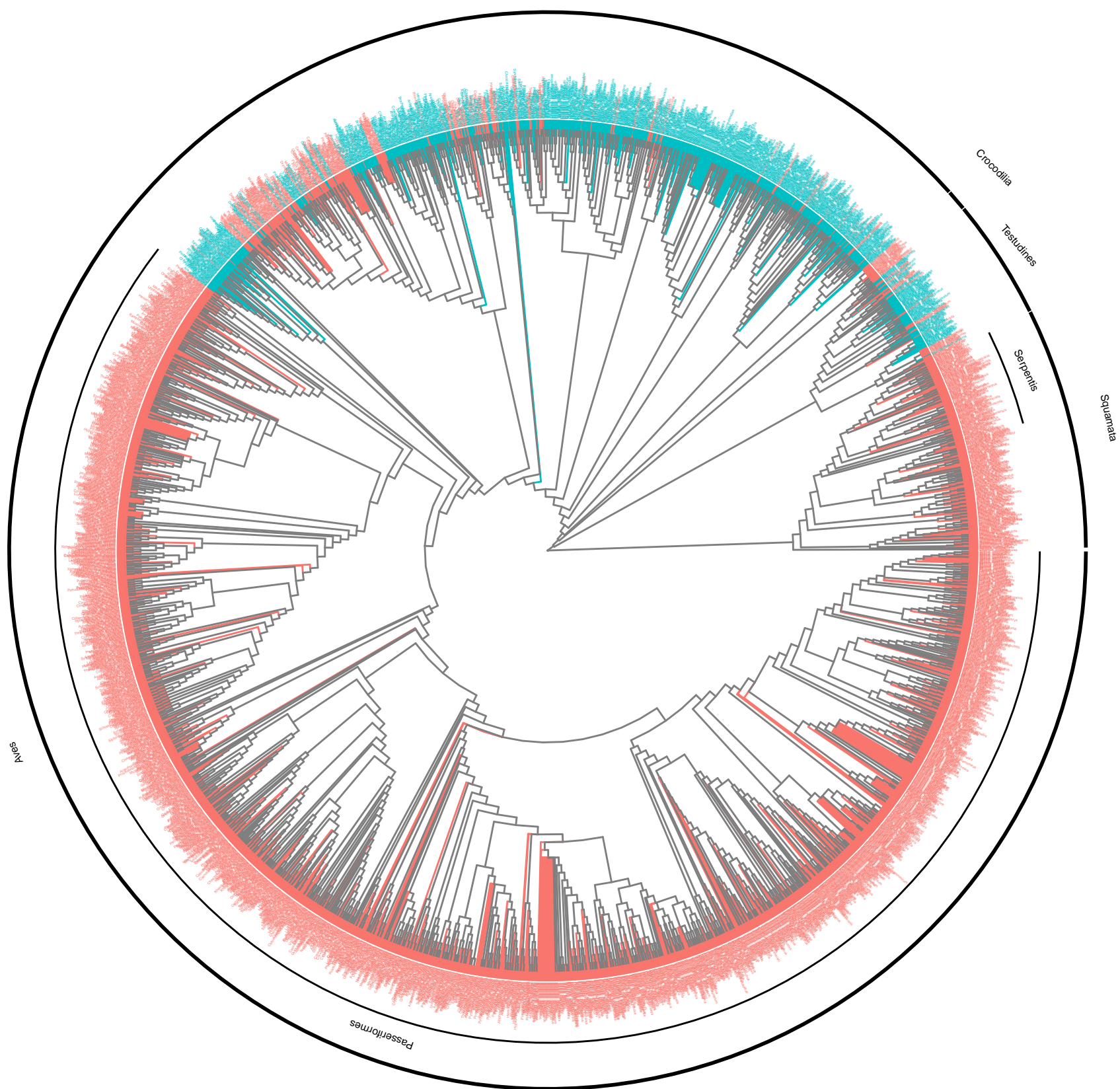

Status Position 174 — Absent — Present — NA

Supplement: giaa161_Supplemental_Files [file giaa161_supplemental_files.zip › Additional_file_5.pdf]

# Birds

Gap | Insertion

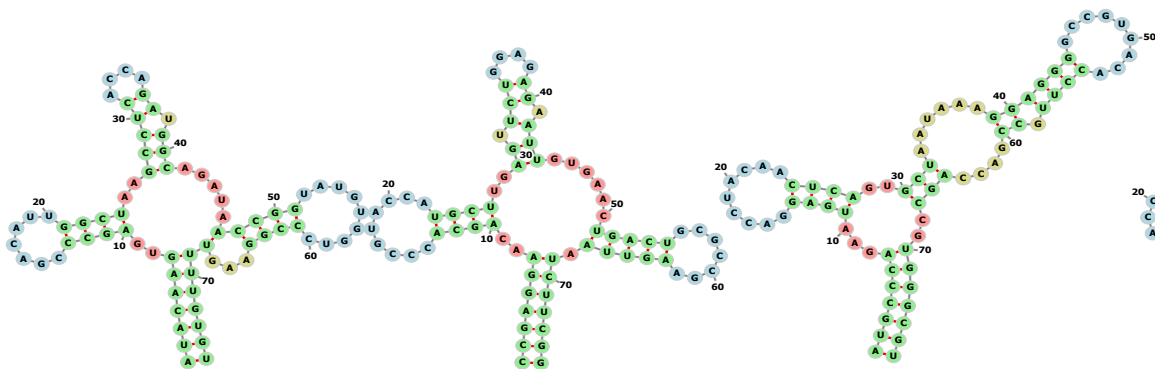

# Turtles

Gap | Insertion

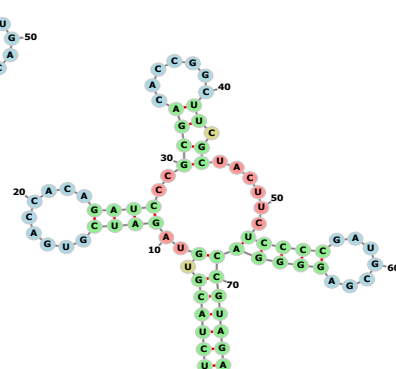

## Leu

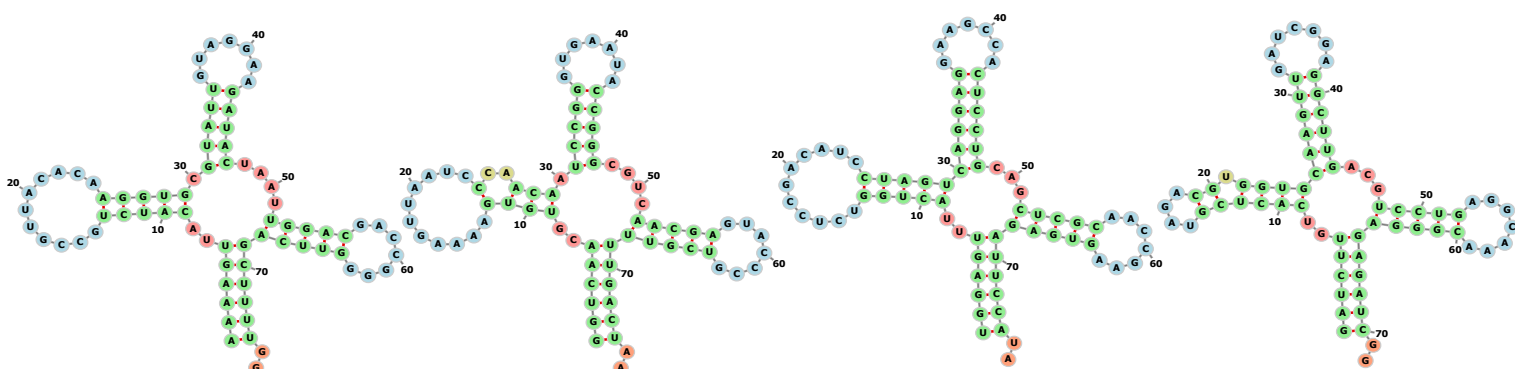

## Ser

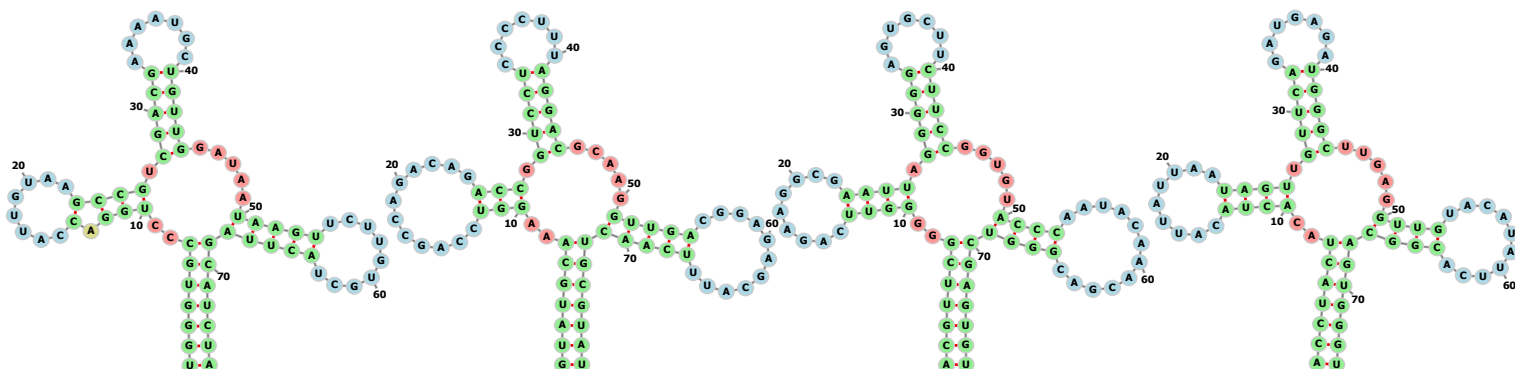

## Val

Supplement: giaa161_Supplemental_Files [file giaa161_supplemental_files.zip › Additional_file_6.pdf]

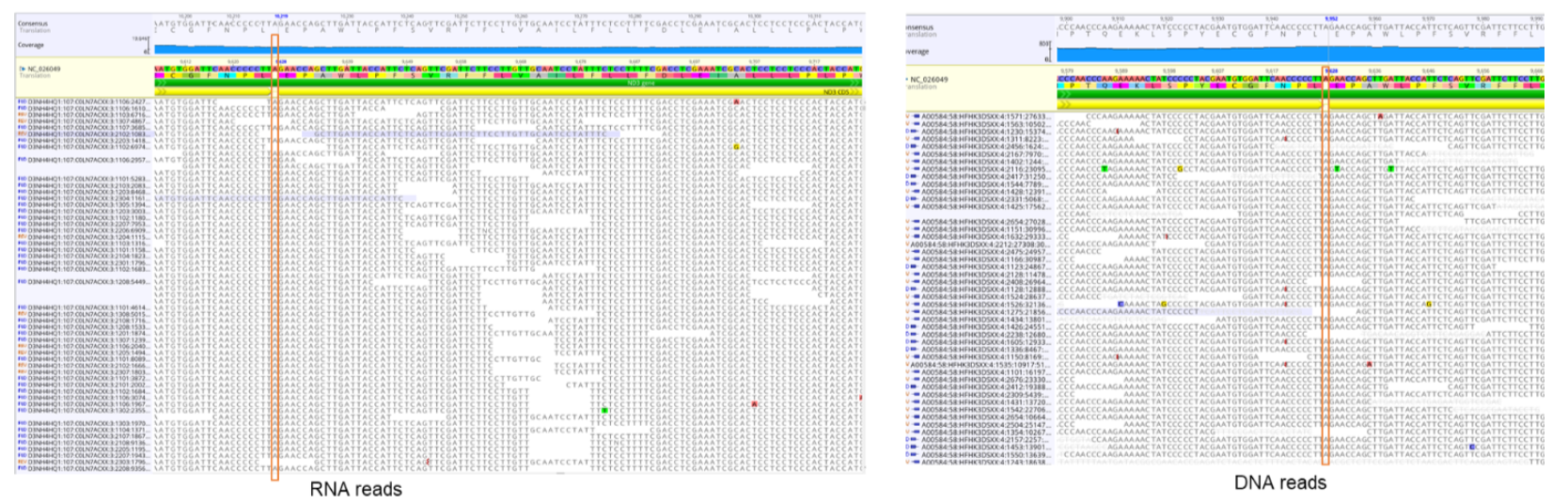

Supplement: giaa161_Supplemental_Files [file giaa161_supplemental_files.zip › Additional_file_7.PNG]
