# Supplementary material for: Multiple origins of a frameshift insertion in a mitochondrial gene in birds and turtles
Source: Gigascience. 2021 Jan 19;10(1):giaa161. doi: 10.1093/gigascience/giaa161 (PMC7814300; doi:10.1093/gigascience/giaa161)
Supplement: giaa161_Response_to_Reviewer_Comments_Original_Submission [file giaa161_response_to_reviewer_comments_original_submission.pdf]

Author's Response To Reviewer Comments

Close

Reviewer #1:

>>>We thank the reviewer for their constructive suggestions, which have improved the manuscript. We have addressed all suggestions in detail below.

Reviewer #1: The manuscript "Multiple origins of a frameshift insertion in a mitochondrial gene in birds and turtles" by Andreu-Sánchez and colleagues is a well-written and very interesting manuscript. I think it is an important contribution. Programmed frameshifts are actually reasonably common in viruses (Harger et al. 2002; Plant et al. 2005; Brierley & Dos Ramos 2006) but quite rare in cellular organisms (in fact, a -1 frameshift exists in coronaviruses; Plant et al. 2005). The authors correctly cite much of the literature, including the existence of some other programmed frameshifts in mitochondrially-encoded proteins in metazoa, although it would be nice if the authors could cite some additional literature, like the more recent review by Dinman (2012). I only have a few relatively minor comments.
>>>We have now cited the suggested reference (L. 109).

To my knowledge, after the original Mindell et al. (1998) study, the only previous study that has explicitly pointed out homoplasy in the frameshift was one from my lab (Tamashiro et al. 2019). In both cases the distribution of frameshifts could be explained by an ancient origin combined with multiple losses.

I also note that the authors state (on lines 315-317) that "Most of the ND3 sequences used here originate from Sanger sequenced ND3 genes, whose chromatograms may have been hand-curated for sequencing errors. Insertions at position 174 are [therefore] likely to be genuine…". The Tamashiro et al. (2019) study I alluded to used Illumina data and I can definitely attest to the presence of the insertion in many reads (and the absence in the three taxa in that study that lack the frameshift insertion). My lab group has also collected a number of Sanger ND3 sequences and they definitely have the insertion. Errors are certainly possible (indeed, the original Desjardins & Morais 1990 chicken mitogenome sequence almost certainly removed the frameshift, assuming it was a sequencing error - Mindell et al. 1998 states this and other Gallus mitogenome sequences - including some from my lab - have the frameshift).
>>>We apologize if our phrasing caused any confusion. Our point was that, in case of an observed absence of the insertion from sequences available on Genbank, the insertion could have been mistakenly removed if the curator was not aware that an insertion could have been present. It is possible that curators that are unaware of the Mindell et al. 1998 study and others will be more likely to remove the insertion. The absence of an insertion in Genbank sequences is therefore not necessarily the true state. On the other hand, an insertion that is present at position 174 in sequences available on Genbank is more likely to be genuine as it would have been flagged as a frameshift insertion during the sequence submission process. Thus, a present insertion is therefore more likely to be the true state.

I only have one conceptual issue with the analyses. I think it would be good to complement the likelihood with a parsimony analysis. The conservative nature of the way the authors calculated the number of transitions (i.e., only counting changes between ancestral state reconstructions when the contribution of a specific state to the marginal likelihoods was >0.9) is a double-edged sword. After all, there will be cases where it is clear transformations have occurred, but the exact branch where that change occurred is unclear. I think a very simple parsimony analysis would complement their likelihood analysis and provide a useful way of reporting the results. Although parsimony is often considered "old-fashioned" in the phylogenetic community it is simply the likelihood solution given the no common mechanism model (Steel & Penny 2000); I am not convinced that the simple stochastic model used by the authors is better for an still poorly-characterized type of evolutionary change (like shifts between the presence and absence of a programmed frameshift) than the no common mechanism (parsimony) model. Indeed, the absence of changes within passerines suggests that the rate of change for the

presence or absence of a frameshift is
>>>We thank the reviewer for this suggestion and we have incorporated a maximum parsimony analysis as implemented in the R ape package. The parsimony analysis largely corroborates the likelihood analysis. Most nodes that have a high probability of one state in the likelihood analysis are also resolved in the parsimony analysis. In the same vein, nodes with uncertainty in the maximum likelihood model were also not resolved in the parsimony analysis and had equally parsimonious states of absence/presence. Corresponding sections have been added to the Results in Sections: Patterns of presence and absence of ND3-174+1 in vertebrates, Complex patterns of gain and loss within turtles and birds and Materials and methods in Section:Ancestral state reconstruction.

From the standpoint of mechanics, all the authors need to do is created a nexus file with a single binary character the program PAUP* (Swofford 2020) and then map the character on the trees the authors used. They already have the character scorings, so this should be a trivial file format conversion. If the authors read the tree, start saving a log file, and then issue the command "DescribeTrees / chglist=yes plot=no;" (or choose the same options through the GUI), and then stop logging. The can then simply use grep to count numbers of 0 to 1 and 1 to 0 changes (PAUP* reports unambiguous and ambiguous mapping of the changes using ==> and -->). This will run quickly (even on a laptop computer) and authors can report numbers of changes given ACCTRAN and DELTRAN (Swofford & Maddison 1987). Changing between the two optimizations is accomplished by "PSet opt=delTran;" and "PSet opt=accTran;" (note that ACCTRAN is the default). This will be a simple and easy to interpret analysis that is insensitive to branch lengths. Assuming the authors add this simple analysis, they should also report the retention index (which is echoed when they do "DescribeTrees").

As stated above, one concern that leads me to suggest the parsimony analysis is the issue of branch lengths. I was unable to find how the authors obtained the branch lengths for their analysis. This should be clarified.
>>>We apologize that it was not explicitly stated. The used consensus phylogeny from the Open Tree of Life does not contain branch lengths. The model used assumed equal length for all branches. This is now clearly stated in on line 221.:
“Because the Open Tree of Life consensus phylogeny did not include branch lengths, the function assumes equal branch lengths throughout the phylogeny.”

A final comment about parsimony: although I would really like to see the parsimony analysis, I am not convinced a parsimony analysis with five-state coding (i.e., coding the identity of the base) is necessary. I am mostly interested in the parsimony estimate of gains vs losses in a binary sense. Obviously, if the authors are interested in doing the five-state parsimony analysis it would be fine, I just feel the presence vs absence analysis is more interesting to readers.
>>>We followed the suggestion and reconstructed the presence/absence of the insertion in the maximum parsimony framework.

On lines 430-431 the authors state "It remains unknown why the ND3 insertion appears in birds and turtles, and whether the occurrence of this insertion is under neutral change or subjected to natural selection." This is awkwardly phrased. It might be better as "It remains unknown whether the ND3 insertion has any functional in birds and turtles; the gain and loss of the inserted nucleotide could be neutral or it could be subject to selection." I would also say that the hypothesis that indel mutations are especially common at this site (articulated on lines 434-435 "One possibility is that there is an increased probability to produce indels in that specific position") is certainly possible but it seems very unlikely. The possibility that frameshifts are tolerated in this position is much more likely. In this context, it would seems likely that there is a change in passerines that does not allow frameshifting (of course, it is possible that some change in passerines has resulted in a lower rate new indels mutations at this position, but it seems much more likely that new indels are not tolerated at this position and therefore removed by natural selection).

>>>Fixed as suggested (L. 401):
”One possibility is that there is an increased probability to produce indels in that specific position. Alternatively, insertions may appear at a normal rate but only get tolerated if they are embedded in a specific sequence motif that allows ribosomes to conduct the frameshift correction. This seems likely given the strong conservation at the nucleotide and at the codon level that evolved convergently in birds and turtles, despite their separate evolution for over 240 million years.”

Finally, it would be a good idea for the authors to take more care with references. For example, on line 288 the authors use numbered references and, on line 320, "Mindell et al." is reiterated. Overall, a careful readthrough to catch these issues is warranted.

>>>Fixed.

Regardless of whether I provide a positive or negative review I like to sign my reviews (unless it is against journal policy). I believe the review process would be more positive and constructive if all reviews were either open or if they were double-blind; anonymous reviews create too much potential for reviews that are not constructive. Of course, it is much more pleasant to sign a review when I am enthusiastic about the manuscript, as I am here.
>>>We thank the reviewer for their constructive and positive comments, which have improved the quality of the manuscript.

Edward L. Braun

Reviewer #2:

>>>We thank the reviewer for their constructive and helpful comments. We appreciate the time and effort in scrutinizing the dataset and have addressed all suggestions and concerns in detail below.

Reviewer #2: The authors make use of a comprehensive mitogenomic dataset, covering a wide range of taxa among vertebrates, and later focusing on turtles and birds, to study the evolution of an insertion causing a frame-shift and an early stop codon in the mitochondrial ND3 gene. They identify it as occurring only in turtles and birds but being absent in Crocodilians and other groups of vertebrates. Using sequences for more than 9,000 taxa, they conduct a comparative phylogenetic approach and suggest that the insertion appeared at the ancestor of Archosauria and was later lost in Crocodilians as well as in multiple lineages of both birds and turtles. They also analyze the influence of base composition in the flanking regions of the insertion to examine their potential influence on the occurrence of the insertion and the mechanisms to cope with it.

The manuscript is general well written and easy to understand, even if some sections could be more concise and less redundant. It would be highly appreciated if the authors included some more information on the alternative mechanisms that have been proposed to deal with frame-shifts (see refs below).
>>>We thank the reviewer for the positive comments on the manuscript. We have paid careful attention in reducing redundancy throughout the manuscript.

The manuscript now mentions two other mechanisms other than programmed frameshifting with the relevant references (Line 113):
“Alternatively, other mechanisms could explain the absence of functional consequences of the frameshift insertion, such as non-canonical translation of tetra or penta codons, which might be an ancient translation mechanism [12], or RNA editing [13]. “
In addition, we also make a brief mention to the 3 translational frameshifts described in the introduction of Han et al (line 125):
“Three models have been proposed to enable translational frameshifting [13]. The “pause-and-slip” model proposes that a pause is induced at the A-site of the ribosome and that the P-site tRNA can pair with the +1 codon, allowing it to slip out of frame [15]. A second model proposes that abnormal tRNA structures enable the frameshift [16]. The “out-of-frame” model proposes that the recruited tRNA skips the additional nucleotide in the A-site [11].”

The methods section is in its current state well written and in detail described, the analytical pipeline is sound and the results seemed to support the conclusions presented by the authors.
For the discussion, however, I could not avoid the feeling that what they find is not different from what was already described in previous studies, which is a loss of the insertion in crocodilians and multiple losses and gains within turtles and birds. I would thus suggest, making use of the outstanding dataset the authors already compiled, the study to be extended to other insertions detected in the ND3 gene across vertebrates and, more particularly, in the taxa they focus, turtles and birds.
>>>We thank the reviewer for this suggestion. We have now studied the presence and absence of other insertions in ND3 in birds and turtles. We identified four frameshift insertions upstream of ND3-174 in five turtle species. Despite having many more available records from birds than from turtles, we did not detect any additional frameshifts in birds. This implies a widespread ability of coping with frameshifts in turtles, while translation frameshifting seems to be limited to ND3-174 in birds. Because of the limited occurrence of the other frameshifts, we focus the remaining manuscript on ND3-174. The corresponding section has been added to the manuscript, Section: Additional potential frameshifts in five turtle species

Before doing that, however, the authors need to go some stepes backwards and revise the alignment (provided in Additional file 1) as a close inspection of it led me to detected an issue that might require most of the analyses to be redone and the manuscript modified accordingly if the conclusions were to change.

First, the alignment needs to be improved to ensure that reading frame is respected along the full set of sequences along the whole gene. This is easily accomplished using a codon-aware aligner able to deal with frame-shifts. From my experience, the alternative proposed by Ranwez et al (2011) in MACSE (available at https://bioweb.supagro.inra.fr/macse/index.php?menu=releases) should be enough to correct this.
>>>We thank the reviewer for the suggestion of using a codon-aware aligner. Unfortunately, MACSE could not be used on the large alignment of 10,308 sequences across vertebrates used in our manuscript. We therefore decided to use MACSE on a smaller dataset across Diapsida (1,044 sequences). For the full Vertebrata alignment, we continued to use MAFFT but excluded sequences that could have resulted in spurious alignments around position 174. We filtered out sequences that were not properly aligned (e.g. large scale insertions or deletions which could be indicative of a numt or low-quality sequencing) or that had low quality in the region of interest (N in the codons surround position 174).

In order to assess the impact of the chosen aligner on the inference of the presence or absence of the insertion at ND3-174, we compared the prediction of presence/absence of the insertion at ND3-174 using the MACSE alignment of 1,044 Diapsida sequences with the MAFFT alignment of Diapsida derived from the Vertebrata alignment. We only observed different predictions in three species of a turtle genus, Cuora aurocapitata, Cuora pani and Cuora trifasciata. All three were predicted with no insertion by MACSE but with an insertion of T at position 174 in MAFFT. The MAFFT alignment shows a gap six base pairs upstream (see Reviewer 2 – Figures alignment A)

If the sequence was shifted to the left, the alignment would still be good and would remove the inferred insertion of T at position 174 (see screenshot below). We manually corrected this alignment: see Reviewer 2 – Figures alignment B

The choice of the aligner therefore did not influence much for the inference of the insertion or the inferred patterns of the gain and loss of the insertions. A heavier impact likely lies in the quality of the aligned sequences, which we have exposed to greater scrutiny now than in the previous iteration of the dataset.

Second, and more important, it is known for some species not having the insertion at position 174, that they can present a different one earlier in the sequence. An example of this, already presented in Russell & Beckenbach (2008), is the African helmeted turtle, Pelomedusa subrufa, which has not one but three different insertions, each on a different gene, including ND3.

When checking for this taxon, I noticed that (i) it is present twice in the alignment, likely due to the comma present in one of the labels, that likely made it escape the filtering to keep single sequences per taxon; perhaps this is also true for other taxa, thus adding redundant information ; (ii) this insertion is absent in the alignment and its incorrect filtering (maybe it is present in less than 95% of the sequences?) results in disrupted reading frames for the rest of the species.

In order to verify, I checked the mitogenome in Genbank (NC_001947.1), where the insertion is explicitly annotated as an exception and present in the annotated sequence (see captions of nucleotide and protein alignments in joint pdf file).
I consider that including it, along with the possibility that it happens elsewhere in the phylogeny, is susceptible to change the big picture of the evolution of insertions in this gene and should be addressed in this manuscript.

>>>We thank the reviewer for highlighting these problems with the included sequences. We have re-collected the dataset from scratch and have changed our strategy of collecting the sequence names to avoid the highlighted problems mentioned by the reviewer. For the previous dataset, we extracted species names from the Genbank headers but this was not always successful due to often non-standardized headers and resulted in artifacts such as the one described in (i). We now use NCBI's taxonomy to select unique sequences using the Organism field from the NCBI allowing for better control of taxon names. While this mostly corresponds to species names, it should be noted that NCBI's taxonomy sometimes includes placeholder taxa (e.g. species with cf. designation or non-formal names). We decided to include these placeholder taxa, which may be formally named at some point. This dataset is also more transparent as to which sequences are represented than our previous attempt to assign each sequence to a species.

For point ii), the absence of this particular frameshift was indeed caused by the filtering of positions not seen at least in 5% of sequences. We removed such singletons from the alignment to focus on position 174 but we acknowledge that this alignment alone is too simplified. We now include the unfiltered alignment Reviewer_2_alignment.txt (which will be uploaded to GigaDB) and the MACSE alignment where this insertion, and other possible frameshifts (Additional_File_4).

I think it would also be interesting some analyses on tRNAs, as coping with the frame-shifts described in this manuscript could be reflected in tRNA structures (see, for example, Haen et al (2014)). This option is not mentioned in the manuscript and given that some species present more than one insertion in coding genes along the mitogenome, it should definitively be considered. Given that a significant portion of the sequences in this study comes from fully assembled and annotated mitogenomes (e.g. RefSeq sequences from Genbank), having a look for differences in tRNAs sequences and structures should be feasible and would provide more value and support to the inferred trends and observations.
>>>This is an excellent suggestion. We have included an analysis of tRNA secondary structures in the codons surrounding the insertion. We compared secondary structures for birds and turtles with and without the insertion, respectively. We focused on the secondary structures of tRNAs of the codon encompassing the insertion (leucine) and the following tRNA, which is serine when the frameshift is not corrected and valine if the frameshift is corrected. The secondary structures between these groups were not noticeably different between the groups, which indicates that there are no convergent modifications to the tRNA secondary structures between birds and turtles with the insertion. The predicted secondary structures are included as Additional_file_6 and alignments will be made publicly available in GigaDB. The analyses are described in the Methods (Section:tRNA secondary structure prediction) and Results (Section: No major tRNA changes in taxa presenting the insertion).

Close
