# Supplementary material for: Multiple origins of a frameshift insertion in a mitochondrial gene in birds and turtles
Source: Gigascience. 2021 Jan 19;10(1):giaa161. doi: 10.1093/gigascience/giaa161 (PMC7814300; doi:10.1093/gigascience/giaa161)
Supplement: giaa161_Response_to_Reviewer_Comments_Revision_1 [file giaa161_response_to_reviewer_comments_revision_1.pdf]

Author's Response To Reviewer Comments

Close

We are glad to comply with the last suggestion of the reviewer and to edit the header of the section he refers to.
At the same time, we have now included information about the GigaDB repository.

Close
