# Supplementary material for: Multiple origins of a frameshift insertion in a mitochondrial gene in birds and turtles
Source: Gigascience. 2021 Jan 19;10(1):giaa161. doi: 10.1093/gigascience/giaa161 (PMC7814300; doi:10.1093/gigascience/giaa161)
Supplement: giaa161_Reviewer_1_Report_Original_Submission — Edward Louis Braun, Ph.D. -- 5/24/2020 Reviewed [file giaa161_reviewer_1_report_original_submission.pdf]

Reviewer Report

Title: Multiple origins of a frameshift insertion in a mitochondrial gene in birds and turtles

Version: Original Submission **Date:** 5/24/2020

Reviewer name: Edward Louis Braun, Ph.D.

Reviewer Comments to Author:

The manuscript "Multiple origins of a frameshift insertion in a mitochondrial gene in birds and turtles" by Andreu-Saì�nchez and colleagues is a well-written and very interesting manuscript. I think it is an important contribution. Programmed frameshifts are actually reasonably common in viruses (Harger et al. 2002; Plant et al. 2005; Brierley & Dos Ramos 2006) but quite rare in cellular organisms (in fact, a -1 frameshift exists in coronaviruses; Plant et al. 2005). The authors correctly cite much of the literature, including the existence of some other programmed frameshifts in mitochondrially-encoded proteins in metazoa, although it would be nice if the authors could cite some additional literature, like the more recent review by Dinman (2012). I only have a few relatively minor comments.

To my knowledge, after the original Mindell et al. (1998) study, the only previous study that has explicitly pointed out homoplasy in the frameshift was one from my lab (Tamashiro et al. 2019). In both cases the distribution of frameshifts could be explained by an ancient origin combined with multiple losses.

I also note that the authors state (on lines 315-317) that "Most of the ND3 sequences used here originate from Sanger sequenced ND3 genes, whose chromatograms may have been hand-curated for sequencing errors. Insertions at position 174 are [therefore] likely to be genuine…". The Tamashiro et al. (2019) study I alluded to used Illumina data and I can definitely attest to the presence of the insertion in many reads (and the absence in the three taxa in that study that lack the frameshift insertion). My lab group has also collected a number of Sanger ND3 sequences and they definitely have the insertion. Errors are certainly possible (indeed, the original Desjardins & Morais 1990 chicken mitogenome sequence almost certainly removed the frameshift, assuming it was a sequencing error - Mindell et al. 1998 states this and other Gallus mitogenome sequences - including some from my lab - have the frameshift).

I only have one conceptual issue with the analyses. I think it would be good to complement the likelihood with a parsimony analysis. The conservative nature of the way the authors calculated the number of transitions (i.e., only counting changes between ancestral state reconstructions when the contribution of a specific state to the marginal likelihoods was >0.9) is a double-edged sword. After all, there will be cases where it is clear transformations have occurred, but the exact branch where that change occurred is unclear. I think a very simple parsimony analysis would complement their likelihood analysis and provide a useful way of reporting the results. Although parsimony is often considered "old-fashioned" in the phylogenetic community it is simply the likelihood solution given the no common mechanism model (Steel & Penny 2000); I am not convinced that the simple stochastic model used by the authors is better for an still poorly-characterized type of evolutionary change (like shifts between the presence and absence of a programmed frameshift) than the no common mechanism (parsimony)

model. Indeed, the absence of changes within passerines suggests that the rate of change for the presence or absence of a frameshift is

From the standpoint of mechanics, all the authors need to do is created a nexus file with a single binary character the program PAUP* (Swofford 2020) and then map the character on the trees the authors used. They already have the character scorings, so this should be a trivial file format conversion. If the authors read the tree, start saving a log file, and then issue the command "DescribeTrees / chglist=yes plot=no;" (or choose the same options through the GUI), and then stop logging. The can then simply use grep to count numbers of 0 to 1 and 1 to 0 changes (PAUP* reports unambiguous and ambiguous mapping of the changes using ==> and -->). This will run quickly (even on a laptop computer) and authors can report numbers of changes given ACCTRAN and DELTRAN (Swofford & Maddison 1987). Changing between the two optimizations is accomplished by "PSet opt=delTran;" and "PSet opt=accTran;" (note that ACCTRAN is the default). This will be a simple and easy to interpret analysis that is insensitive to branch lengths. Assuming the authors add this simple analysis, they should also report the retention index (which is echoed when they do "DescribeTrees").

As stated above, one concern that leads me to suggest the parsimony analysis is the issue of branch lengths. I was unable to find how the authors obtained the branch lengths for their analysis. This should be clarified.

A final comment about parsimony: although I would really like to see the parsimony analysis, I am not convinced a parsimony analysis with five-state coding (i.e., coding the identity of the base) is necessary. I am mostly interested in the parsimony estimate of gains vs losses in a binary sense. Obviously, if the authors are interested in doing the five-state parsimony analysis it would be fine, I just feel the presence vs absence analysis is more interesting to readers.

On lines 430-431 the authors state "It remains unknown why the ND3 insertion appears in birds and turtles, and whether the occurrence of this insertion is under neutral change or subjected to natural selection." This is awkwardly phrased. It might be better as "It remains unknown whether the ND3 insertion has any functional in birds and turtles; the gain and loss of the inserted nucleotide could be neutral or it could be subject to selection." I would also say that the hypothesis that indel mutations are especially common at this site (articulated on lines 434-435 "One possibility is that there is an increased probability to produce indels in that specific position") is certainly possible but it seems very unlikely. The possibility that frameshifts are tolerated in this position is much more likely. In this context, it would seems likely that there is a change in passerines that does not allow frameshifting (of course, it is possible that some change in passerines has resulted in a lower rate new indels mutations at this position, but it seems much more likely that new indels are not tolerated at this position and therefore removed by natural selection).

Finally, it would be a good idea for the authors to take more care with references. For example, on line 288 the authors use numbered references and, on line 320, "Mindell et al." is reiterated. Overall, a careful readthrough to catch these issues is warranted.

Regardless of whether I provide a positive or negative review I like to sign my reviews (unless it is against journal policy). I believe the review process would be more positive and constructive if all reviews were either open or if they were double-blind; anonymous reviews create too much potential for reviews that are not constructive. Of course, it is much more pleasant to sign a review when I am enthusiastic about the manuscript, as I am here.

Edward L. Braun

References for this review:

Brierley, I., & Dos Ramos, F. J. (2006). Programmed ribosomal frameshifting in HIV-1 and the SARS-CoV. Virus research, 119(1), 29-42. doi: 10.1016/j.virusres.2005.10.008

Desjardins, P., & Morais, R. (1990). Sequence and gene organization of the chicken mitochondrial genome: a novel gene order in higher vertebrates. Journal of molecular biology, 212(4), 599-634. doi: 10.1016/0022-2836(90)90225-B

Dinman, J. D. (2012). Mechanisms and implications of programmed translational frameshifting. Wiley Interdisciplinary Reviews: RNA, 3(5), 661-673. doi: 10.1002/wrna.1126

Harger, J. W., Meskauskas, A., & Dinman, J. D. (2002). An 'integrated model'of programmed ribosomal frameshifting. Trends in biochemical sciences, 27(9), 448-454. doi: 10.1016/S0968-0004(02)02149-7

Mindell, D. P., Sorenson, M. D., & Dimcheff, D. E. (1998). An extra nucleotide is not translated in mitochondrial ND3 of some birds and turtles. Molecular Biology and Evolution, 15(11), 1568-1571. doi: 10.1093/oxfordjournals.molbev.a025884

Plant, E. P., PÃ©rez-Alvarado, G. C., Jacobs, J. L., Mukhopadhyay, B., Hennig, M., & Dinman, J. D. (2005). A three-stemmed mRNA pseudoknot in the SARS coronavirus frameshift signal. PLoS biology, 3(6), e172. doi: 10.1371/journal.pbio.0030172

Steel, M., & Penny, D. (2000). Parsimony, likelihood, and the role of models in molecular phylogenetics. Molecular biology and evolution, 17(6), 839-850. doi:

Swofford, D. L. (2020). PAUP* (Phylogenetic analyses using parsimony (*and other methods) v4.0a167. Available from https://paup.phylosolutions.com/

Swofford, D. L., & Maddison, W. P. (1987). Reconstructing ancestral character states under Wagner parsimony. Mathematical Biosciences, 87(2), 199-229. doi: 10.1016/0025-5564(87)90074-5

Tamashiro, R. A., White, N. D., Braun, M. J., Faircloth, B. C., Braun, E. L., & Kimball, R. T. (2019). What are the roles of taxon sampling and model fit in tests of cyto-nuclear discordance using avian mitogenomic data? Molecular phylogenetics and evolution, 130, 132-142. doi: 10.1016/j.ympev.2018.10.008

Methods

Are the methods appropriate to the aims of the study, are they well described, and are necessary controls included? Choose an item.

Conclusions

Are the conclusions adequately supported by the data shown? Choose an item.

Reporting Standards

Does the manuscript adhere to the journal's guidelines on [minimum standards of reporting?]() Choose an item.

Choose an item.

Statistics

Are you able to assess all statistics in the manuscript, including the appropriateness of statistical tests used? Choose an item.

Quality of Written English

Please indicate the quality of language in the manuscript: Choose an item.

Declaration of Competing Interests

Please complete a declaration of competing interests, considering the following questions:

- Have you in the past five years received reimbursements, fees, funding, or salary from an organisation that may in any way gain or lose financially from the publication of this manuscript, either now or in the future?
- Do you hold any stocks or shares in an organisation that may in any way gain or lose financially from the publication of this manuscript, either now or in the future?
- Do you hold or are you currently applying for any patents relating to the content of the manuscript?
- Have you received reimbursements, fees, funding, or salary from an organization that holds or has applied for patents relating to the content of the manuscript?
- Do you have any other financial competing interests?
- Do you have any non-financial competing interests in relation to this paper?

If you can answer no to all of the above, write 'I declare that I have no competing interests' below. If your reply is yes to any, please give details below.

I declare that I have no competing financial interests. I have collaborated with the corresponding author in the past and I also have some ongoing collaborations. I believe that I am able to be objective in this review .

I agree to the open peer review policy of the journal. I understand that my name will be included on my report to the authors and, if the manuscript is accepted for publication, my named report including any attachments I upload will be posted on the website along with the authors' responses. I agree for my report to be made available under an Open Access Creative Commons CC-BY license (http://creativecommons.org/licenses/by/4.0/). I understand that any comments which I do not wish to be included in my named report can be included as confidential comments to the editors, which will not be published.

Choose an item.

To further support our reviewers, we have joined with Publons, where you can gain additional credit to further highlight your hard work (see: https://publons.com/journal/530/gigascience). On publication of this paper, your review will be automatically added to Publons, you can then choose whether or not to claim your Publons credit. I understand this statement.

Yes Choose an item.
