# Supplementary material for: Multiple origins of a frameshift insertion in a mitochondrial gene in birds and turtles
Source: Gigascience. 2021 Jan 19;10(1):giaa161. doi: 10.1093/gigascience/giaa161 (PMC7814300; doi:10.1093/gigascience/giaa161)
Supplement: giaa161_Reviewer_1_Report_Revision_1 — Edward Louis Braun, Ph.D. -- 11/16/2020 Reviewed [file giaa161_reviewer_1_report_revision_1.pdf]

Reviewer Report

Title: Multiple origins of a frameshift insertion in a mitochondrial gene in birds and turtles

Version: Revision 1 **Date:** 11/16/2020

Reviewer name: Edward Louis Braun, Ph.D.

Reviewer Comments to Author:

I have read the revised manuscript carefully. and. feel that the authors took great pains to respond to all of my comments and all of the comments made by the second reviewer. I feel that all of my comments were addressed extremely well. Likewise, I was delighted to read the new material on tRNA structures, which the authors added to address a comment made by the second reviewer. Although the results were negative in the sense that they did not find clear structural changes, those negative results are still important. I have one minor comment about the title of that section (line 317) - it is "No major tRNA changes in taxa presenting the insertion" but I found that section title awkward; I would recommend the simpler "No major tRNA changes in taxa with the insertion". I was also delighted to see the section on the additional frameshifts in Pelomedusidae.
I really like this manuscript and I hope to see it in print. Other than suggesting that the reviewers consider my minor comment on line 317 (which is just a judgement call) I think this should be accepted as is. -Edward L. Braun

Methods

Are the methods appropriate to the aims of the study, are they well described, and are necessary controls included? Choose an item.

Conclusions

Are the conclusions adequately supported by the data shown? Choose an item.

Reporting Standards

Does the manuscript adhere to the journal's guidelines on [minimum standards of reporting?] Choose an item.

Choose an item.

Statistics

Are you able to assess all statistics in the manuscript, including the appropriateness of statistical tests used? Choose an item.

Quality of Written English

Please indicate the quality of language in the manuscript: Choose an item.

Declaration of Competing Interests

Please complete a declaration of competing interests, considering the following questions:

- Have you in the past five years received reimbursements, fees, funding, or salary from an organisation that may in any way gain or lose financially from the publication of this manuscript, either now or in the future?
- Do you hold any stocks or shares in an organisation that may in any way gain or lose financially from the publication of this manuscript, either now or in the future?
- Do you hold or are you currently applying for any patents relating to the content of the manuscript?
- Have you received reimbursements, fees, funding, or salary from an organization that holds or has applied for patents relating to the content of the manuscript?
- Do you have any other financial competing interests?
- Do you have any non-financial competing interests in relation to this paper?

If you can answer no to all of the above, write 'I declare that I have no competing interests' below. If your reply is yes to any, please give details below.

I have collaborated with the authors of this paper. However, I have no financial conflicts of interest related to this manuscript. I do not believe that I have any competing interests that would affect my evaluation of the manuscript.

I agree to the open peer review policy of the journal. I understand that my name will be included on my report to the authors and, if the manuscript is accepted for publication, my named report including any attachments I upload will be posted on the website along with the authors' responses. I agree for my report to be made available under an Open Access Creative Commons CC-BY license (http://creativecommons.org/licenses/by/4.0/). I understand that any comments which I do not wish to be included in my named report can be included as confidential comments to the editors, which will not be published.

Choose an item.

To further support our reviewers, we have joined with Publons, where you can gain additional credit to further highlight your hard work (see: https://publons.com/journal/530/gigascience). On publication of this paper, your review will be automatically added to Publons, you can then choose whether or not to claim your Publons credit. I understand this statement.

Yes Choose an item.
