# Supplementary material for: Multiple origins of a frameshift insertion in a mitochondrial gene in birds and turtles
Source: Gigascience. 2021 Jan 19;10(1):giaa161. doi: 10.1093/gigascience/giaa161 (PMC7814300; doi:10.1093/gigascience/giaa161)
Supplement: giaa161_Reviewer_2_Report_Original_Submission — Fidel Botero-Castro -- 6/23/2020 Reviewed [file giaa161_reviewer_2_report_original_submission.pdf]

Reviewer Report

Title: Multiple origins of a frameshift insertion in a mitochondrial gene in birds and turtles

Version: Original Submission **Date:** 6/23/2020

Reviewer name: Fidel Botero-Castro

Reviewer Comments to Author:

The authors make use of a comprehensive mitogenomic dataset, covering a wide range of taxa among vertebrates, and later focusing on turtles and birds, to study the evolution of an insertion causing a frame-shift and an early stop codon in the mitochondrial ND3 gene. They identify it as occurring only in turtles and birds but being absent in Crocodilians and other groups of vertebrates. Using sequences for more than 9,000 taxa, they conduct a comparative phylogenetic approach and suggest that the insertion appeared at the ancestor of Archosauria and was later lost in Crocodilians as well as in multiple lineages of both birds and turtles. They also analyze the influence of base composition in the flanking regions of the insertion to examine their potential influence on the occurrence of the insertion and the mechanisms to cope with it.

The manuscript is general well written and easy to understand, even if some sections could be more concise and less redundant. It would be highly appreciated if the authors included some more information on the alternative mechanisms that have been proposed to deal with frame-shifts (see refs below).

The methods section is in its current state well written and in detail described, the analytical pipeline is sound and the results seemed to support the conclusions presented by the authors.

For the discussion, however, I could not avoid the feeling that what they find is not different from what was already described in previous studies, which is a lost of the insertion in crocodilians and multiple losses and gains within turtles and birds. I would thus suggest, making use of the outstanding dataset the authors already compiled, the study to be extended to other insertions detected in the ND3 gene across vertebrates and, more particularly, in the taxa they focus, turtles and birds.

Before doing that, however, the authors need to go some stepes backwards and revise the alignment (provided in Additional file 1) as a close inspection of it led me to detected an issue that might require most of the analyses to be redone and the manuscript modified accordingly if the conclusions were to change.

First, the alignment needs to be improved to ensure that reading frame is respected along the full set of sequences along the whole gene. This is easily accomplished using a codon-aware aligner able to deal with frame-shifts. From my experience, the alternative proposed by Ranwez et al (2011) in MACSE (available at https://bioweb.supagro.inra.fr/macse/index.php?menu=releases) should be enough to correct this.

Second, and more important, it is known for some species not having the insertion at position 174, that they can present a different one earlier in the sequence. An example of this, already presented in Russell & Beckenbach (2008), is the African helmeted turtle, Pelomedusa subrufa, which has not one but three different insertions, each on a different gene, including ND3.

When checking for this taxon, I noticed that (i) it is present twice in the alignment, likely due to the comma present in one of the labels, that likely made it escape the filtering to keep single sequences per taxon; perhaps this is also true for other taxa, thus adding redundant information ; (ii) this insertion is absent in the alignment and its incorrect filtering (maybe it is present in less than 95% of the sequences?) results in disrupted reading frames for the rest of the species.
In order to verify, I checked the mitogenome in Genbank (NC_001947.1), where the insertion is explicitly annotated as an exception and present in the annotated sequence (see captions of nucleotide and protein alignments in joint pdf file).
I consider that including it, along with the possibility that it happens elsewhere in the phylogeny, is susceptible to change the big picture of the evolution of insertions in this gene and should be addressed in this manuscript.
I think it would also be interesting some analyses on tRNAs, as coping with the frame-shifts described in this manuscript could be reflected in tRNA structures (see, for example, Haen et al (2014)). This option is not mentioned in the manuscript and given that some species present more than one insertion in coding genes along the mitogenome, it should definitively be considered. Given that a significant portion of the sequences in this study comes from fully assembled and annotated mitogenomes (e.g. RefSeq sequences from Genbank), having a look for differences in tRNAs sequences and structures should be feasible and would provide more value and support to the inferred trends and observations.
References on mechanisms to cope with frameshifts
Seligmann, H., Warthi, G., 2019. Chimeric Translation for Mitochondrial Peptides: Regular and Expanded Codons. Comput Struct Biotechnol J., 17: 1195-1202.
Haen, K.M, Walker, P., Lavrov. D.V., 2014. Eight new mtDNA sequences of glass sponges reveal an extensive usage of +1 frameshifting in mitochondrial translation. Gene, 535: 336-344.

Methods

Are the methods appropriate to the aims of the study, are they well described, and are necessary controls included? Choose an item.

Conclusions

Are the conclusions adequately supported by the data shown? Choose an item.

Reporting Standards

Does the manuscript adhere to the journal’s guidelines on minimum standards of reporting? Choose an item.

Choose an item.

Statistics

Are you able to assess all statistics in the manuscript, including the appropriateness of statistical tests used? Choose an item.

Quality of Written English

Please indicate the quality of language in the manuscript: Choose an item.

Declaration of Competing Interests

Please complete a declaration of competing interests, considering the following questions:

- Have you in the past five years received reimbursements, fees, funding, or salary from an organisation that may in any way gain or lose financially from the publication of this manuscript, either now or in the future?
- Do you hold any stocks or shares in an organisation that may in any way gain or lose financially from the publication of this manuscript, either now or in the future?
- Do you hold or are you currently applying for any patents relating to the content of the manuscript?
- Have you received reimbursements, fees, funding, or salary from an organization that holds or has applied for patents relating to the content of the manuscript?
- Do you have any other financial competing interests?
- Do you have any non-financial competing interests in relation to this paper?

If you can answer no to all of the above, write 'I declare that I have no competing interests' below. If your reply is yes to any, please give details below.

I declare that I have no competing interests.

I agree to the open peer review policy of the journal. I understand that my name will be included on my report to the authors and, if the manuscript is accepted for publication, my named report including any attachments I upload will be posted on the website along with the authors' responses. I agree for my report to be made available under an Open Access Creative Commons CC-BY license (http://creativecommons.org/licenses/by/4.0/). I understand that any comments which I do not wish to be included in my named report can be included as confidential comments to the editors, which will not be published.

Choose an item.

To further support our reviewers, we have joined with Publons, where you can gain additional credit to further highlight your hard work (see: https://publons.com/journal/530/gigascience). On publication of this paper, your review will be automatically added to Publons, you can then choose whether or not to claim your Publons credit. I understand this statement.

Yes Choose an item.
